# From the Optic Neuritis Treatment Trial to Antibody-Mediated Optic Neuritis: Four Decades of Progress and Unanswered Questions

**DOI:** 10.3390/biomedicines14020334

**Published:** 2026-01-31

**Authors:** Marco A. Lana-Peixoto, Natália C. Talim, Paulo P. Christo

**Affiliations:** CIEM MS Research Center, Minas Gerais Medical School, Federal University, Belo Horizonte 30130-090, MG, Brazil; talim.fono@gmail.com (N.C.T.); ppchristo@gmail.com (P.P.C.)

**Keywords:** optic neuritis, Optic Neuritis Treatment Trial, MS-associated optic neuritis, AQP4-associated optic neuritis, MOG-associated optic neuritis, double-negative optic neuritis, optical coherence tomography, MRI biomarkers, CSF biomarkers

## Abstract

Optic neuritis (ON) has been recognized since antiquity, but its modern clinical identity emerged only in the late 19th century and was definitively shaped by the Optic Neuritis Treatment Trial (ONTT). The ONTT established the natural history, visual prognosis, association with multiple sclerosis (MS), and therapeutic response to corticosteroids, building the foundation for contemporary ON management. Subsequent discoveries—most notably aquaporin-4 IgG-associated ON (AQP4-ON), myelin oligodendrocyte glycoprotein antibody-associated ON (MOG-ON), and double-negative ON—have fundamentally transformed this paradigm, shifting ON from a seemingly uniform demyelinating syndrome to a group of biologically distinct disorders. These subtypes differ in immunopathology, clinical course, MRI features, retinal injury patterns, CSF profiles, and long-term outcomes, making early and accurate differentiation essential. MRI provides key distinctions in lesion length, orbital tissue inflammation, bilateral involvement, and chiasmal or optic tract extension. Optical coherence tomography (OCT) offers complementary structural biomarkers, including severe early ganglion cell loss in AQP4-ON, relative preservation in MOG-ON, and variable patterns in double-negative ON. CSF analysis further refines diagnosis, with oligoclonal bands strongly supporting MS-ON. Together, these modalities enable precise early stratification and timely initiation of targeted immunotherapy, which is critical for preventing irreversible visual disability. Despite major advances, significant unmet needs persist. Access to high-resolution MRI, OCT, cell-based antibody assays, and evidence-based treatments remains limited in many regions, contributing to global disparities in outcomes. The understanding of the pathogenesis of double-negative optic neuritis, the identification of reliable biomarkers of relapse and visual recovery, and the determination of standardized cut-off values for multimodal diagnostic tools—including MRI, OCT, CSF analysis, and serological assays—remain unresolved challenges. Future research must expand biomarker discovery, refine imaging criteria, and ensure equitable global access to cutting-edge diagnostic platforms and therapeutic innovations. Four decades after the ONTT, ON remains a dynamic field of investigation, with ongoing advances holding the potential to transform care for patients worldwide. Together, these advances expose a fundamental tension between historically MS-centered diagnostic frameworks and the emerging biological heterogeneity of ON, a tension that underpins the structure and critical perspective of the present review.

## 1. Introduction

Optic neuritis (ON) encompasses a spectrum of inflammatory disorders affecting the optic nerve, typically presenting with subacute visual loss. The conceptual origins of ON can be traced back to classical antiquity, specifically the 5th to 4th centuries BCE, when early physicians such as Alcmaeon of Croton and Hippocrates first proposed that vision loss could originate from neural structures beyond the eye itself [1]. Since then, the concept has evolved through a series of major milestones, most notably the invention of the ophthalmoscope in the mid-19th century, allowing the in vivo examination of optic nerve inflammation; the Optic Neuritis Treatment Trial (ONTT) studies with onset in the turn of the 20th century; and the identification of aquaporin 4 (AQP4 IgG) and myelin oligodendrocyte glycoprotein (MOG IgG) antibodies as causes of immune-mediated ON [2,3,4].

The difficulty in defining ON over more than two millennia reflects the way its underlying concepts evolved across history. Explanations for vision loss alternated between ocular, neural, inflammatory, and systemic models, creating lasting diagnostic uncertainties that persisted into the modern era. Appreciating these historical limitations is essential for understanding the ONTT—not only as a landmark achievement but also as a study shaped by the conceptual boundaries of its time.

Recently, an international consortium of experts proposed a classification framework for ON based on its underlying etiology and fundamental pathophysiological mechanisms. This initiative also introduced a set of diagnostic criteria that integrates clinical phenotype, neuroimaging features, and serological biomarkers, aiming to enhance diagnostic accuracy, inform prognosis, and guide therapeutic decision-making [5].

Despite these marked advancements, several unresolved issues persist, particularly regarding biomarker development, pathophysiological characterization, and long-term management of double-seronegative ON (DN-ON), defined as AQP4-IgG–negative, MOG-IgG–negative ON not associated with multiple sclerosis (MS). Importantly, the absence of validated biological markers means that DN-ON currently represents a heterogeneous, exclusion-based diagnostic category rather than a unified nosological entity. This limitation underscores the need for cautious interpretation of this group and highlights the importance of targeted research strategies aimed at refining classification, improving diagnostic precision, and guiding individualized therapeutic decision-making. This review provides a comprehensive synthesis of the evolution of the foundation of the ON concept from Antiquity to Modern Age, and the current status of its knowledge. We examine the state-of-the-art of immune-mediated ON including its clinical characteristics, diagnostic approach, and therapeutic strategies for both typical ON (MS-ON)—as defined by the ONTT and subsequent studies—and atypical ON (non-MS-ON), which predominantly includes AQP4-ON, MOG-ON, and double-seronegative forms of undetermined etiology. Additionally, we explore the major challenges associated with the diagnosis and subclassification of seronegative ON and propose research strategies aimed at defining distinct entities within this group. These include detailed analysis of clinical phenotyping, advanced neuroimaging techniques, and the identification of novel serum autoantibodies that could allow discrimination of other types of ON within this common group. Such efforts are essential for bridging current gaps in the field, ultimately enabling more precise diagnosis, targeted therapy, personalized care and improved clinical outcomes [5,6,7]. Against this historical backdrop, the following section reviews the major milestones that shaped the early conceptual foundations of optic neuritis.

## 2. Optic Neuritis Before the ONTT: Historical Foundations

**Figure 1:**
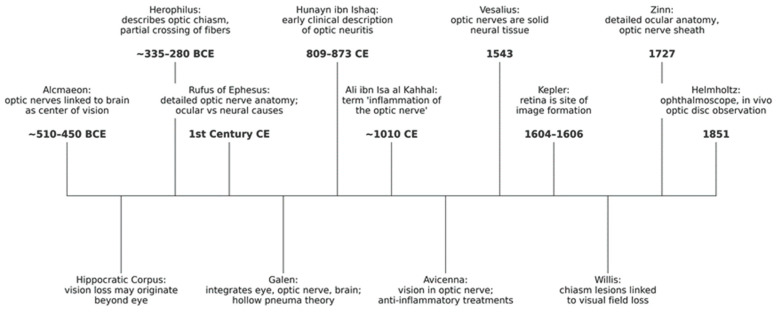
Milestones in the understanding of optic neuritis from antiquity to the onset of the modern era.

The conceptual foundations of optic neuritis (ON) emerged through a long sequence of anatomical, physiological, and clinical insights that progressively reframed vision from a purely ocular phenomenon to a neurological function. The principal milestones that shaped this evolution—from antiquity to the threshold of the modern era—are summarized in Figure 1.

### 2.1. Antiquity: Neural Vision and the Optic Pathways

The discovery of the optic nerves is traditionally attributed to Alcmaeon of Croton (c. 510–450 BCE), who traced visual pathways from the eyes to the brain and proposed that the brain, rather than the heart, was the seat of sensation and cognition [8]. This marked a decisive departure from earlier cardiocentric and metaphysical views of vision. Subsequent writings in the Hippocratic Corpus acknowledged visual loss without visible ocular disease, often described as *amaurosis*, although these texts did not clearly distinguish optic nerve pathology from intraocular disorders [9].

Anatomical understanding advanced further with Herophilus of Chalcedon (c. 335–280 BCE), who provided the first description of the optic chiasm as a crossing of the optic nerves [1]. Rufus of Ephesus (1st century CE) later recognized that visual loss could arise from central or neural causes and described the optic nerve as a solid structure extending from the brain to the eye, correcting earlier extramission theories [10]. Galen subsequently emphasized the coordinated role of the eye, optic nerves, and brain in vision, although his enduring doctrine still conceived the optic nerves as hollow conduits transmitting “visual pneuma,” an idea that dominated medical thought for more than a millennium [1,11].

### 2.2. Medieval Medicine: Early Clinical Descriptions of Optic Nerve Inflammation

**Figure 2:**
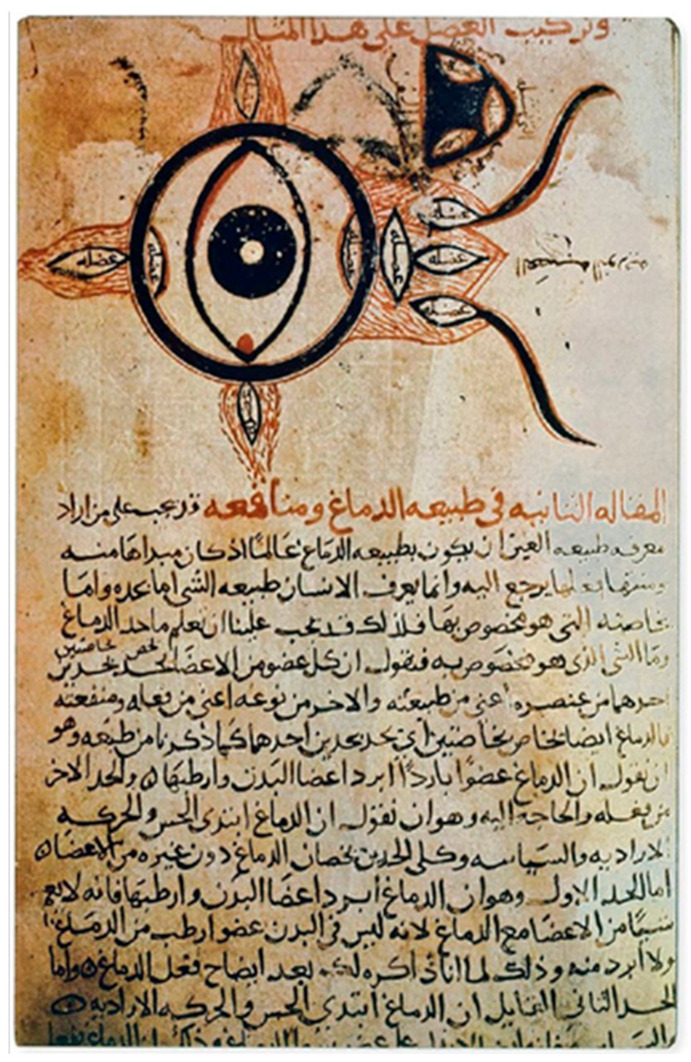
Hunayn ibn Ishaq 9th century CE description of the eye diagram in a copy of his book, Kitab al-Ashr Maqalat fil-Ayn (“Ten Treatises on the Eye”), in a 12th century CE edition. Source: Wikipedia [13].

A major conceptual advance occurred during the Islamic Golden Age. Hunayn ibn Ishaq (809–873 CE) provided the earliest detailed clinical descriptions consistent with ON in his *Kitab al-Ashr Maqalat fil-Ayn* (*Ten Treatises on the Eye*). He described acute visual loss occurring without apparent ocular disease, often accompanied by pain and sometimes followed by recovery—features closely aligned with modern ON [12]. Figure 2 illustrates an eye and optic pathway diagram from a later copy of Hunayn’s work, reflecting the anatomical and conceptual framework underlying his observations.

The earliest known use of the expression “inflammation of the optic nerve” (*waram ʿaṣab al-baṣar*) appears in Ali ibn Isa al-Kahhal’s *Tadhkirat al-Kahhalin* (c. 1010 CE), which described painful visual loss attributed to optic nerve disease and its occasional association with systemic conditions [14].

Avicenna (Ibn Sina, 980–1037 CE) further advanced visual physiology by relocating the faculty of vision from the crystalline lens to the optic nerve in *The Canon of Medicine*, thereby reframing visual loss as a neurological disorder rather than a purely ocular one [15]. His therapeutic approaches to inflammatory eye disease implicitly acknowledged inflammation as a cause of visual impairment.

### 2.3. Renaissance to Early Modern Era: Anatomical Precision and Functional Correlation

The Renaissance marked the definitive abandonment of Galenic visual doctrine. Vesalius demonstrated that the optic nerves are solid neural structures, while Kepler established the retina as the site of image formation, enabling a clear distinction between retinal and optic nerve pathology. Thomas Willis later linked optic chiasm lesions to characteristic visual field defects, strengthening clinico-anatomical correlations. By the 18th century, Zinn’s detailed descriptions of the optic nerve and orbital anatomy completed the anatomical framework required for modern neuro-ophthalmology [16].

### 2.4. The Modern Era: Clinical Characterization Before the ONTT

The invention of the ophthalmoscope by Helmholtz (1851) enabled direct visualization of the optic disc, transforming the diagnosis of ON. Shortly thereafter, von Graefe described inflammatory optic disc changes and distinguished papillitis from papilledema, recognizing that ON could occur in isolation or in association with neurological diseases [17]. Nettleship’s 1884 description of retrobulbar neuritis established that acute ON may present with a normal fundus and identified key features such as a central scotoma, dyschromatopsia, and pain with eye movement [18].

Physiological observations—including Uhthoff’s phenomenon and the Pulfrich effect—provided functional correlates of demyelinating conduction failure [19]. Finally, Charcot integrated ON into the clinical spectrum of multiple sclerosis in 1868 [20]. Mid-20th-century epidemiological studies clarified the risk of MS following ON and stimulated early therapeutic trials with ACTH [21,22,23,24,25], directly paving the way for the Optic Neuritis Treatment Trial and the modern era of evidence-based management [26,27].

Despite these cumulative anatomical and clinical insights, ON remained a loosely defined clinical syndrome, lacking standardized diagnostic criteria, prognostic markers, or evidence-based therapeutic guidance. This uncertainty—particularly regarding visual outcomes and the risk of developing MS set the stage for the ONTT, which represented the first systematic effort to define and standardize a specific clinical phenotype of ON.

## 3. The Optic Neuritis Treatment Trial and Characterization of Multiple Sclerosis-Related Optic Neuritis

By the 1970s and 1980s, the management of acute ON remained largely empirical. Systemic corticosteroids were widely used, yet the evidence supporting their efficacy was inconsistent and derived mainly from small, uncontrolled studies. Questions persisted regarding the optimal dose, route, and duration of therapy, and uncertainty extended to the natural history of ON itself—particularly its long-term visual prognosis and the magnitude of its association with MS. Although epidemiologic observations suggested that ON frequently served as a presenting symptom of MS, robust prospective data quantifying this risk were not yet available. The advent of magnetic resonance imaging (MRI) in the mid-1980s provided an unprecedented means of detecting clinically silent demyelinating lesions, but its prognostic value in the setting of a first ON episode had not been systematically evaluated. This convergence of therapeutic ambiguity, prognostic uncertainty, and emerging neuroimaging technology created a compelling rationale for a rigorously designed, large-scale clinical trial.

To address these gaps, the Optic Neuritis Study Group (ONSG) was established as a multicenter collaborative consortium supported by the National Eye Institute. Bringing together leading neurologists and neuro-ophthalmologists from 15 clinical centers across the United States, the ONSG undertook the first comprehensive, prospective characterization of demyelinating ON and designed what would become the ONTT. The ONTT was conceived with two principal objectives. The first was to determine whether specific corticosteroid regimens—namely high-dose intravenous methylprednisolone followed by oral prednisone, oral prednisone alone, or placebo—could accelerate visual recovery or improve long-term visual outcomes in acute ON. The second objective was to define the long-term risk of MS following a first episode of ON and to evaluate whether baseline brain MRI could stratify that risk with clinical utility.

Over the next two decades, the ONTT and its extension studies generated an extensive body of work, including seminal reports on treatment effects, visual recovery trajectories, late visual function, quality of life, recurrence rates, and MS conversion risk. These publications collectively transformed the clinical understanding and management of ON [2,28,29,30,31,32,33,34,35,36,37,38,39,40,41,42,43,44,45,46,47,48,49,50,51].

Together, the ONTT and its related investigations established the evidence-based framework that continues to guide the diagnosis, treatment, and prognostication of MS-related ON.

### 3.1. Demographic and Clinical Characterization of Optic Neuritis in the ONTT

Between 1988 and 1991, the ONTT enrolled 457 patients with a first episode of acute unilateral ON across 15 clinical centers in the United States [26]. The cohort was predominantly Caucasian (85.3%), with smaller proportions of African American (12.7%), Asian (1.5%), and Hispanic (0.4%) participants. Eligibility required age between 18 and 45 years and the presence of a first episode of acute unilateral ON of ≤8 days’ duration, typically characterized by sudden visual loss accompanied by periocular pain. Clinical examination had to be consistent with ON, including an RAPD and either optic disc swelling or a normal disc in retrobulbar cases. Visual acuity (VA) in the affected eye was required to be worse than 20/40 but not no light perception (NLP), while that of the fellow eye had to be better than 20/40. Patients were excluded if they exhibited atypical clinical features—such as marked optic disc swelling with hemorrhages or exudates, progression beyond two weeks, or absence of pain—prior ON in the affected eye, or VA worse than 20/40 in the fellow eye. Additional exclusion criteria included systemic diseases capable of mimicking ON (e.g., sarcoidosis, syphilis, Lyme disease, systemic lupus erythematosus), other ocular or neurological conditions affecting vision, contraindications to corticosteroid therapy (such as uncontrolled hypertension, diabetes, active infection, psychosis, or peptic ulcer disease), pregnancy or lactation, or exposure to systemic corticosteroids within the previous 30 days.

The baseline demographic and clinical characteristics of the ONTT cohort are summarized in Table 1. These data outline the visual function profile and symptomatology of the 457 patients at study entry [26]. The table includes demographic variables, laterality, and the frequency of simultaneous bilateral involvement, as well as key baseline measures such as high-contrast visual acuity (HCVA), color vision, contrast sensitivity (CS), visual field findings, and the presence of an afferent pupillary defect. It also reports the prevalence of eye pain or headaches accompanying vision loss, a hallmark feature of acute demyelinating ON in this population.

### 3.2. Assessment of Corticosteroid Treatment

The selected ONTT participants were randomized to one of three groups: (1) IV methylprednisolone (IVMP) + oral prednisone: 250 mg IV every 6 h for 3 days (1 g/day), then oral prednisone 1 mg/kg/day for 11 days with taper; (2) oral prednisone alone (1 mg/kg/day for 14 days); or (3) oral placebo: 14 days. The study showed that IVMP significantly accelerated recovery of visual function, with earlier improvement in visual fields (*p* = 0.0001), CS (*p* = 0.026), and color vision (*p* = 0.033) compared to placebo, though no long-term VA benefit was observed. Oral prednisone alone was ineffective and increased recurrence risk [2].

At five-year follow-up most patients retained good vision (≥20/20 VA in ~74% of affected eyes). Baseline MRI lesions predicted a ~50% 5-year MS risk vs. ~25% with normal MRI [43,44]. At 10 years, 74% had ≥20/20 VA, 18% had 20/25–20/40, 5% had <20/40–20/200, and 3% had <20/200. Recurrence occurred in ~35%, more often in MS patients (*p* < 0.001). At 15–18 years, 72% of affected eyes and two-thirds of patients had ≥20/20 VA bilaterally. Multiple sclerosis remained associated with slightly worse outcomes and quality-of-life measures [47,52,53].

### 3.3. Multiple Sclerosis Risk and Prognostic Value of MRI

A pivotal contribution of the ONTT was the detailed characterization of the long-term risk of MS following the first episode of acute demyelinating ON while meeting the study’s inclusion criteria. These findings established the prognostic utility of baseline brain MRI as the most powerful predictor of MS conversion in isolated ON [28,43,47,49], thereby defining what is now understood as typical demyelinating ON.

In the final long-term ONTT follow-up, conducted 14–18 years after enrollment (mean, 15 years), the cumulative probability of clinically definite MS for the entire cohort was approximately 50% [49]. Baseline brain MRI performed at study entry stratified patients into two distinct prognostic groups. Individuals with one or more characteristic white-matter lesions—defined at the time as ovoid, ≥3 mm in diameter, and located in typical MS regions such as periventricular, juxtacortical, infratentorial areas, or the corpus callosum—had a 15-year MS conversion risk of approximately 72%. In contrast, patients with a normal MRI had a markedly lower conversion probability of approximately 25% [49].

The temporal pattern of MS conversion demonstrated a biphasic distribution. The majority of conversions occurred within the first five years, particularly among patients with MRI abnormalities [43]. Conversions continued at a slower rate thereafter, with very few new cases after ten years among patients with normal baseline MRI, suggesting that long-term risk eventually plateaus [49].

Several clinical factors increased MS risk independently of MRI findings. These included older age at ON onset, female sex, relapsing ON (RON), and the presence of additional neurological symptoms or abnormal neurological examination at presentation [54,55,56]. Conversely, severe visual loss and optic disc swelling—particularly in children—were associated with a lower likelihood of MS conversion, although these features were not strong independent predictors once MRI was taken into account [56].

Optical coherence tomography (OCT) has also emerged as a potential prognostic tool. Thinning of the ganglion cell internal plexiform layer (GCIPL) and the peripapillary retinal nerve fiber layer (pRNFL) in both the affected and the fellow eye may serve as independent predictors of MS conversion after ON [57,58].

Recurrent ON occurred more frequently among patients who subsequently developed MS, affecting approximately one-third of the ONTT cohort [26,49,52]. In many cases, involvement of the fellow eye occurred in close temporal proximity to the MS diagnosis. These observations firmly established MRI as an essential tool for long-term prognostication and directly influenced revisions to the McDonald diagnostic criteria in 2001, 2005, 2010, 2017, and 2024, which increasingly relied on MRI evidence of dissemination in space (DIS) and time (DIT) to confirm MS after a single demyelinating event [59,60,61,62,63].

### 3.4. The Role of Optic Neuritis in the Diagnosis of Multiple Sclerosis

The evolution of the McDonald criteria over the past two decades has progressively reshaped the diagnostic weight assigned to ON in MS, integrating increasingly sophisticated imaging and laboratory biomarkers into the diagnostic framework [59,60,61,62,63,64,65] (Table 2).

The 2001 and 2005 revisions relied primarily on conventional MRI evidence of DIS and DIT, permitting the diagnosis of MS after a first demyelinating episode—such as ON—when radiological thresholds were fulfilled [59,63]. The 2010 update advanced this framework by allowing DIS and DIT to be demonstrated on a single MRI scan through the simultaneous presence of enhancing and non-enhancing lesions, thereby accelerating MS diagnosis in typical ON presentations [60].

In 2017, cerebrospinal fluid (CSF)-specific oligoclonal bands (OCBs) were accepted as an alternative to demonstrating DIT, although the optic nerve itself still did not qualify as a DIS site [61]. This long-standing limitation was fundamentally revised in the 2024 McDonald update, which recognized the optic nerve as the fifth topographic region for DIS, allowing ON-related lesions—identified on orbital MRI, visual evoked potentials (VEP), or OCT—to directly support MS diagnosis [62,66].

This shift is particularly relevant for patients presenting with isolated ON and borderline MRI findings. In such cases, advanced imaging markers now incorporated into the criteria—such as the central vein sign (CVS) and paramagnetic rim lesions (PRLs)—provide additional specificity [67,68,69,70].

The CVS reflects the perivenular origin of demyelinating plaques in MS. It appears as a small central vessel traversing white-matter lesions on susceptibility-weighted or T2* imaging [71], corresponding to classic histopathological descriptions of MS lesions centered on small veins [72]. Quantitative thresholds—such as ≥40% of lesions showing a central vein—have demonstrated high diagnostic accuracy in distinguishing MS from ischemic, migraine-related, and other inflammatory white-matter disorders [73].

The PRLs also referred to as “iron rim lesions”, reflect chronic active demyelination in MS. They exhibit a demyelinated, hypocellular core surrounded by iron-laden activated microglia/macrophages, producing a paramagnetic rim on susceptibility-based MRI [74,75]. PRLs are highly specific for MS and rare in ischemic small-vessel disease or neuromyelitis optica spectrum disorder (NMOSD) [76]. Their presence correlates with accelerated brain atrophy, greater clinical disability, and worse long-term outcomes [77,78]. Longitudinal studies have demonstrated that PRLs are relatively specific for MS, distinguishing it from other white matter diseases such as small vessel ischemia or NMOSD, in which PRLs are rare [76]. The presence of PRLs has been associated with greater clinical disability, faster brain atrophy, and worse long-term outcomes, underscoring their prognostic value [69,76,77,78,79].

Optical coherence tomography has become an integral component of the 2024 McDonald criteria, reflecting its value as a structural biomarker in patients presenting with optic ON. By quantifying axonal and neuronal integrity in the retina, OCT provides objective evidence of optic nerve involvement that complements MRI and CSF analysis [66]. Both, the pRNFL and the GCIPL are particularly informative after ON. They exhibit characteristic thinning following demyelination, with GCIPL loss occurring earlier and correlating more consistently with functional outcomes such as low-contrast visual acuity (LCVA), color vision, and visual field sensitivity [80,81].

Intereye absolute difference (IEAD) substantially enhances diagnostic sensitivity for prior unilateral ON. Validated thresholds of ≥9 μm for pRNFL and ≥6 μm for GCIPL reliably distinguish affected from unaffected eyes, even when clinical history is uncertain or visual evoked potential (VEP) are inconclusive [82,83].

Importantly, OCT also detects subclinical retinal thinning in MS eyes without a history of ON, reflecting diffuse neuroaxonal injury that parallels global CNS atrophy [84]. This extends OCT’s utility beyond the assessment of ON and supports its use as a marker of neurodegeneration in MS more broadly.

Early OCT changes additionally hold prognostic value as the magnitude of GCIPL and pRNFL thinning in the weeks following ON predicts long-term visual function, and retinal atrophy may progress despite clinical recovery, indicating ongoing neurodegeneration [80].

Given its reproducibility, quantitative precision, and sensitivity to small structural changes, OCT is now routinely incorporated into multicenter clinical trials evaluating neuroprotective and remyelinating strategies [85]. Its integration into the 2025 diagnostic criteria reflects the growing emphasis on multimodal, objective biomarkers to strengthen the diagnostic framework in clinically isolated ON and early MS.

Kappa free light chains (KFLCs) have emerged as a highly sensitive, quantitative marker of intrathecal B-cell activity and are now incorporated into the 2025 McDonald criteria as an accepted alternative to CSF-specific OCBs. KFLCs are released in excess during immunoglobulin synthesis, and their measurement—typically expressed as the KFLC index—provides a reproducible and automated assessment of intrathecal immunoglobulin production [86].

Large multicenter studies have established the strong diagnostic performance of KFLCs. In an early, influential investigation, Presslauer et al. reported a diagnostic sensitivity of 95% for intrathecal KFLC synthesis in MS, compared with 93% for OCBs, with both biomarkers demonstrating 95% specificity [86]. These findings were subsequently supported by Leurs et al., who demonstrated a sensitivity of 88% (95% CI 85–90%) for the KFLC index versus 82% (95% CI 79–85%) for OCBs, with specificities of 83% and 92%, respectively [87].

Meta-analytic data further validate the robustness of KFLC. A systematic review encompassing 32 studies reported weighted mean sensitivities of 88% for the KFLC index and 85% for OCBs, with specificities of 89% and 92%, respectively [88]. Complementary findings from Nabizadeh et al. demonstrated pooled KFLC sensitivities of 90–91% and specificities of 86–87%, confirming its diagnostic accuracy across diverse populations [89].

In addition to its diagnostic power, KFLC offers several practical advantages over isoelectric focusing (IEF) for OCB detection, including automation, quantification, reduced inter-observer variability, faster turnaround time, and improved cost-effectiveness [87,90]. These operational strengths have led multiple consensus statements to recommend KFLC as a core biomarker for MS diagnosis [88,91].

The integration of KFLC into the 2024 McDonald criteria reflects a broader shift toward multimodal, quantitative, and reproducible biomarkers that enhance diagnostic certainty, reduce delays in identifying MS after a first demyelinating event, and improve differentiation from mimicking inflammatory or infectious conditions. When combined with MRI and OCT findings, KFLC substantially strengthens the diagnostic framework for patients presenting with ON and other clinically isolated syndromes.

## 4. Beyond the ONTT: Non-Multiple Sclerosis-Related Optic Neuritis (“Atypical Optic Neuritis”)

The ONTT (1992–2008) established the benchmark clinical phenotype of acute demyelinating ON and clarified the effects of corticosteroid therapy on visual outcomes [2,26,29,30,31,32,33,35,36,37,38,40,41,42,43,44,45,46,47,48,50,51,52,92,93]. It also defined the risk of conversion to MS over different follow-up periods [28,38,47,49]. Clinical features that diverge from the ONTT-defined profile—such as painless presentation, bilateral or rapidly sequential involvement, severe optic disc edema with hemorrhages or exudates, poor visual recovery, and associated systemic signs—were subsequently considered “atypical” and recognized as red flags warranting an expanded diagnostic evaluation [94].

The term “atypical optic neuritis” subsequently emerged as a practical umbrella designation for these phenotypes that deviate from the ONTT-defined profile and encompass a heterogeneous group of inflammatory optic neuropathies, including those associated with NMOSD, MOGAD, chronic relapsing inflammatory optic neuropathy (CRION), infectious etiologies, neurosarcoidosis, and neuroretinitis [94,95].

Table 3 shows the classification of ON with emphasis on the immune-mediated subtypes according to their etiopathogenic mechanisms, with representative references for each category [2,5,26,29,30,31,32,33,34,35,36,37,38,40,41,42,43,44,45,46,47,48,49,50,52,53,92,93,96,97,98,99,100,101,102,103,104,105,106,107,108,109,110,111,112,113,114,115,116,117,118,119,120,121,122,123,124,125,126].

The International Consensus Optic Neuritis (ICON) Criteria distinguish *Single Isolated Optic Neuritis (SION)*—a first episode of ON in individuals who do not fulfil diagnostic criteria for MS, NMOSD, or MOG-antibody disease—from *ON occurring in established MS*, which reflects disease activity within a recognized MS phenotype [5,127,128].

This conceptual separation builds on earlier classifications that defined idiopathic isolated ON as a discrete clinical construct within the spectrum of autoimmune optic neuropathies [128].

Despite this categorical distinction, SION and MS-associated ON share nearly identical clinical and paraclinical profiles, including acute unilateral painful visual loss, the presence of a relative afferent pupillary defect, short-segment retrobulbar enhancement on MRI, and characteristic OCT patterns of GCIPL and pRNFL thinning—features consistent with a shared demyelinating pathophysiology [127,129,130].

The 2024 McDonald diagnostic criteria further strengthen this biological continuum. Under the revised framework, MS may be diagnosed at the time of a first demyelinating event—such as SION—when dissemination in space is demonstrated and when supportive MRI or CSF biomarkers, including the central vein sign, paramagnetic rim lesions, or optic nerve involvement, are present. As a result, some cases previously classified as SION now meet diagnostic criteria for MS even in the absence of clinical dissemination in time, underscoring the diagnostic relevance of early radiological markers and the continuum between isolated ON and MS.

This distinction has been operationalized in prospective datasets, including the Acute Optic Neuritis Network (ACON), which stratify patients as SION or MS-ON based on McDonald diagnostic status at presentation [131,132]. Longitudinal studies consistently show that a substantial proportion of patients presenting with SION eventually fulfil criteria for MS, thereby transitioning from an isolated optic neuropathy to MS-associated ON. In the ONTT, approximately 50% of individuals with a first episode of typical ON converted to MS over 15 years, with baseline brain MRI abnormalities representing the strongest predictor of conversion [48]. Similar long-term trajectories have been demonstrated in European and Finnish cohorts evaluating idiopathic ON [133,134].

More recent analyses within the McDonald 2017 and 2024 frameworks confirm that ON frequently represents the first clinical manifestation of MS [135,136].

**Table 3:** Types of Optic Neuritis According to Etiopathogenetic Mechanisms.

Type			References
Infectious ON			[5,96,97,137]
Immune-mediated ON			
	MS-related ON (Typical ON)		[2,26,28,29,30,31,32,33,34,35,36,37,38,39,40,41,42,43,44,45,46,47,48,49,50,51,52,53,92,93]
	Non-MS-related ON (Atypical ON)		
		AQP4-Related ON	[98,99]
		MOG-Related-ON	[100,126]
		Paraneoplastic ON (CRMP5-ON)	[101,102]
		GFAP-Related ON	[103,104,105,106,107]
		Glycine Receptor-Related ON	[108,109,110]
		Post-infection-ON	[111,112]
		Post-vaccination ON	[114,115,116]
		Recurring Idiopathic ON RION	[117,119]
		CRION	[120,122]
		ON in systemic autoimmune disorders	[123,124,125]
		Other immune-mediated ON of undetermined etiopathogenesis	[6,123]

ON—optic neuritis; AQP4—aquaporin-4; MOG—myelin oligodendrocyte glycoprotein; CRMP5—collapsin response-mediator protein 5; GFAP—glial fibrillary acidic protein; RION—relapsing isolated optic neuritis; CRION—chronic relapsing inflammatory optic neuropathy.

Collectively, these data indicate that although SION and MS-associated ON are often treated as separate diagnostic categories, they likely represent two temporal expressions of the same underlying demyelinating disease process. Their differentiation primarily reflects the timing of detection of additional MS-typical lesions rather than fundamental distinctions in clinical, radiological, or pathological mechanisms. The 2024 McDonald criteria reinforce this interpretation by enabling MS diagnosis at the first demyelinating event when appropriate supportive biomarkers are present, integrating isolated ON more directly into the MS disease spectrum.

At the time of the ONTT, AQP4-IgG and MOG-IgG antibodies had not yet been discovered. In a recent reanalysis of stored serum from 177 ONTT participants, none tested positive for AQP4-IgG, while 3 patients (1.7%) were positive for MOG-IgG [138]. All MOG-IgG-positive patients presented with optic disc edema and had good recovery of VA, though one had persistent peripheral visual field loss. Two experienced a single episode of recurrent ON, but none developed MS or had demyelinating lesions on MRI during 15 years of follow-up. These results show that MOG-IgG and AQP4-IgG are rare in typical ONTT cases, and that MOG antibody-associated disease (MOGAD) is clinically and prognostically distinct from MS [138].

### 4.1. Aquaporin 4-Related Optic Neuritis

The identification of AQP4-IgG marked the first unequivocal demonstration that a substantial subset of ON represents a primary astrocytopathy rather than a demyelinating disease, fundamentally challenging the ONTT-derived assumption that ON is pathophysiologically homogeneous. AQP4-related ON is a severe, relapsing autoimmune disorder with distinct epidemiology, pathophysiology, clinical features, and prognosis. It is a hallmark of NMOSD and is associated with severe visual impairment and a high risk of permanent disability [139].

**Epidemiology**—The reported prevalence of AQP4-IgG seropositivity in patients with isolated ON varies widely according to age and geographic region, ranging from 4% in non-Asian adults to 27% in Asian adults. In pediatric ON, AQP4-IgG is rare, being detected in only 0.4% of non-Asian children but in up to 15% of Asian children [140].

Population-based data from Olmsted County show the antibody prevalence in 3% of ON cases [141].

Conversely, ON represents a frequent manifestation of NMOSD, accounting for 50–70% of first clinical presentations in seropositive patients [142].

The mean age of onset varies across populations, reported at 26.2 ± 11.0 years in a Turkish cohort and 38.6 ± 13.7 years in a Chinese cohort [143,144].

A striking female predominance is observed, with female-to-male ratios ranging from 6.5:1 to 14.7:1 in AQP4-IgG-positive NMOSD cohorts [145]. This sex imbalance is particularly pronounced during reproductive age, when the ratio can reach 23:1 [146].

Epidemiological studies consistently show that Asian, Black, and Latin American patients—both adults and children—are more frequently affected than Caucasians [147,148]. These groups also face a higher risk of developing NMOSD and tend to have worse clinical outcomes [149,150,151].

**Pathophysiology**—In AQP4-ON antibodies of the IgG1 subclass target AQP4 water channel densely expressed at astrocytic endfeet in the optic nerves. Binding of AQP4-IgG triggers complement-dependent cytotoxicity, perivascular deposition of C5b-9, astrocyte injury/necrosis, and secondary oligodendrocyte and axonal loss [98,152,153,154].

Histopathology shows loss of AQP4 and glial fibrillary acidic protein (GFAP) along with perivascular IgG/complement and granulocyte infiltration. These findings indicate a primary astrocytopathy rather than a primary demyelinating process [154]. The optic nerve’s high AQP4 density and relative paucity of complement regulators likely contribute to its particular vulnerability in AQP4-ON [155].

Experimental models of ON induced by passive transfer of AQP4-IgG have successfully reproduced the histological features observed in NMOSD, including severe visual dysfunction [156].

Recent transcriptomic analyses suggest that inflammation in AQP4-ON is mediated by damage-associated molecular patterns (DAMPs) and involves selective activation of toll-like receptors (TLR2, TLR5, TLR8, TLR10), with immune cell infiltration correlating with visual impairment [157].

Additionally, gene expression analyses have identified histone modification genes as potential biomarkers, indicating a role for epigenetic regulation in disease pathogenesis [158].

**Clinical features**—AQP4-ON is consistently associated with profound vision loss at nadir, often reaching 20/200 or worse. In a large Japanese cohort, the median nadir VA in AQP4-ON was 20/2000, and even after treatment, the median final VA improved only to 20/50, which is significantly worse than outcomes in MOG-ON or MS-ON.

In a population-based US study, most AQP4-ON patients had multiple attacks, and two-thirds were left with NLP in at least one eye [141].

Similarly, a large Chinese cohort found that 42.9% of AQP4-ON eyes remained ≤20/200 at final follow-up, while only 42.9% achieved ≥20/40, highlighting the high risk of permanent legal blindness [159].

Chiasmal involvement is a notable feature in AQP4-ON, occurring in approximately 20% of cases and demonstrating microstructural damage that correlates with reduced VA and pRNFL thinning [155], a prevalence similar to that seen in MOG-IgG-ON but with distinct patterns—AQP4-ON more often affects the posterior chiasm, whereas MOG-IgG-ON typically shows LEON lesions extending from the orbit to the chiasm [160].

**Treatment and outcome**—Acute attacks of AQP4-ON are primarily managed with high-dose IVMP, which should be administered ideally within three days of onset to optimize visual recovery as even a short delay can significantly worsen prognosis [161,162,163]. In cases where response to steroids is inadequate, plasma exchange (PLEX) is recommended and has been shown to improve visual outcomes, especially when initiated early [164].

Long-term relapse prevention relies on immunosuppressive therapies, including rituximab, azathioprine, and mycophenolate mofetil, with recent evidence supporting the preferential use of monoclonal antibodies such as ravalizumab, inebilizumab, and satralizumab [165,166,167].

Visual acuity at nadir is the strongest predictor of long-term outcome across all ON subtypes, including AQP4-ON. In patients with NMOSD, AQP4-ON at disease presentation is a predictor of poorer outcome than when it occurs in the course of the disease [168].

Maintenance immunosuppressive therapy also reduces recurrences and improves final VA, with patients on maintenance therapy achieving median VA of 20/20 at one year, compared to 20/200 in those without. Older age at onset and more recurrences are additional risk factors for poor outcome [159,161,169].

These distinctions are clinically decisive because early recognition of AQP4-ON mandates urgent escalation from corticosteroids to PLEX when response is incomplete and necessitates prompt initiation of long-term relapse-prevention therapy. Failure to correctly identify this subtype risks irreversible astrocytic and axonal injury, repeated attacks, and inappropriate exposure to MS disease-modifying therapies that are ineffective or potentially harmful in NMOSD.

### 4.2. MOG-Related Optic Neuritis

Myelin oligodendrocyte glycoprotein antibody-associated disease (MOGAD) is recognized as a distinct demyelinating disorder of the CNS [170,171] in which ON is the presenting symptom in 70–77% cases, especially in adults and late-adult onset patients [172,173].

**Epidemiology**—MOGAD prevalence ranges from 1.3–2.5 per 100,000 inhabitants, while its annual incidence is approximately 3.4–4.8 per million, with 20–40% of patients presenting a history of preceding infection or vaccination [174].

The frequency of MOG-ON among all cases of ON as the first sign of demyelinating diseases of the CNS varies by population and age group. Some pediatric studies have found that 18–27% of children with acquired demyelinating syndromes and ON were MOG-IgG positive [175,176]. The proportion of MOG-ON among isolated ON cases ranges from 25% to nearly 50% in pediatric cohorts [140,175,177].

However, in adult populations, the proportion is lower. In the US and Europe MOG-IgG-MOG-ON represents about 5% of all adult ON cases, while meta- analyses and systematic reviews show that the frequency in some Asian populations can be as high as 8–20% of all adult ON patients [141,178,179,180].

MOG-ON affects a wide age range, with a median or mean age at onset typically in the 20s to 40s, but cases have been reported from early childhood to late adulthood [176,181,182,183].

In MOGAD there is no strong female or male predominance: Most large studies report a female-to-male ratio close to 1:1 or slightly higher for females (1.2:1) [174]. However, an US pediatric cohort found 57% female [184] and a Quebec cohort found an equal sex ratio [185]. In Olmsted County (USA) and Martinique, 38% of MOGAD cases were female, suggesting some variability by region [186]. This is highly distinct from AQP4+ NMOSD, which has a strong female predominance [145].

Studies with European, Asian and North American cohorts show that there is no clear racial preponderance globally as MOGAD has been observed across all racial groups without a strong bias [174,186]. However, some differences in distinct ancestries have been observed. A study in Singapore found a slightly higher prevalence among Indians (2.48/100,000) compared to Malays (1.47/100,000) and Chinese (1.03/100,000) [187]. Another study in Olmsted County and Martinique found prevalence of 3.70/100,000 in Olmsted County and 2.61/100,000 in Martinique, with children, respectively, representing 29% and 11% of the MOGAD cohorts [186].

**Pathophysiology**—MOGAD represents a unique autoimmune demyelination driven by perivenous, antibody-mediated myelin injury. The disease is associated with pathogenic serum MOG-IgG, which targets the outermost surface of myelin sheaths, making it susceptible to autoimmune attack. Activated peripheral CD4^+^ T cells and MOG-specific B cells breach the blood–brain barrier, producing antibodies that induce complement activation and antibody-dependent cytotoxicity. Lesions show perivenous demyelination with macrophages, granulocytes, and CD4^+^ T-cell predominance, but relative astrocyte preservation—distinct from AQP4-NMOSD and MS. Pathological studies reveal that MOGAD lesions display both perivenous and confluent white matter demyelination, with a notable over-representation of intracortical demyelinated lesions compared to MS [188].

**Clinical features**—MOG-ON is often associated with severe visual loss at onset, with nadir VA frequently ≤20/200 in 50–80% of cases. Most patients experience substantial recovery, with median post-treatment VA improving to 20/20–20/45 [189,190]. In a large cohort study, only 6% of the MOG-ON patients had a final VA of 20/200 or worse [183]. In children recovery is even better; 56–85% achieve complete recovery, and 89–98% reach at least 20/40 [191,192,193]. Older age, female sex, and longer optic nerve lesion length predict worse final VA [194,195]. Ocular pain, especially with eye movement, is reported in 82–90% [196].

Optic disc swelling is a hallmark of MOG-ON, present in 53–100% of cases, and is more common than in AQP4-IgG or MS-associated ON [189,197]. Bilateral involvement is frequent, occurring in 50–84% of cases [198].

Relapsing ON (RON) is common: 47–80% of patients experience relapses, with persistent MOG-IgG seropositivity increasing this risk [163,190]. The annualized relapse rate (ARR) in relapsing cohorts ranges from 0.69 to 1.2 attacks per year [196,199]. The most common interval for the first relapse is within the first year, with a median time to first relapse of 5–6 months and 75–80% of relapsing patients experiencing their first relapse within 12 months [138,189,198]. The median number of relapses in relapsing patients is 2 (IQR 1–4), with over half experiencing two or more relapses [198,200].

Diagnosis of MOGAD requires a compatible clinical syndrome (such as ON, myelitis, or ADEM) detection of MOG-IgG in serum using a cell-based assay; exclusion of alternative diagnoses, especially MS and AQP4-IgG-positive NMOSD; and supportive MRI or neurophysiological evidence of demyelination [198].

**Treatment**—Acute attacks of MOG-ON are primarily managed with high-dose IVMP, which leads to favorable outcomes in most patients, especially when administered promptly; for example, 91% of patients achieved full visual recovery at three months, whereas delayed treatment (>10 days) significantly reduced the likelihood of optimal recovery [201,202].

If response to IVMP is insufficient after 3–5 days, escalation to intravenous immunoglobulin (IVIG), 1–2 g/kg over 1–5 days) or PLEX is recommended as second-line therapies [203]. IVIG has demonstrated significant improvement in disability and visual outcomes in acute attacks, with retrospective studies showing marked improvement in Expanded Disability Status Scale (EDSS) and VA (*p* < 0.0001) 2. PLEX is also effective, particularly in severe or steroid-resistant cases, and is commonly used after IVMP failure [202,203].

For maintenance therapy to prevent relapses, immunosuppressive agents such as rituximab, azathioprine, mycophenolate mofetil, and monthly IVIG are commonly used [202,203,204,205,206,207].

Observational studies indicate that IVIG provides the lowest annualized relapse rates (ARR: 0–0.13) and highest relapse-free probability (up to 72%), outperforming rituximab (ARR: 0.51), mycophenolate mofetil (ARR: 0.32), and azathioprine (ARR: 0.2) [204,205,206].

In adults, maintenance IVIG at 1 g/kg every 4 weeks is associated with a significant reduction in relapses, with only 17% relapsing at this dose and frequency [206].

Traditional MS disease-modifying therapies are generally ineffective in MOGAD [204,207].

The comparison of MOG-ON with AQP4-ON and MS-ON shows that similar clinical presentations can arise from different underlying immune mechanisms, each associated with distinct outcomes and treatment considerations. This understanding has shifted ON from a symptom-based clinical label to a biologically defined spectrum of diseases.

### 4.3. Distinct Clinical, Biomarker, and Imaging Profiles of MS-ON, AQP4-ON and MOG-ON

Patients with MS-ON, AQP4-ON, and MOG-ON exhibit distinct clinical, biomarker, MRI, and OCT profiles. Clinically, AQP4-ON and MOG-ON are more often bilateral and severe, with AQP4-ON showing the worst visual outcomes. MRI reveals longer, more posterior lesions in AQP4-ON and anterior, optic nerve head swelling in MOG-ON, while MS-ON lesions are typically unilateral and anterior. OCT demonstrates greater GCIPL and p-RNFL thinning in AQP4-ON and MOG-ON than MS-ON, correlating with visual impairment severity. Table 4 summarizes their main distinctive feature.

Despite these recent advances, a substantial proportion of patients with recurrent or severe ON remain seronegative for known antibodies, highlighting a residual diagnostic blind spot. These cases challenge current classification systems and suggest the existence of additional, yet-unidentified immune mechanisms. The current classification of double-seronegative ON (DN-ON) is therefore best regarded as a diagnostic residual category rather than a defined disease entity. Its definition relies primarily on exclusion—namely the absence of MS, AQP4-IgG, and MOG-IgG—rather than on positive biological markers. As a result, this category likely aggregates multiple distinct pathogenic processes that cannot yet be reliably distinguished using existing clinical, imaging, or laboratory criteria.

**Comparative diagnostic value, limitations, and accessibility of MRI and OCT in ON**—Although MRI and OCT are often presented as complementary diagnostic tools in ON, they differ substantially in their diagnostic roles, limitations, and real-world accessibility. MRI remains the cornerstone for etiological classification, providing critical information on lesion location, length, enhancement patterns, and associated CNS involvement. In MS-ON, MRI enables risk stratification for MS, while in AQP4-ON and MOG-ON it reveals characteristic features such as LEON lesions, posterior or chiasmal involvement, and perineural enhancement. However, MRI availability is uneven globally, acquisition protocols vary, and sensitivity for isolated optic nerve involvement may be limited in the acute phase, particularly in resource-constrained settings.

In contrast, OCT offers rapid, non-invasive, and highly reproducible quantification of retinal neuroaxonal injury. Measurements of the GCIPL and pRNFL provide objective markers of structural damage that correlate with visual function and long-term outcome across ON subtypes. OCT is particularly valuable for distinguishing patterns of injury—such as severe early GCIPL loss in AQP4-ON or relative structural preservation despite severe visual loss in MOG-ON—but lacks etiological specificity and cannot replace MRI for lesion localization or CNS assessment.

From a practical perspective, OCT may be more accessible, is less costly, and easier to standardize across centers than MRI, making it especially valuable in low- and middle-income regions. Conversely, MRI provides indispensable pathophysiological and prognostic information but is limited by cost, infrastructure requirements, and variability in access. Optimal diagnostic evaluation of optic neuritis therefore relies not on either modality alone, but on their integrated use, with MRI guiding etiological diagnosis and OCT refining prognosis, monitoring disease evolution, and quantifying cumulative injury.

### 4.4. Relapsing Optic Neuritis

Relapsing ON represents a heterogeneous group of ON with diverse etiopathogenesis and outcomes. A study of 246 patients at Mayo Clinic with at least two consecutive demyelinating ON attacks at presentation with or without a subsequent other demyelinating involvement, showed that about one third of these cases were related to AQP4-IgG or MOG-IgG, about 10% were related to MS, and 6% could be classified as CRION. Double negative isolated RON comprised 41% of the entire cohort [200].

That subtype of ON characterized by relapses restricted to the optic nerves without evidence of MS-, AQP4- or MOG-mediated disease has been classified as relapsing isolated optic neuritis (RION) [5].

Relapsing isolated optic neuritis affects both adults and children, with a slight female predominance in most cohorts. In a recent US cohort [223] the median age at onset was in the mid-30s, pediatric cases accounting for about 15% of the cohort, and over a quarter of these presented with bilateral involvement. No clear preceding infections were identified, and recurrences typically occurred within two months of the initial episode. Ocular or periocular pain is reported in 85–95% of cases, more frequently precedes vision loss by on to two days and is usually less severe than in NMOSD. Visual acuity at onset is generally less severe than in antibody-positive ON. Only 23% of patients presented with VA <20/200, compared to much higher rates in AQP4-ON and MOG-ON. Pediatric cases had a higher rate of severe vision loss at presentation. Visual recovery is typically favorable, and at one month follow-up, nearly 90% of patients achieved a VA of 20/40 or better; this proportion exceeding 95% at the last follow-up. In pediatric cases, all achieved a final VA of 20/40 or better. These outcomes are notably better than those seen in AQP4-ON and are similar to or slightly better than MOG-ON [223].

Optical coherence tomography provides a sensitive marker of cumulative damage following ON attacks. The average retinal nerve fiber layer (RNFL) loss following the first episode is about 20 μm within 3–6 months, and repeated attacks result in progressive thinning, particularly in the temporal quadrant. Ganglion cell–inner plexiform layer (GCIPL) loss parallels visual outcome. Nevertheless, compared with MOGAD or AQP4-IgG ON, RNFL and GCIPL loss in idiopathic RION tend to be milder [123].

### 4.5. Chronic Relapsing Inflammatory Optic Neuropathy

Chronic relapsing inflammatory optic neuropathy is a rare form of ON, characterized by recurrent, painful ON responsive to corticosteroids but prone to relapse on steroid withdrawal [120]. Unlike demyelinating MS-ON or AQP4-ON, CRION typically presents with normal brain MRI, absence of AQP4-IgG seropositivity, and dependency on long-term immunosuppression for relapse prevention. The hallmark of CRION is relapse upon steroid taper or discontinuation, typically within weeks to months. The median number of relapses across series ranges from 3 to 6, with an inter-relapse interval of about 4–6 months [121]. Some patients experience prolonged remission under maintenance immunotherapy, whereas others relapse repeatedly over years, accumulating optic atrophy and visual disability.

In the study of 122 cases, the median age at onset was 36 years (range 11–70), with a slight female predominance (~58%). Approximately 70% of cases were bilateral, often sequentially affected, and the majority occurred in adults without prior systemic autoimmune disease. Ethnic distribution indicated higher frequency among Caucasians and Asians, but later cohorts identified cases globally, including in Latin America and the Middle East [121,224].

At nadir, vision is often profoundly reduced—count fingers (CF), hand motion (HM), or NLP in up to 60% of eyes [120]. The weighted mean baseline VA across 122 cases was 20/160. Following IVMP, most eyes recovered to ≥20/40 (0.5 decimal) within weeks, but long-term follow-up revealed residual deficits in up to 40% of eyes. The mean final VA across studies was 20/33, though with marked interindividual variability. Patients with MOG-IgG–positive CRION exhibit better short-term recovery than AQP4-ON, yet relapse frequency leads to cumulative axonal loss and poorer OCT outcomes [122].

Recognition of CRION is critical due to its relapsing nature and potential for irreversible visual loss if untreated. MRI of the orbits shows enhancement of affected optic nerves in most cases, while brain MRI is usually normal, further distinguishing CRION from MS or NMOSD. Cerebrospinal fluid findings are usually unremarkable, or show mild pleocytosis without OCB, differing from MS. Optical coherence tomography studies reveal substantial p-RNFL thinning and GCIPL loss after recurrent attacks, supporting cumulative axonal damage even in steroid-responsive cases. Table 5 shows the revised diagnostic criteria for CRION.

A key discriminator of CRION is its steroid dependence and absence of systemic findings. Serological testing for AQP4- and MOG-IgG is mandatory for the diagnosis of any relapsing or bilateral optic neuritis.

The precise pathogenesis of CRION remains uncertain. Steroid responsiveness and dependency reflect a persistent inflammatory drive modulated by adaptive immunity. With the discovery of MOG-IgG, CRION was reinterpreted as part of the spectrum of MOGAD [122]. In the Seoul National University cohort, 11 of 12 patients meeting CRION criteria were MOG-IgG positive [122]. Similarly, other studies reported MOG-IgG in 50–90% of CRION-like cases, suggesting CRION is often a clinical phenotype within MOGAD rather than a distinct nosological entity [123]. MOG-IgG-associated CRION tends to manifest with younger onset (median 30–40 years), bilateral sequential attacks, prominent optic disc swelling (seen in up to 80%), excellent corticosteroid responsiveness but early relapse if tapered rapidly [122].

Visual outcome depends on early recognition and consistent immunotherapy. In the Petzold review, 36% of patients achieved complete recovery (≥20/25), 45% partial recovery (20/40–20/200), and 19% severe permanent loss (<20/200) [121]. Poor prognostic indicators include delayed treatment, high relapse frequency, and optic disc pallor. MOG-IgG–positive CRION has a relatively favorable prognosis, though with risk of cumulative structural damage positive [122].

### 4.6. GFAP-Related Optic Neuritis

Glial fibrillary acidic protein (GFAP) is an intermediate filament protein expressed by astrocytes and Müller cells that provides cytoskeletal stability and regulates astrocyte–neuronal signaling. GFAP astrocytopathy (GFAP-A) is a distinctive meningoencephalomyelitis characterized by CSF GFAP-IgG antibodies and a monophasic, steroid-responsive course [226].

GFAP-associated-ON (GFAP-ON) is rare, occurring in 6% of all GFAP-A cases. The mechanism of visual involvement in the disease is thought to be related to venous inflammation and perivascular processes, rather than direct demyelination or perineural inflammation typical of other ON etiologies [103]. Recent systematic reviews and clinical series [227,228,229,230,231] have established the clinical and imaging characteristics of the disease. It affects a wide age spectrum (median age 46 years), shows a slight male predominance, and has a worldwide distribution with no ethnic predilection.

When present, visual symptoms often accompany or follow systemic GFAP-A manifestations—headache, fever, meningismus, encephalopathy, or myelitis—reflecting widespread CNS inflammation. Typically, it presents with subacute bilateral, painless visual blurring and marked optic disc edema, occasionally with vitreous cells, mimicking papilledema. It should be suspected in bilateral disc edema without raised intracranial pressure or when accompanied by meningoencephalitis signs [123]. Visual acuity ranges from mild impairment to profound loss. At nadir it is usually 20/40–20/200, but patients recover to near-normal levels in most cases after corticosteroid therapy; mild optic atrophy persisted in 20%. Relapses occur in about 15–20% of patients, particularly in association with coexisting AQP4-IgG or neoplasia. In a pooled review, >80% of patients experienced favorable visual and neurological recovery after corticosteroids, confirming excellent reversibility [232].

The hallmark MRI feature of GFAP-A is linear, radial perivascular enhancement radiating from the ventricles into the deep white matter, best seen on post-contrast T1-weighted images. This pattern is highly suggestive of the disease and reflects perivenular inflammation. Patchy confluent hyperintense lesions in the periventricular, centrum semiovale, deep brain structures, brainstem, and cerebellum can be found. Spinal MRI may reveal extensive lesions, sometimes with punctate or patchy enhancement. Orbital MRI may show enhancement of the optic nerves, but this is not a consistent finding. Most visual involvement is due to bilateral optic disc edema without classic optic nerve enhancement; more often, optic disc edema occurs without significant MRI abnormalities of the optic nerve [103,233,234,235]. The CSF profile shows lymphocytic pleocytosis (70–90%), elevated protein (0.8–1.5 g/L), and frequent GFAP-IgG detection by cell-based assay. Rarely, elevated opening pressure is observed [232].

For GFAP astrocytopathy, treatment recommendations remain less well defined and may rely on extrapolation from similar autoimmune CNS disorders. High-dose IV corticosteroids for 5 days induce rapid improvement in >80% of cases [236]. For relapsing prevention or severe cases, mycophenolate mofetil, azathioprine, or rituximab are used as maintenance therapy.

Plasma exchange or IVIG is considered for steroid-refractory disease. Overall prognosis is favorable, though mild visual field defects or optic pallor may persist. Favorable prognosis correlates with early steroid therapy, absence of co-existing antibodies, and monophasic presentation. Poor outcomes relate to paraneoplastic GFAP-A, delayed treatment, or extensive myelitis [237].

### 4.7. CRMP5-Related Optic Neuritis

Collapsin response-mediator protein 5 antibodies are a marker of paraneoplastic autoimmunity, most frequently found in patients with small cell lung cancer and thymoma. It is associated with a broad spectrum of neurologic abnormalities, but painful polyradiculoneuropathy, ataxia, myelopathy, optic neuropathy, and cranial neuropathies are the most characteristic [238,239]. CRMP5-IgG has been identified in patients with paraneoplastic ON, vitritis, retinitis, or a combination thereof. Its frequency among all ON cases is very low, and is estimated to be lower than 1% of all ON cases [5,123,237]. In a series of 76 CRMP5-IgG-positive patients, 29 (38%) had neuro-ophthalmic manifestations (central nystagmus and diplopia), and only 18% ON [131]. Another study found that ON and/or retinitis occurred in 11% of patients with CRMP5 autoimmunity [238].

The pathology is characterized by microvasculitis affecting small venules and capillaries. CRMP5-ON most commonly affects older adults, with a median age of 67 years (range 33–88), and shows a female predominance (about 69%) (about 69%) [102]. At onset, median VA is moderately reduced (20/50, range 20/20 to CF), and the final median VA is similar or slightly improved (20/40, range 20/20 to hand movements) [102]. In all cases there is optic disc edema which is frequently associated with retinitis, vitritis, and uveitis in the patients [102]. Ocular motility dysfunction, such as central nystagmus and diplopia, occurs in about 41% of cases, and MRI typically does not show optic nerve enhancement [102]. Visual outcomes are variable: about half of patients receiving immunosuppressive therapy experience improvement, but overall, recovery is less favorable than in typical ON, and the prognosis is closely linked to the underlying malignancy [102,123].

Management of CRMP5-ON generally follows treatment protocols for atypical ON. Acute attacks are typically treated with high-dose intravenous corticosteroids as first-line therapy, often followed by an oral taper. In severe or refractory cases, PLEX may be considered to control the acute episode. Identification and treatment of underlying malignancy is also crucial for optimal outcomes. Long-term immunosuppressive therapy may be necessary to prevent relapses in some cases, and treatment should be tailored to the individual based on the underlying cause and associated symptoms [123].

### 4.8. Optic Neuritis in Systemic Autoimmune Diseases

Optic neuritis may occur as a neuro-ophthalmic manifestation of several systemic immune-mediated diseases [5]. Although uncommon in most of these conditions, its occurrence is clinically consequential, frequently reflecting active systemic inflammation and often requiring urgent immunosuppressive therapy to reduce the risk of irreversible visual loss. Compared with typical MS-associated ON, systemic autoimmune-associated ON more often presents with severe visual impairment at nadir, bilateral or LEON involvement, optic nerve sheath or chiasmal enhancement, and variable responsiveness to corticosteroid monotherapy. Table 6 summarizes the principal systemic diseases associated with ON, categorized by the consistency and strength of available evidence.

In accordance with the ICON 2022 framework, ON in systemic autoimmune diseases should be regarded as a distinct etiological category of immune-mediated ON, requiring systematic exclusion of MS, NMOSD, and MOGAD. In several conditions, ON may precede the diagnosis of systemic disease, underscoring the importance of integrating neuro-ophthalmic phenotype, MRI pattern, serological testing, and systemic evaluation.

**Sarcoidosis-associated optic neuritis and optic perineuritis.**

Sarcoidosis is a multisystem granulomatous disease of unknown etiology, with prevalence ranging from approximately 1 to 40 per 100,000 depending on ethnicity and geographic region [257]. Neurological involvement occurs in 5–26% of patients with systemic sarcoidosis, and up to one third of patients with neurosarcoidosis lack clinically apparent systemic disease at presentation [258].

Optic nerve involvement accounts for approximately 1–5% of sarcoidosis cases [259] and represents a characteristic neuro-ophthalmic manifestation of neurosarcoidosis. Pathophysiological mechanisms include granulomatous infiltration of the optic nerve, inflammation of the optic nerve sheath (optic perineuritis, OPN), contiguous spread from leptomeningeal disease, cavernous sinus involvement, or secondary effects of raised intracranial pressure. Although a large Japanese cohort of noninfectious ON reported no sarcoidosis-related cases [164], sarcoidosis remains one of the most consistently documented systemic causes of ON in Western cohorts.

Sarcoidosis-associated ON typically presents between 35 and 55 years of age, with a modest female predominance and increased frequency among Black and Northern European populations [257]. Onset is usually subacute and often painless. Initial unilateral involvement is common, but bilateral or sequential ON occurs in approximately 25–30% of cases [257]. Optic disc edema is frequent, and VA at nadir is often poor (20/200 to LP) particularly in cases with extensive perineural or chiasmal involvement [260].

Visual outcomes are variable. In a review of 52 cases, mean VA at presentation was approximately 20/400, with improvement to ≥20/60 in 56% following treatment [260]. Poor prognostic factors include bilateral disease, chiasmal extension, delayed therapy, and chronic granulomatous infiltration leading to secondary optic atrophy.

Optic perineuritis is a distinctive phenotype recognized within the ICON classification as a specific inflammatory optic neuropathy characterized by primary sheath involvement. Clinically, sarcoid-related OPN presents with progressive visual loss, frequent orbital pain, and circumferential optic nerve sheath enhancement on MRI, often sparing the nerve core. OPN is typically corticosteroid-responsive but prone to relapse with rapid tapering [261].

Magnetic resonance imaging findings include fusiform optic nerve enlargement, enhancement extending to the optic chiasm, and characteristic “tram-track” or “doughnut” enhancement patterns on post-contrast fat-suppressed T1-weighted images. Leptomeningeal enhancement, particularly at the skull base, occurs in up to 40% of cases [260].

Cerebospinl fluid abnormalities occur in approximately 50–70% of patients and include lymphocytic pleocytosis and elevated protein; oligoclonal bands may be detected but are typically nonspecific. Serum ACE is elevated in about 60% of cases, while CSF ACE or soluble interleukin-2 receptor levels may support the diagnosis but lack sensitivity [257,261].

The differential diagnosis includes infectious meningitides, IgG4-related disease, lymphoma, and vasculitis [261]. In line with consensus recommendations, histological confirmation from accessible non-neural tissue is strongly encouraged to establish the diagnosis and exclude mimics [260]. Diagnostic stratification follows the *Neurosarcoidosis Consortium and International Workshop on Ocular Sarcoidosis* (IWOS) criteria, summarized in Table 7, with non-caseating granulomas on tissue biopsy remaining the diagnostic gold standard [260,262].

High-dose systemic corticosteroids constitute first-line therapy and often result in rapid improvement in pain and partial visual recovery. Relapsing or corticosteroid-dependent disease frequently requires steroid-sparing immunosuppression or biologic therapy. Visual prognosis depends on disease burden, timeliness of treatment, and extent of optic nerve involvement.

**Systemic lupus erythematosus-associated optic neuritis.**

Optic neuritis occurs in approximately 0.6–1% of patients with systemic lupus erythematosus (SLE) and may represent the initial manifestation of the disease [263]. Pathogenic mechanisms include immune-mediated vasculitis, microthrombotic injury—particularly in the presence of antiphospholipid antibodies—and direct inflammatory damage to the optic nerve [241,263].

Systemic lupus erythematosus-associated ON is typically severe at onset, with most patients presenting with VA worse than 20/200 [241]. Visual outcomes are heterogeneous, ranging from meaningful recovery with early aggressive immunosuppression to permanent visual impairment despite therapy [264]. More than one third of affected eyes remain legally blind across published series [241,263].

Diagnosis requires optic neuropathy in conjunction with established clinical or serological criteria for SLE. CSF findings are usually unremarkable, and the absence of persistent oligoclonal bands helps distinguish SLE-associated ON from MS-associated ON. Testing for AQP4-IgG and MOG-IgG is mandatory to exclude NMOSD or MOGAD overlap. MRI frequently demonstrates longitudinally extensive optic nerve enhancement, with occasional chiasmal involvement [265].

High-dose intravenous corticosteroids are the recommended initial therapy. In corticosteroid-refractory cases, intravenous cyclophosphamide pulse therapy has demonstrated benefit, while PLEX may be considered in severe or resistant cases [220,266]. Owing to the absence of randomized controlled trials, management is guided by observational data and expert consensus [220,221,266].

**Sjögren’s syndrome-associated optic neuritis.**

Optic neuritis is an uncommon manifestation among patients with Sjögren’s syndrome (SS), yet SS is overrepresented among ON cohorts with autoimmune features. In a Chinese series, 7.9% of ON patients fulfilled diagnostic criteria for SS, particularly among those with bilateral or recurrent ON [267]. When ON occurs in SS, it frequently reflects underlying NMOSD, especially in AQP4-IgG–positive patients [268,269].

Sjogren’s syndrome-associated ON is often severe at onset, with VA commonly worse than 20/800 or counting fingers [225,270]. Early corticosteroid treatment may result in meaningful improvement, whereas delayed therapy or recurrent attacks are associated with incomplete recovery and cumulative optic nerve damage [225]. Female predominance and relapsing disease course are characteristic [227,268].

Diagnosis requires standard SS evaluation alongside neuro-ophthalmic assessment. In accordance with ICON recommendations, AQP4-IgG testing is essential, as antibody positivity reclassifies the disorder as NMOSD and directly informs prognosis and long-term management [227,268].

Acute treatment consists of high-dose intravenous corticosteroids, often followed by maintenance immunosuppression to reduce relapse risk. Plasma exchange is reserved for steroid-refractory attacks. Long-term immunosuppressive therapy is strongly recommended for relapsing diseases, particularly in AQP4-IgG-positive patients [227].

### 4.9. Post-Infectious and Post-Vaccination Optic Neuritis

Post-infectious ON is an immune-mediated inflammatory demyelinating optic neuropathy that develops after recovery from a systemic infection rather than from direct microbial invasion of the optic nerve. It represents a frequent cause of bilateral ON in children and young adults and typically follows a viral, or less commonly bacterial, illness by several weeks. Reported antecedent infections include the varicella-zoster virus, the herpes simplex virus, measles, mumps, influenza, and the Epstein–Barr virus [228,229,230], with more recent recognition of *Mycoplasma pneumoniae*, arboviral infections such as dengue, and SARS-CoV-2 as relevant triggers [231,271].

Post-infectious ON predominantly affects children and young adults, with a female predominance. The latency between infection and visual symptoms usually ranges from 1 to 6 weeks. Clinically, patients present with subacute visual loss, frequently bilateral (approximately 40–60%), often accompanied by pain on eye movement and dyschromatopsia. Optic disc edema is common, particularly in pediatric cases, although retrobulbar presentations also occur. Visual acuity at nadir is variable, with many patients presenting at or below 20/200 [272].

Magnetic resonance imaging typically demonstrates optic nerve T2 hyperintensity with gadolinium enhancement. Brain MRI usually lacks disseminated demyelinating lesions, helping distinguish post-infectious ON from MS and, in most cases, from MOGAD. Acute treatment consists of high-dose IVMP followed by a short oral taper, with IVIG or PLEX reserved for severe cases or MOG-IgG positivity. Prognosis is generally favorable, with most series reporting near-complete visual recovery within 4–8 weeks.

**Post-vaccination ON** has been described in temporal association with several vaccines, most frequently following COVID-19 immunization, as well as after other vaccines summarized in Table 8.

A large *Vaccine Safety Datalink* analysis found no increased risk of ON within biologically plausible post-vaccination windows [277], whereas analyses from the *Vaccine Adverse Event Reporting System* suggest a temporal clustering of cases within the first 6–8 weeks after vaccination, despite overall incidence remaining within expected background rates [274]. A 2023 systematic review of post-COVID-19 vaccination ON reported a mean presenting VA of approximately 20/170 and bilateral involvement in 35% of cases [279].

Clinical features include subacute unilateral or bilateral visual loss, periocular pain, dyschromatopsia, and optic nerve enhancement on MRI. Detection of MOG-IgG in a subset of patients suggests that vaccination may act as a trigger in individuals with latent autoimmune susceptibility [280]. Visual outcomes are typically favorable, and recurrences after re-exposure to the same vaccine are rare [281]. Within the ICON 2022 framework, post-infectious and post-vaccination ON most often represent monophasic immune-mediated ON, particularly in children and young adults [5,272], but antibody positivity reclassifies the phenotype as MOG-associated ON [279,280].

## 5. Diagnosis of Optic Neuritis

### 5.1. Clinical History and Examination

The diagnosis of optic neuritis (ON) relies on a careful clinical history addressing the onset of visual loss, its association with eye pain—typically exacerbated by ocular movements—and headache; the temporal evolution of visual impairment over subsequent days or weeks; the nature of visual deficits; the presence of premonitory symptoms; recent infectious illnesses or vaccinations; prior symptoms suggestive of ON mimickers; comorbid conditions; and a family history of autoimmunity. As an inflammatory disorder, ON typically presents as a subacute event, with progressive visual worsening over hours to a few days. Abrupt, non-progressive visual loss favors a vascular cause, while progression beyond one week suggests an expansive lesion.

**Eye Pain and Headache**—Eye pain and headache are hallmark symptoms of ON, with prevalence and characteristics varying by etiology. In MS-ON, eye pain is near universal, affecting up to 92% of patients in the ONTT, most often exacerbated by eye movement [26,282]. This pain is partly attributed to optic nerve sheath and anterior segment involvement, activating trigeminal afferents, and is strongly associated with orbital segment enhancement on MRI [282,283].

Pain typically precedes visual loss by two to three days and resolves within one week. In AQP4-ON, pain is less consistent and generally milder, less movement-related, and less predictive of visual decline, reflecting predominant posterior optic pathway involvement [164]. By contrast, MOG-ON shows high pain prevalence (86–92%), often bilateral, frequently preceding vision loss. Headaches are also more common in MOG-ON than MS-ON (50.5% vs. 14%), supporting a role for optic nerve sheath inflammation [164,284].

Overall, pain characteristics may assist early differentiation of ON subtypes before serological results are available.

**Visual Acuity**—Assessment of VA is a key element of the clinical history. Among immune-mediated ON subtypes, AQP4-ON is consistently associated with the most severe visual loss and poorest high-contrast VA outcomes compared with MS-ON, MOG-ON, and double-negative ON [164,200]. In a Japanese cohort of 531 noninfectious ON cases, median pretreatment VA (logMAR) was 2.6 in AQP4-ON, compared with 1.6 in MOG-ON and 1.2 in DN-ON; 53% of AQP4-ON patients had VA of CF or worse, versus 25% in DN-ON and 22% in MOG-ON [164]. Similarly, a large Chinese series of 1022 ON attacks reported VA ≤20/200 in 83.6% of AQP4-ON and 66.9% of MOG-ON attacks [159]. Nadir VA in AQP4-ON was frequently profound, with 34.9% reaching no light perception [200].

**Visual Field Abnormalities**—Visual field (VF) patterns may provide supportive but non-specific diagnostic information. While MS-ON most often produces a central scotoma, AQP4-ON is associated with more heterogeneous and extensive VF defects, reflecting frequent long-segment optic nerve and chiasmal involvement. In the Japanese nationwide cohort, central scotoma predominated in DN-ON (61%) but remained common in AQP4-ON (46%); the latter also showed higher frequencies of complete field loss (26%), altitudinal defects (22%), and temporal hemianopia (7%) [164]. Despite these trends, substantial overlap exists across ON subtypes, and no single VF pattern reliably distinguishes MS-ON, AQP4-ON, MOG-ON, or DN-ON in isolation [6,285]. These extensive defects are consistent with MRI findings in NMOSD, including longitudinally extensive optic nerve lesions and chiasmal involvement [286].

**Contrast Sensitivity and Color Vision Abnormalities**—High-contrast VA alone incompletely captures visual dysfunction in ON [287]. Low-contrast visual acuity (LCVA) and color vision testing are more sensitive for detecting functional impairment and predicting outcome. In demyelinating ON cohorts assessed ≥3 months after attack, Sloan 2.5% LCVA outperformed HCVA and 1.25% LCVA in identifying prior ON, and its combination with HCVA detected abnormalities in approximately 85% of affected eyes [288]. In the acute setting, LCVA demonstrated 100% sensitivity for ON, exceeding OCT, VEP, and MRI, while orbital MRI provided complementary diagnostic specificity [289]. Contemporary guidelines therefore recommend LCVA and structured color vision testing as core components of ON assessment [290]. Early inter-eye color asymmetry predicts long-term LCVA, axonal loss, and mfVEP abnormalities, underscoring the strong structure–function relationship in ON [291,292].

**Bilateral Involvement**—AQP4-ON shows a higher tendency for bilateral involvement than MS-ON, although it is less frequent than in MOG-ON. Attacks may be simultaneous or rapidly sequential, and bilateral ON is recognized as a characteristic feature of NMOSD [140]. In a large Chinese cohort, bilateral involvement at first presentation occurred in 12.6% of AQP4-ON attacks compared with 45.0% in MOG-ON [159]. When present, bilateral ON should strongly raise suspicion for atypical ON, particularly AQP4-ON or MOGAD-ON. Associated features such as pain, optic disc edema, and RAPD further contribute to clinical differentiation.

**Optic Disc Edema**—The frequency of optic disc edema varies according to ON etiology. In MS-ON, disc swelling occurs in approximately 35% of cases, with most patients presenting with retrobulbar neuritis and a normal optic disc [26]. Disc edema is less common in AQP4-ON than in MOG-ON. In a Chinese cohort, disc swelling was observed in 32.8% of AQP4-ON compared with 67.7% of MOG-ON cases [180], a pattern confirmed in a large Japanese multicenter study [164]. A meta-analysis reported pooled frequencies of approximately 35% in MS-ON, 20–30% in AQP4-ON, and 50–80% in MOG-ON [140]. The lower prevalence in AQP4-ON likely reflects its predilection for posterior optic nerve and chiasmal involvement.

**Relative Afferent Pupillary Defect**—The relative afferent pupillary defect (RAPD) reflects asymmetric afferent pathway dysfunction and remains a key bedside sign supporting the diagnosis of ON and helping distinguish it from retinal disorders [123]. RAPD was a mandatory inclusion criterion in the ONTT [2] and remains part of the minimal clinical triad for “possible ON,” together with subacute monocular vision loss and pain with eye movements [5]. In unilateral ON, RAPD is present in most cases, while its absence constitutes a diagnostic red flag, particularly in the presence of atypical features [123].

Accurate assessment requires optimal technique and testing conditions, which are often suboptimal in routine clinical settings. RAPD is also intrinsically insensitive in bilateral or near-symmetric ON, as commonly seen in AQP4-IgG- and MOG-IgG-associated disease [237,284].

Therefore, while highly informative, RAPD findings must be interpreted in context and complemented by multimodal investigations such as OCT, MRI, and serological testing [132,237].

### 5.2. Paraclinical and Supportive Tests

**Figure 3:**
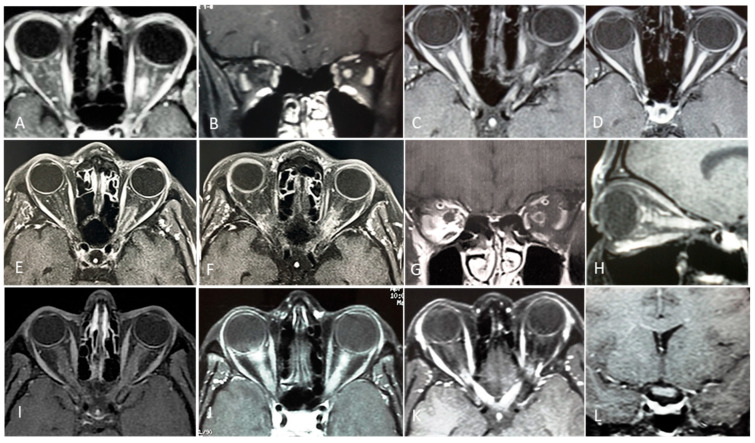
Magnetic Resonance Imaging Features of Immune-Mediated Optic Neuritis Subtypes. T1-weighted, gadolinium-enhanced orbital MRI illustrating characteristic patterns across immune-mediated optic neuritis. (**A**,**B**) MS-ON: axial (**A**) and coronal (**B**) views showing a short, focal enhancing lesion of the left optic nerve. (**C**,**D**) MOG-IgG–seronegative CRION: axial views demonstrating bilateral, longitudinally extensive enhancement of the optic nerves. (**E**–**G**) MOG-ON: axial views showing optic nerve sheath enhancement (**E**) and enhancement of the posterior orbital perioptic tissues (**F**), with coronal confirmation of perioptic enhancement (**G**). (**H**–**L**) AQP4-ON: sagittal view (**H**) showing a longitudinally extensive, thickened and enhancing optic nerve; axial views demonstrating bilateral optic nerve involvement with mild (**I**) and marked (**J**) enhancement; and axial (**K**) and coronal (**L**) views showing chiasmal involvement.

**Magnetic resonance imaging**—MRI remains indispensable for evaluating ON, providing diagnostic confirmation and enabling reliable distinction among MS-ON, AQP4-ON, and MOG-ON (Table 9; Figure 3). Conventional fat-suppressed T2-weighted and STIR sequences have limited sensitivity, detecting ON abnormalities in only 20–44% of acute episodes [5], whereas gadolinium-enhanced fat-suppressed T1-weighted sequences achieve far superior detection rates of 85–94% [293,294]. Diffusion tensor imaging offers complementary biomarkers, capturing microstructural axonal injury with predictive value for visual recovery [295].

Lesion length and topography are among the strongest radiological discriminators across immune-mediated ON subtypes. MS-ON typically demonstrates short retrobulbar lesions, whereas longitudinally extensive optic nerve lesions (LEONL) (>17–18 mm or >50% of total nerve length) are common in MOG-ON (23–88%) and AQP4-ON (50–79%) [164,211]. Segmental analysis reinforces these distinctions since ≥2-segment involvement appears in 75% of NMOSD-ON and 33% of MS-ON, whereas ≥3 segments are affected in 72% vs. only 7% of cases, respectively [296]. Such patterns reflect the broader spatial distribution of inflammation in AQP4- and MOG-ON.

Differences in the continuity and trajectory of lesions also aid diagnosis. LEONL with contiguous enhancement from orbit to chiasm is strikingly more frequent in MOG-ON (54%) compared with AQP4-ON (7%) [160] helping differentiate these two long-lesion disorders.

Laterality offers additional discriminatory value. Bilateral involvement appears in 84% of MOG-ON and 82% of AQP4-NMO cases but in only 23% of MS-ON cases [211], a finding supported by independent series [297].

Despite this shared bilateral tendency, MOG-ON and AQP4-ON present distinct anatomic predilections: MOG-ON more frequently affects anterior segments and often accompanies optic disc edema, whereas AQP4-ON preferentially involves posterior segments, including intracranial optic nerve, chiasm, and optic tracts [160,164,211,285].

Perineural enhancement is a distinctive marker of MOG-ON, observed in up to ~50% of cases [298] reflecting inflammatory involvement of the optic nerve sheath. MOG-ON is also strongly associated with perioptic orbital fat and posterior orbital tissue involvement (31–52%)—findings uncommon in AQP4-ON and extremely rare in MS-ON [299]. These periorbital features provide important radiological clues in early or atypical presentations.

Chiasmal involvement varies substantially among ON phenotypes. AQP4-ON demonstrates the highest prevalence (~20%), followed by MOG-ON (~16%) [160] while MS-ON shows substantially lower frequencies (5–15%) [160,211]. Nonetheless, the pattern of chiasmal involvement differs: in MOG-ON, chiasmal enhancement often represents an extension of a long anterior–posterior lesion, whereas in AQP4-ON it typically localizes to the posterior chiasm [160]. Modern large-scale cohorts suggest that earlier literature may have overestimated chiasmal involvement in AQP4-ON.

In combination, lesion length, the presence of perineural or orbital tissue involvement, laterality, and chiasmal extension provide robust phenotypic signatures. Consequently, thin-slice (≤3 mm) contrast-enhanced fat-suppressed orbital MRI at ≥1.5 T remains essential for accurate subtype differentiation and individualized therapeutic decision-making.

**Table 9:** Distinct MRI Features in MS-ON, AQP4-ON, and MOG-ON.

Feature	MS-ON	AQP4-ON	MOG-ON	Key References
Laterality	Usually unilateral	Often bilateral	Often bilateral	[210,211,300]
Lesion Length	Short (<1/2 optic nerve)	Longitudinally extensive	Longitudinally extensive	[210,211,300]
Optic Nerve Head Swelling	Rare	Rare	Common	[210,211,300]
Chiasmal Involvement	Rare (5–15%)	Frequent (20–64%)	Moderate (15–16%), often as part of long lesion	[210,300,301]
Optic Tract Involvement	Rare	Common (up to 45%)	Rare	[210,211,300]
Lesion Location (Optic Nerve)	Retrobulbar, posterior	Posterior, intracranial, chiasmal, tract	Anterior, retrobulbar, optic nerve head	[210,211,300]
Brain Lesions	Common	Rare	Rare	[213,298,300,302]
Central Vein Sign (CVS)	Common	Rare	Rare	[213,303]
Enhancement Pattern	Focal, well-demarcated	Patchy, poorly demarcated	Fluffy, perineural, poorly demarcated	[298,300]

MRI—magnetic resonance imaging; MS-ON—multiple sclerosis-associated optic neuritis; AQP4-ON—aquaporin 4-associated optic neuritis; MOG-ON—myelin oligodendrocyte glycoprotein antibody-associated optic neuritis; CVS—central vein sign.

While MRI is essential for etiological classification and risk stratification, it provides limited direct quantification of axonal loss and visual pathway integrity, highlighting the complementary role of OCT in structural and prognostic assessment.

**Figure 4:**
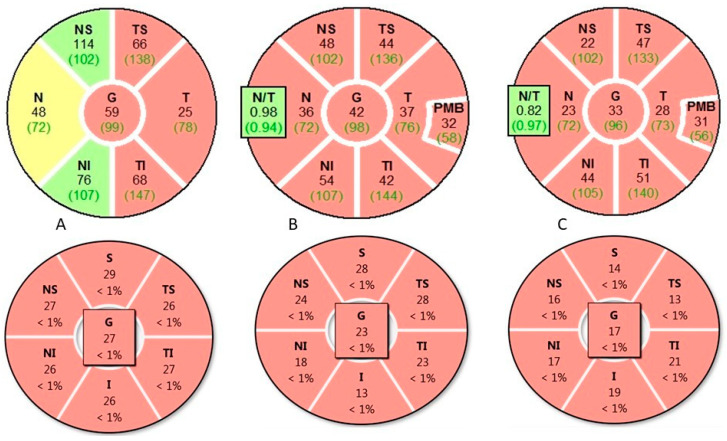
Optical coherence tomography in immune-mediated optic neuritis subtypes (OS). Upper row: peripapillary retinal nerve fiber layer thickness; lower row: macular ganglion cell (GC) layer thickness. (**A**). MS-ON; (**B**). MOG-ON; (**C**). AQP4-ON. The severity of axonal and ganglion cell loss is lowest in MS-ON, highest in AQP4-ON, and intermediate in MOG-ON. In MS-ON, the temporal quadrant of the pRNFL is preferentially and more severely affected.

**Optic coherence tomography**—OCT provides highly sensitive quantitative measures of neuroaxonal injury across ON phenotypes (Table 10; Figure 4). In MS-ON, chronic pRNFL thinning typically ranges from 60–75 μm, with marked temporal-predominant loss reflecting selective vulnerability of small-diameter papillomacular axons. GCIPL values decline to ~55–70 μm compared with 80–85 μm in healthy controls, and subclinical fellow-eye involvement is frequent, illustrating progressive and attack-independent neurodegeneration [304].

AQP4-ON displays a more destructive and diffuse pattern of retinal neurodegeneration. In the CROCTINO cohort, GCIPL thickness averaged 57.4 ± 12.2 µm versus 81.4 ± 5.7 µm in controls, with the first ON attack accounting for the vast majority of irreversible loss (−22.7 µm after the initial episode and only −3.5 µm with additional relapses) [305]. Meta-analytic data confirm similarly severe injury, with reductions of ~33 µm in pRNFL and ~21 µm in GCIPL compared with healthy controls, and thinning more pronounced than in MS-ON [306]. Although the outer retinal layers are generally preserved, except for selective parafoveal OPL thinning [307], the pattern of inner retinal involvement is highly distinctive. In this context, IED metrics provide particularly strong diagnostic discrimination, with a pRNFL IEAD ≥ 5 µm yielding an are under the curve (AUC) of ~0.95 (sensitivity 86%, specificity 82) and a GCIPL IEAD ≥ 4 µm achieving an AUC of ~0.93 (sensitivity 75%, specificity 98). These quantitative asymmetry measures have been incorporated into recently proposed ON diagnostic criteria and enhance the differentiation of AQP4-ON from both healthy controls and AQP4-NMOSD without ON [308].

MOG-ON exhibits a unique structural trajectory, beginning with marked acute optic disc and pRNFL swelling often exceeding 150–200 μm—more pronounced than in MS-ON or AQP4-ON. After edema resolves, pRNFL and GCIPL thinning are generally mild to moderate after a first episode (GCIPL 60–75 μm), though cumulative relapses lead to progressive loss [309]. Fellow-eye structure is typically preserved, consistent with an attack-dependent rather than chronic degenerative process [310,311]. Diagnostic accuracy of IED thresholds remains high in MOG-ON (pRNFL > 5 μm; GCIPL > 4 μm) [83,304,312,313].

Structure–function correlation displays striking phenotype-specific patterns. In adults with MOG-ON, each 10 μm GCIPL loss corresponds to a loss of ~5.2 high-contrast letters, yet this relationship is weaker than in AQP4-ON and MS-ON [314]. Pediatric patients show even greater dissociation: despite similar degrees of GCIPL thinning, 73% regain complete vision compared with 31% of adults, suggesting greater neuroplasticity [193,315]. A defining hallmark of MOG-ON is its pronounced structure–function dissociation. Even with substantial pRNFL and GCIPL thinning—pRNFL ≤ 75 μm in 69% of eyes, GCIPL frequently 55–65 μm—VA recovers to ≥20/30 in 70–85% of cases [215,316,317,318,319].

Conversely, AQP4-ON produces far poorer visual outcomes despite sometimes comparable structural metrics [217].

MS-ON typically exhibits more proportional structure–function coupling than MOG-ON but less severe injury than AQP4-ON.

Temporal evolution also differs across subtypes. MS-ON shows early GCIPL thinning within 2–4 weeks. In MOG-ON, pronounced pRNFL swelling masks early GCIPL loss, which becomes apparent only after 6–12 weeks [316]. In AQP4-ON, GCIPL loss is abrupt, severe, and most pronounced during the first attack, reflecting astrocytopathic mechanisms [306,316,320]. Retinal thinning correlates with degenerative changes in the lateral geniculate nucleus and occipital cortex, consistent with trans-synaptic degeneration along the visual pathway [321].

OCTA provides complementary microvascular insights. In AQP4-ON, reductions in radial peripapillary capillary density and macular superficial plexus perfusion occur even in clinically unaffected eyes and correlate closely with VA, pRNFL, and GCIPL thinning. MOG-ON may also show reduced capillary density, though with greater variability and typically milder reductions than AQP4-ON, consistent with its more reversible inflammatory profile.

**Table 10:** Comparative OCT Characteristics in MS-ON, AQP4-ON, and MOG-ON.

OCT Parameter	MS-ON	AQP4-ON	MOG-ON	Key-References
Typical chronic pRNFL thickness	60–75 µm	40–55 µm	65–85 µm after single ON	[215,217,305,322]
Typical chronic GCIPL thickness	55–70 µm	57.4 ± 12.2 µm; −22.7 µm after first attack	60–75 µm after first ON; 55–65 µm recurrent	[215,217,305]
Acute pRNFL swelling	Mild–moderate	Variable	>150–200 µm	[310,311]
Pattern of thinning	Temporal-predominant	Diffuse, pan-retinal	Global, less temporal	[310,311]
Outer retinal layers (OPL/ONL)	Preserved	No consistent thinning	Preserved	[215]
Subclinical (non-ON) eye involvement	Frequent (3–5 µm GCIPL asymmetry)	Less frequent	Rare	[305,322]
Inter-Eye Difference thresholds	GCIPL ≥ 4 µm; pRNFL ≥ 5 µm	Validated: pRNFL ≥ 5 µm (AUC 0.95)	GCIPL ≥ 7–8 µm or ≥8–10% asymetry	[308,313]
Structure–function relationship	Correlates with VA loss	Strong correlation	Dissociation possible (70–85% recover ≥20/30)	[216,217]
Disease mechanism reflected in OCT	Mixed demyelination + neurodegeneration (progressive component)	Astrocytopathy with secondary neuronal loss	Relapse-driven inflammatory demyelination with relative ganglion cell functional preservation	[215,217]

MS-ON—multiple sclerosis-associated optic neuritis; AQP4-ON—aquaporin 4-associated optic neuritis; MOG-ON—myelin oligodendrocyte glycoprotein antibody-associated optic neuritis; pRNFL—peripapillary retinal nerve fiber layer thickness; GCIPL—ganglion cell—inner plexiform layer thickness; IED—intereye difference; VA—visual acuity.

However, OCT findings must be interpreted in conjunction with MRI, as retinal structural changes alone do not reliably distinguish between MS-ON, AQP4-ON, and MOG-ON in the absence of lesion localization and CNS context.

**Visual Evoked Potentials as a Supportive Tool for Optic Neuritis Diagnosis and Subtypes Differentiation**—VEPs provide an objective physiological measure of visual pathway conduction and have enduring value in both the diagnosis of ON and the differentiation of immune-mediated ON phenotypes. VEPs quantify two key dimensions of injury, i.e., conduction delay, reflected in P100 latency prolongation, and axonal loss, reflected in a reduction in N75–P100 amplitude. The relative predominance of these abnormalities varies among MS-ON, AQP4-ON, and MOG-ON, and these patterns align with the underlying pathophysiology of each disorder.

The International Consensus on Optic Neuritis (ICON) Criteria 2022 explicitly recognize VEP as a supportive diagnostic biomarker, especially when MRI is nondiagnostic or fundoscopic findings are subtle, reinforcing its role in confirming demyelinating optic neuropathy [5].

In MS-ON, VEP predominantly demonstrate prolonged P100 latency with relatively preserved amplitude, consistent with primary demyelination and partial axonal preservation. In a comparative diagnostic study, combined multifocal VEP (mfVEP) amplitude–latency criteria detected abnormalities in ~89% of previously affected MS-ON eyes, compared with 62% by OCT and 72% by automated perimetry, underscoring VEP’s sensitivity to conduction block [323].

Moreover, mfVEP latency delay has prognostic relevance as in clinically isolated syndrome presenting with ON, latency delay predicted a 36.4% one-year conversion to MS, whereas patients with normal latency showed 0% conversion [324]. Low-contrast VEP further increases early detection sensitivity, identifying abnormalities in 76.9% of first episode ON compared with 43.6% using standard high-contrast stimuli [325].

In AQP4-ON, the VEP pattern diverges markedly. Here, severely reduced or extinguished VEP amplitude with only modest or variable latency prolongation is typical, reflecting pronounced and often irreversible axonal damage. Comparative structure–function studies demonstrate that AQP4-ON produces greater amplitude loss and worse visual outcomes than MS-ON, and unlike MS, the fellow eye is usually structurally and electrophysiologically preserved [326].

MOG-ON shows a third, intermediate profile. During the acute phase, large optic disc swelling, and conduction block commonly produce marked P100 latency prolongation, like MS-ON. However, VEP amplitude is frequently better preserved than in AQP4-ON, aligning with OCT and clinical evidence that MOG-ON involves demyelination with relative axonal sparing. Longitudinal electrophysiology confirms that latency may remain prolonged, but amplitude often recovers in parallel with visual function, producing the well-recognized structure–function dissociation [309].

### 5.3. Diagnostic Criteria for Optic Neuritis

The ICON 2022 criteria provide a pragmatic, structured framework for diagnosing optic neuritis (ON), distinguishing *possible* from *definite* ON and standardizing paraclinical corroboration for both clinical practice and research (Table 11). They represent the first international attempt to formalize diagnostic thresholds across diverse ON etiologies—including MS-ON, AQP4-IgG–positive NMOSD-ON, MOG-ON, and idiopathic ON—while integrating modern imaging and serological biomarkers.

The criteria begin with three clinical entry phenotypes: (A) subacute, typically monocular visual loss with pain on eye movement, dyschromatopsia, reduced CS, and a RAPD; (B) a painless presentation otherwise identical to (A); and (C) binocular visual loss with features of (A) or (B), noting RAPD is unreliable in bilateral disease. These clinical constellations anchor a two-tiered scheme in which paraclinical evidence—neuroimaging, OCT, or immunological markers—upgrades the diagnosis from “possible” to “definite” ON.

Paraclinical corroboration rests on three modality pillars. First, OCT: acute optic disc swelling consistent with ON, or intereye asymmetry measured within 3 months—the consortium prespecifies thresholds of mGCIPL ≥4% or >4 µm, or pRNFL >5% or >5 µm. Second, MRI of the orbits showing acute optic nerve ± sheath gadolinium enhancement, or intrinsic T2 signal increase within 3 months. Third, biomarkers, either serum antibodies (AQP4-IgG, MOG-IgG, or CRMP5-IgG) or CSF-restricted OCB providing biological plausibility and etiologic cues.

ICON then specifies how clinical and paraclinical data combine: definite ON is diagnosed with (A) + ≥1 paraclinical test; (B) + ≥2 paraclinical tests from different modalities; or (C) + two paraclinical tests, one of which must be MRI. Possible ON applies when a patient is assessed acutely with features of (A)/(B)/(C) but paraclinical tests are unavailable and the fundus and clinical course remain typical on follow-up, or when paraclinical tests are positive in a patient with a history strongly suggestive of ON. These explicit combinations address prior variability and aim to minimize both under- and over-diagnosis.

Crucially, the criteria embed “red flags” to prompt evaluation for mimickers (ischemic, compressive, infectious, toxic-nutritional, hereditary). Examples include progressive visual loss over >2 weeks without improvement, severe disc edema or, conversely, unexplained optic atrophy at onset, bilateral simultaneous ON, absence of pain, retinitis or retinal dysfunction on OCT/electrophysiology, focal neurological deficits, fever/systemic signs, and atypical demographic or geographic contexts. This structured differential reduces misclassification and guides ancillary testing.

Early external applications underscore both the utility and the operational nuances of the 2022 framework. In a first-episode cohort, Terrim et al. [131] classified 62% as definite and 38% as possible ON, with seronegative non-MS causes less often meeting “definite” thresholds—highlighting the importance of timely, multimodal testing. A major prospective evaluation arose from the Acute Optic Neuritis Network (ACON), a global, multicenter cohort assessing first-ever ON, which provided critical insights into the operational performance of the ICON criteria in real-world practice. Strict adherence to the ICON clinical entry features—particularly the requirement for RAPD and documented dyschromatopsia—excluded a substantial proportion of clinically diagnosed ON; in fact, nearly half of patients were classified as “not ON” solely because RAPD or color vision impairment was not documented, despite 63% showing MRI-confirmed optic nerve inflammation and 43% harboring disease-defining biomarkers. These observations underscore several limitations of mandatory reliance on RAPD: it is intrinsically insensitive in bilateral ON, a pattern common in NMOSD and MOGAD; it may be subtle or undetectable in mild, early, or partially treated ON; and the swinging-flashlight test is heavily dependent on examiner skill, lighting conditions, and patient cooperation. Furthermore, emergency departments—where most ON presentations occur—frequently lack standardized color vision testing, contributing to under-documentation of dyschromatopsia. In response to these constraints, the ACON investigators proposed a pragmatic refinement designed to increase diagnostic sensitivity without compromising specificity: in patients with otherwise typical painful monocular ON, the absence of RAPD or dyschromatopsia may be offset by concordant paraclinical evidence, specifically MRI abnormalities of the optic nerve plus a second paraclinical abnormality such as OCT asymmetry or a disease-defining biomarker. Applying this adjustment substantially increased ON capture (from 58% to 79%), offering constructive guidance for implementing ICON criteria across diverse clinical environments and supporting a more flexible, evidence-driven diagnostic approach [132].

**Figure 5:**
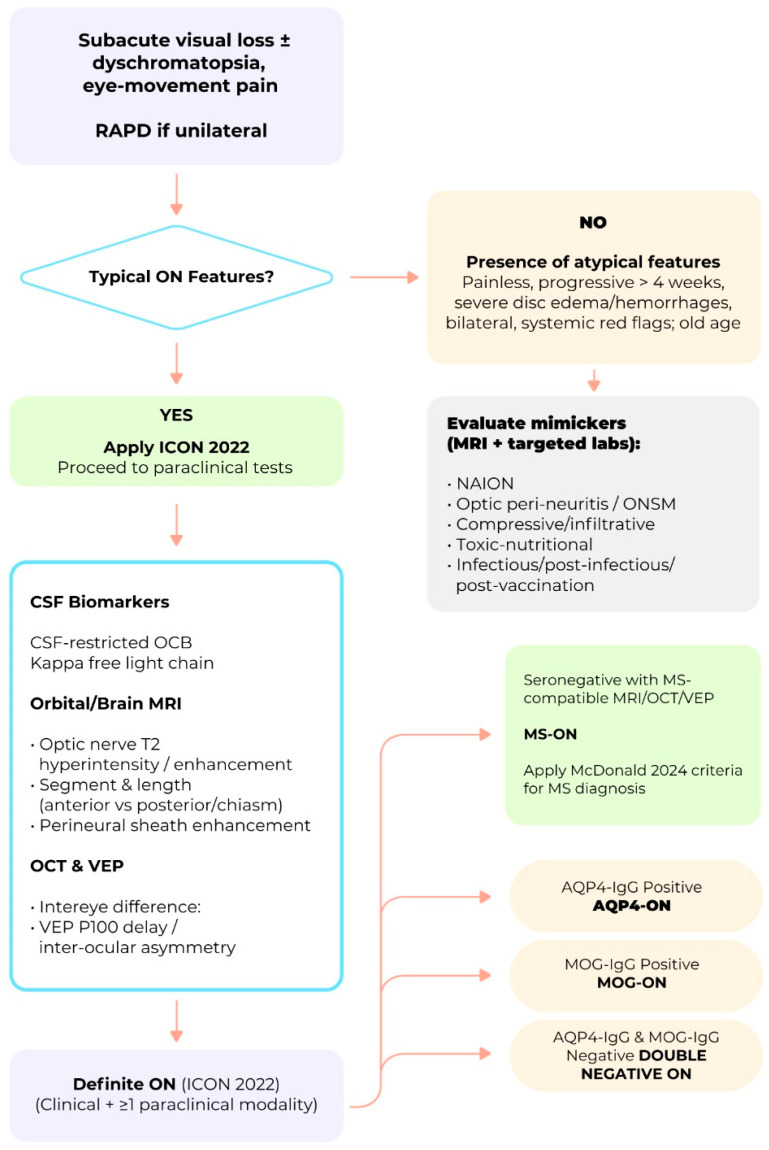
Diagnostic flowchart for immune-mediated optic neuritis subtypes RAPD—relative afferent pupillary defect; ICON—International Consensus on Optic Neuritis; CSF—cerebrospinal fluid; OCB—oligoclonal bands; OCT—optical coherence tomography; VEP—visual evoked potential; NAION—non-arteritic anterior ischemic optic neuritis; ONSM—optic nerve sheath meningioma; AQP4-ON—aquaporin 4-associated optic neuritis; MOG-ON—myelin oligodencrocyte glycoprotein-associated optic neuritis.

The structured ICON 2022 approach facilitates accurate etiologic classification of immune-mediated ON by combining clinical phenotype with OCT, MRI, and antibody testing. OCT inter-eye asymmetry and MRI enhancement support confirmation of inflammatory optic neuropathy, while serologic markers differentiate subtypes: AQP4-IgG identifies NMOSD-ON, MOG-IgG characterizes MOG-ON, and the presence of restricted-CSF OCB in specific antibody negative cases, and typical MRI abnormalities favor MS-ON. The diagnostic flowchart (Figure 5) reinforces this stepwise logic, guiding clinicians from initial recognition of typical ON signs toward diagnosis of immune-mediated ON subtypes, while prompting exclusion of ischemic, compressive, infiltrative, and toxic mimics. This framework promotes early, targeted treatment and reduces misclassification.

## 6. Unanswered Questions and Unmet Research Needs

In spite of the remarkable progress achieved over the last four decades since the ONTT, ON remains an area marked by persistent diagnostic, prognostic, and therapeutic uncertainties. The following subsections outline the principal areas in which uncertainties persist and identify priority directions for future investigation.

### 6.1. Damage and Repair in MS-ON

In MS-ON, corticosteroids accelerate visual recovery but do not prevent irreversible axonal degeneration. The imperfect relationship between lesion burden, inflammatory markers, and visual prognosis suggests that the determinants of axonal vulnerability, remyelination capacity, and neuroprotection are not fully characterized [327,328]. Addressing these gaps will require longitudinal imaging–histopathology correlation studies and clinical trials of remyelination-promoting and neuroprotective agents, including clemastine and anti-LINGO-1.

### 6.2. Double-Negative Optic Neuritis

A major unresolved challenge in ON is the subgroup of patients who test negative for both AQP4-IgG and MOG-IgG. In some cohorts, DN-ON represents the majority of cases, implying the presence of unidentified autoantibodies or non-humoral immune mechanisms [164,200,209].

Whether these forms reflect T cell–mediated optic neuropathy, microglial activation, astrocyte dysfunction, or non-canonical complement signaling is unknown. Progress in this area requires systematic antigen discovery using approaches such as proteomic epitope mapping, single-cell immune profiling, and phage-display antibody screening.

### 6.3. Biomarker Development and Standardization

Although AQP4-IgG and MOG-IgG testing remain the most robust serological tools for diagnosis of atypical ON [5], their optimal testing platforms, timing of sampling, and interpretation in low-titer or pediatric cases require standardization. Cerebrospinal fluid NF-L levels have shown promise as predictors of poor visual outcomes, yet cutoff values and temporal profiles remain poorly defined. Similarly, other potential CSF biomarkers, such as myelin basic protein, osteopontin, and chitinase-3-like-1—require multicenter validation before incorporation into clinical algorithms [329].

### 6.4. Imaging Biomarkers

MRI lesion characteristics differ across ON etiologies, yet these distinctions are not reliably reproducible across imaging centers [211]. OCT-derived inter-eye GCIPL and pRNFL asymmetry provides a sensitive marker of previous ON but requires device-independent thresholds and ancestry-adjusted interpretation [330]. The emerging role of OCT-A in detecting perfusion deficits is promising, though its diagnostic specificity remains under investigation, lacking large, prospective validation studies [5]. A coordinated effort toward standardized acquisition protocols and shared normative datasets is urgently needed.

### 6.5. Therapeutic Algorithms and Clinical Trials

Evidence-based treatment of acute attack sequencing remains underdeveloped across ON subtypes. Comparative trials of rituximab, inebilizumab, ravalizumab, and satralizumab to prevent relapses and avoid disability in AQP4-ON are limited. Escalation strategies are not standardized, particularly in recurrent MOG-ON [6,331]. Clinical trials would benefit from unified outcome measures, incorporating low-contrast VA, contrast sensitivity, OCT structural loss rates, and VEP latency changes to enable cross-study comparability.

### 6.6. Global Disparities and the for Collaborative Research Networks

Marked geographic differences exist in access to antibody testing, MRI, OCT, and biologic therapies, leading to delayed diagnosis and undertreatment in many regions, especially in low- and middle-income countries. Consequently, diagnostic delays and under-treatment are common [5,329].

Building multinational ON registries and biorepositories encompassing diverse ancestries is vital to capture population-specific immunological and genetic determinants. Collaborative consortia should prioritize harmonized data collection, equitable technology transfer, and cost-effective diagnostic platforms to ensure that advances in ON research translate into global clinical benefit.

## 7. Conclusions

The Optic Neuritis Treatment Trial (ONTT) established the benchmark clinical phenotype of acute demyelinating ON, demonstrated that intravenous methylprednisolone accelerates—but does not ultimately change—the visual outcome, and clarified the long-term risk of MS after the first ON episode. At that time, ON was largely conceptualized as a monospecific demyelinating event within the MS spectrum. Subsequent discovery of AQP4-IgG and MOG-IgG, together with refined MRI and OCT techniques, has transformed this view, revealing a group of distinct autoimmune optic neuropathies with different immunopathological mechanisms, clinical trajectories, and treatment responses.

Yet, several critical gaps remain. The immunobiology of DN-ON is still poorly understood; here, targeted antigen-discovery programs, single-cell immunophenotyping, and integrative transcriptomic/epigenomic studies represent key investigative avenues.

In MS-ON, longitudinal cohorts combining high-resolution MRI, OCT, VEP, and fluid biomarkers are needed to dissect the determinants of axonal vulnerability, remyelination capacity, and incomplete visual recovery and to test candidate neuroprotective or remyelinating agents in well-designed trials.

For biomarkers, research priorities include defining disease- and age-adjusted thresholds for neurofilament light chain, validating multimarker CSF and serum panels, and embedding standardized biobanking within multicenter ON registries.

In the domain of imaging, international efforts should focus on harmonizing MRI and OCT acquisition protocols, developing device-independent inter-eye difference thresholds, and clarifying the diagnostic and prognostic value of OCT angiography across ON subtypes.

Regarding therapy, comparative and stratified randomized controlled trials are required to optimize the sequencing and duration of B-cell-depleting and complement- or interleukin-targeting agents in AQP4-ON and MOG-ON, as well as to define evidence-based approaches for relapsing seronegative ON.

Finally, addressing global disparities will demand multinational registries, context-adapted diagnostic algorithms, and evaluation of cost-effective testing platforms to ensure that advances in classification and treatment translate into improved outcomes worldwide.

Taken together, these converging research programs—spanning immunology, imaging, biomarkers, therapeutics, and health equity—provide a concrete roadmap toward truly phenotype-driven, personalized care for patients with ON globally.

## Figures and Tables

**Table 1:** Baseline Demographic and Clinical Characteristics of Patients with Optic Neuritis Enrolled in the ONTT.

Characteristics	Value
Number of patients	457
Age, mean (range), years	32 (18–45)
Sex, female (%)	352 (77)
Race (%)	White: 388 (85) Black: 64 (14) Other: 5 (1%)
Affected eye (%)	Right: 229 (50), Left: 228 (50)
Simultaneous bilateral involvement (%)	7 (1.5)
Eye pain or headache associated with vision loss (%)	420 (92)
Visual acuity in affected eye %	≥20/20: 3 20/25–20/40: 6 20/50–20/190: 56 ≤20/200: 35; CF: 14; HM: 10; LP: 3; NLP: <1
Visual acuity in fellow eye (%)	≥20/20: 96 20/25–20/40: 3 ≤20/50: 1
Color vision defect in affected eye (Hardy–Rand–Rittler plates)	Median correct plates: 1/14 >90% abnormal, severe defect in 60%
Contrast sensitivity abnormality (Pelli–Robson chart)	Mean log cs: 1.04 ± 0.37 >95% abnormal (<1.65)
Visual field defect in affected eye %	Central or cecocentral scotoma: 52 Diffuse depression: 29 Nerve fiber bundle defect: 8 Altitudinal defect: 3 Other: 8
Afferent pupillary defect %	Present: >95 Severe: 47 Moderate: 34 Mild: 14

ONTT—Optic Neuritis Treatment Trial; APD—afferent pupillary defect; VA—visual acuity; LP—light perception; CS—contrast sensitivity.

**Table 2:** Evolution of the McDonald’s Criteria and Role of Optic Neuritis in the Diagnosis of Multiple Sclerosis.

McDonald’s Criteria	Key Revisions to McDonald Criteria	Impact of Optic Neuritis on MS Diagnosis	References
2001	First introduction of McDonald criteria; allowed MS diagnosis after a single clinical attack (e.g., ON) with MRI evidence of DIS and DIT.	ON recognized as a possible first demyelinating event; MRI could confirm MS diagnosis if DIS and DIT criteria met.	[59]
2005	Revised criteria allowed DIT to be demonstrated by a new T2 or gadolinium-enhancing lesion on follow-up MRI, replacing the need for clinical evidence of a second attack.	ON remained a valid first demyelinating event; follow-up MRI showing new lesions could expedite MS diagnosis.	[63]
2010	DIT could be demonstrated by simultaneous presence of gadolinium-enhancing and non-enhancing lesions on baseline MRI. Allowed earlier diagnosis without waiting for new clinical attacks or follow-up scans.	ON with baseline MRI fulfilling DIS and DIT criteria could establish MS diagnosis immediately.	[60]
2017	CSF-specific OCBs accepted as an alternative to demonstrating DIT. DIS still required.	ON plus MRI showing DIS and positive CSF OCBs could confirm MS diagnosis without requiring DIT.	[61]
2025	Advanced imaging incorporated: CVS and PRLs recognized as supportive MRI features; inclusion of OCT parameters; CSF KFLC accepted as alternative to OCBs; broader lesion topography definitions.	ON diagnosis can be supported by advanced MRI markers (CVS, PRLs), OCT evidence of retinal nerve fiber layer loss, and CSF KFLC positivity, enabling earlier and more accurate MS diagnosis when combined with DIS criteria.	[62]

ON—optic neuritis; DIS—dissemination in space; DIT—dissemination in time; CSF—cerebrospinal fluid; OCB—oligoclonal bands; KFLCs—kappa free light chains; CVS—central vein sign; PRLs—paramagnetic rim lesions; OCT—optic coherence tomography.

**Table 4:** Distinctive features of Immune-mediated Optic Neuritis Subtypes.

Feature	MS-ON	AQP4-ON	MOG-ON	Key References
Ocular Pain (%)	~10%	Lower than MOG-ON	62.5%; higher than AQP4-ON	[164,208]
Severity of VA Loss (Acute)	Mild-moderate	Severe, often profound	Severe, but often recovers	[163,164,200]
Mean Final VA	20/25–20/40	Counting fingers or worse	20/25	[164,200,209]
Response to Treatment	Good, steroid-responsive	Poorer, limited recovery	Good, steroid-responsive	[163,164,200,209]
Optic Disc Edema (Acute, %)	<33%	~9%	53–91%	[164,210]
Optic Nerve Extensive Lesion (%)	15	64	53	[211]
Perineuritis (%)	Rare	Rare	33	[163,211,212]
Chiasmal Involvement (%)	15	20–64	5–16	[210,211]
MRI Lesion Location	Anterior, unilateral	Posterior, bilateral	Anterior, bilateral	[211,213]
Optic Nerve Enhancement	Focal, short	Longitudinal, posterior	Longitudinal, anterior	[211,213]
Other MRI Features	Brain lesions common	Spinal cord, chiasm	Isolated ON, less brain involvement	[164,213]
Mean pRNFL (μm)	~87.5	58–64	58–75	[209,213,214,215,216]
Mean GCIPL (μm)	Higher than AQP4/MOG	Lower than MS	Lower than MS	[217]
OCT Pattern of Loss	Thinner RNFL, less severe	Severe thinning	Severe thinning, but better preserved	[214,217]
OCT-A Differential	Not well defined	Reduced vessel density	Reduced vessel density	[216]
CSF OCB (%)	77–95	10–30	7–17	[198,218,219,220,221]
CSF Pleocytosis (cells/μL)	Rare	25%	37.5%	[222]

RNFL—retinal nerve fiber layer; GCIPL—ganglion cell inner plexiform layer; OCT-A—optical coherence tomography angiography; CSF—cerebrospinal fluid; OCB—oligoclonal bands.

**Table 5:** Diagnostic Criteria for Chronic Relapsing Inflammatory Optic Neuropathy *.

**HISTORY**	OPTIC NEURITIS AND AT LEAST ONE RELAPSE
**CLINICAL**	Objective evidence for loss of visual function
**LABORATORY**	AQP4-IgG seronegative status
**MRI**	Contrast enhancement of the acutely inflamed optic nerves
**TREATMENT**	Response to immunosuppressive treatment and relapse on withdrawal or dose reduction

* [150,225].

**Table 6:** Systemic Autoimmune Diseases Associated with Optic Neuritis.

Disease	Report Frequency *	Typical ON Pattern/Comment	Key References (DOI)
Sarcoidosis	Frequent	Granulomatous ON often chiasmal and leptomeningeal involvement.	[240]
Systemic lupus erythematosus	Frequent	Severe, ON or ischemic neuropathy; often bilateral and poor visual outcome.	[241]
Primary Sjögren’s syndrome	Frequent	ON or optic neuropathy possibly preceding sicca; may overlap with NMOSD.	[242]
Behçet’s disease	Rare	Uveitis and retinal vasculitis typical; ON rare but documented.	[243]
ANCA-associated vasculitis (GPA/MPA/EGPA)	Rare	Orbital inflammation or vasculitic ON, sometimes bilateral.	[244]
IgG4-related disease	Rare	Optic perineuritis or sheath involvement; enhancement along optic nerve; steroid responsiveness.	[245]
Polyarteritis nodosa	Rare	ON or Ischemic neuropathy.	[246]
Takayasu arteritis	Rare	ON only occasionally reported. Mainly ischemic optic neuropathy secondary to large-vessel stenosis.	[247]
Drug-induced ON (TNF-α or checkpoint inhibitors)	Rare	Immune-mediated ON after biologic therapy (e.g., infliximab, nivolumab).	[248]
Primary antiphospholipid syndrome	Very rare	Vaso-occlusive or ischemic optic neuropathy; usually secondary to microthrombosis.	[249]
Giant cell arteritis	Very rare	True ON extremely rare. Arteritic AION dominant.	[250]
Cogan syndrome	Very rare	ON exceptional; typical ocular sign is interstitial keratitis.	[251]
Ankylosing spondylitis (HLA-B27)	Very rare	Uveitis typical; ON very rare.	[252]
Ulcerative colitis	Very rare	Immune-mediated ON after biologic therapy (e.g., infliximab, nivolumab).	[248]
Systemic sclerosis	Very rare	Sporadic optic neuropathy; likely ischemic.	[253]
Overlap or rheumatic disease (unspecified CTD)	Very rare	Mixed connective-tissue disease–related ON; anecdotal.	[254]
Kawasaki disease	Very rare	Mostly pediatric; isolated ON exceptional.	[255]
Susac syndrome	Very rare	Retinal artery occlusions typical; ON not a core feature.	[256]

* Frequent: Supported by cohort studies and systematic reviews; optic nerve involvement appears in 3–10% of systemic cases. Rare: Documented in small but reproducible clusters—roughly a few dozen reported worldwide. Very Rare: Only isolated or single-case literature; prevalence likely <1% of total systemic cases.

**Table 7:** Diagnostic framework for sarcoidosis-related optic neuritis and optic perineuritis.

Diagnostic Domain	Definite S-ON/S-OPN	Probable S-ON/S-OPN	Possible S-ON/S-OPN
Clinical phenotype	Acute or subacute optic neuritis or optic perineuritis with visual loss and/or periorbital pain; may coexist with uveitis, cranial neuropathies, or meningitic symptoms	Same as definite	Same as definite
MRI findings	Gadolinium-enhancing optic nerve sheath or optic nerve lesion; often longitudinal, circumferential, or contiguous with meningeal enhancement (‘tram-track’/‘doughnut’ signs)	Same pattern; may be less extensive or limited to leptomeningeal enhancement	Suggestive but non-specific imaging pattern; no tissue confirmation
Systemic and laboratory support	Typical systemic sarcoidosis evidence (bilateral hilar/mediastinal lymphadenopathy, ↑ ACE, ↑ sIL-2R, ↑ lysozyme); CSF lymphocytic pleocytosis or ↑ protein; FDG-PET uptake in sarcoid-like distribution	Same as definite	Suggestive systemic features or incomplete work-up
Histological confirmation	Non-caseating granulomas in neural tissue (optic nerve/sheath, meninges, brain, or ocular tissue)	Non-neural tissue biopsy (e.g., lymph node, skin, salivary gland) showing non-caseating granulomas	No biopsy or non-diagnostic specimen
Exclusion of mimics	Infectious (TB, syphilis, fungi), neoplastic (meningioma, lymphoma), and immune-mediated (NMOSD, MOGAD, IgG4-RD, GPA) causes ruled out	Same	Same
Level of certainty (Neurosarcoidosis Consortium tier)	Definite neurosarcoidosis	Probable neurosarcoidosis	Possible neurosarcoidosis
Clinical implication	Confirms sarcoid origin of optic neuropathy; guides long-term systemic therapy	Supports diagnosis when neural biopsy unavailable; requires systemic evaluation	Suggests sarcoid etiology when all mimics excluded; biopsy pursuit recommended

S-ON—sarcoidosis-associated optic neuritis. S-OPN—sarcoidosis-associated optic perineuritis. ↑—increased, ACE—angiotensin converting enzyme. sIL-2R—serum interlekin-2 receptor, FDG-PET—fluorodeoxyglucose positron emission tomography. IgG4-RD—IgG4-related disease. GPA granulomatosis with polyangiitis.

**Table 8:** Vaccines Associated with Optic Neuritis.

Vaccine Type	Evidence/Notes	References
Influenza (seasonal, H1N1)	Reviews and case series	[115,273]
Hepatitis B	Highly reported in VAERS ON	[115,274]
Human Papillomavirus (HPV)	Noted in VAERS and case reviews	[115,274]
Hepatitis A	Documented in reviews	[115]
Rabies	Documented in reviews	[115]
Measles, Rubella, Measles-Rubella	Case reports and reviews	[115,275]
Varicella Zoster (VZV)	Case reports	[276]
Yellow Fever	Documented in reviews	[115]
Anthrax	Case reports	[115,277]
Meningococcus	Documented in reviews	[115]
Tetanus, Diphtheria, Pertussis (Tdap)	VAERS, case reports, and reviews	[113,114,115,274]
COVID-19 (mRNA, J&J)	Cohort studies and case reports	[114,278,279]

Abbreviations: HPV—Human Papillomavirus; VAERS—Vaccine Adverse Event Reporting System; ON optic neuritis; VZV—Varicella Zoster; Tdap—Tetanus, Diphtheria, Pertussis.

**Table 11:** Optic Neuritis Diagnostic Criteria *.

Component	Description	Key Elements/Thresholds
Clinical Entry Phenotypes	Defines clinical presentations consistent with ON.	Subacute monocular visual loss, pain with eye movement, dyschromatopsia, reduced contrast sensitivity, RAPD. Same as A but without pain. Bilateral involvement; note: RAPD unreliable.
Core Clinical Features Suggestive of ON	Used to support bedside impression of ON.	Subacute vision loss (hours–days); pain on eye movement (usually); impaired color vision; reduced contrast sensitivity; central/cecocentral scotoma; RAPD (if unilateral/asymmetric).
Paraclinical Modalities	Objective confirmation elevates diagnosis from possible to definite.	(1) OCT: Acute optic disc edema OR Inter-eye asymmetry within 3 months: mGCIPL ≥4% or >4 μm pRNFL ≥5% or >5 μm. (2) MRI (Orbits, Gd/T2): Gadolinium enhancement or T2 hyperintensity within 3 months. (3) Biomarkers: AQP4-IgG, MOG-IgG, CRMP5-IgG or CSF-restricted OCB.
Diagnostic Categories	Defines how clinical + paraclinical criteria combine.	Definite ON: (A) + ≥1 paraclinical test OR (B) + ≥2 different paraclinical modalities OR (C) + ≥2 paraclinical tests, one must be MRI. Possible ON: Typical clinical presentation but insufficient evidence yet OR Historical ON strongly suggested without contemporaneous testing.
Red Flags	Features requiring reconsideration of ON diagnosis to avoid misclassification.	Progressive visual decline > 2 weeks; painless ON; absence of RAPD in unilateral cases; severe disc edema or early optic atrophy; simultaneous bilateral ON; retinal findings inconsistent with ON; systemic infectious signs; compressive or ischemic patterns.

* Modified from Petzold et al., 2022 [5]. ON—optic neuritis; RAPD—relative afferent pupillary defect; CS—contrast sensitivity; mGCIPL—macular ganglion cell-inner plexiform layer; pRNFL—peripapillary retinal nerve fiber layer.

## Data Availability

No new data were created or analyzed in this study. Data sharing is not applicable to this article.

## Abbreviations

The following abbreviations are used in this manuscript:

ACE	Angiotensin-conversion enzyme
ACTH	Adrenocorticotropic hormone
APD	Afferent pupillary defect
AQP4 IgG	Aquaporin-4
AQP4-ON	Aquaporin-4-associated optic neuritis
ARR	Annualized relapse rate
AUC	Area under the curve
CF	Count fingers
CRION	Chronic relapsing inflammatory optic neuropathy
CRMP5	Collapsin response-mediator protein 5
CS	Contrast sensitivity
CSF	Cerebrospinal fluid
CVS	Central vein sign
DAMPs	Damage-associated molecular patterns
DIS	Dissemination in space
DIT	Dissemination in time
DN-ON	Double negative optic neuritis
EBV	Epstein–Barr virus
EDSS	Expanded disability status scale
GCIPL	Ganglion cell internal plexiform layer
GFAP	Glial fibrillary acidic protein
GFAP-A	GFAP astrocytopathy
GFAP-ON	GFAP-associated-ON
HCVA	High-contrast visual acuity
HM	Hand motion
HPV	Human papillomavirus
ICON	International consensus on optic neuritis
IEAD	Intereye absolute difference
IED	Intereye difference
IEF	Isoelectric focusing
IEPD	Intereye percentage difference
IVIG	intravenous immunoglobulin
IVMP	IV methylprednisolone
IWOS	International Workshop on Ocular Sarcoidosis
KFLCs	Kappa free light chains
LCVA	Low-contrast visual acuity
LEON	Longitudinally extensive optic nerve
LP	Light perception
mfVEP	Multifocal VEP
MOG IgG	Myelin oligodendrocyte glycoprotein
MOGAD	Myelin oligodendrocyte glycoprotein antibody-associated disease
MOG-ON	Myelin oligodendrocyte glycoprotein-associated optic neuritis
MRI	Magnetic resonance imaging
MS	Multiple sclerosis
MS-ON	Multiple sclerosis-associated optic neuritis (Typical optic neuritis)
NPL	No perception of light
NMOSD	Neuromyelitis optica spectrum disorder
non-MS-ON	Optic neuritis not associated with MS (Atypical optic neuritis))
OCBs	Oligoclonal bands
OCT	Optical coherence tomography
OCT-A	Optic coherence tomography angiography
ON	Optic neuritis
ONSG	Optic Neuritis Study Group
ONTT	Optic neuritis treatment trial
OPN	Optic perineuritis
PLEX	Plasma exchange
PRLs	Paramagnetic rim lesions
pRNFL	Peripapillary retinal nerve fiber layer
RAPD	Relative afferent pupillary defect
RION	Relapsing isolated optic neuritis
RNFL	Retinal nerve fiber layer
RON	Relapsing ON
SS	Sjogren syndrome
Tdap	Tetanus, diphtheria, pertussis
VA	Visual acuity
VAERS	Vaccine Adverse Event Reporting System
VEP	Visual evoked potential
VZV	Varicella zoster virus

## References

[1] von Staden H. (1989). Herophilus: The Art of Medicine in Early Alexandria: Edition, Translation and Essays.

[2] Beck R.W., Cleary P.A., Anderson M.M., Keltner J.L., Shults W.T., Kaufman D.I., Buckley E.G., Corbett J.J., Kupersmith M.J., Miller N.R. (1992). A randomized, controlled trial of corticosteroids in the treatment of acute optic neuritis. The Optic Neuritis Study Group. N. Engl. J. Med..

[3] Lennon V.A., Kryzer T.J., Pittock S.J., Verkman A.S., Hinson S.R. (2005). IgG marker of optic-spinal multiple sclerosis binds to the aquaporin-4 water channel. J. Exp. Med..

[4] Kitley J., Woodhall M., Waters P., Leite M.I., Devenney E., Craig J., Palace J., Vincent A. (2012). Myelin-oligodendrocyte glycoprotein antibodies in adults with a neuromyelitis optica phenotype. Neurology.

[5] Petzold A., Fraser C.L., Abegg M., Alroughani R., Alshowaeir D., Alvarenga R., Andris C., Asgari N., Barnett Y., Battistella R. (2022). Diagnosis and classification of optic neuritis. Lancet Neurol..

[6] De Lott L.B., Bennett J.L., Costello F. (2022). The changing landscape of optic neuritis: A narrative review. J. Neurol..

[7] Benard-Seguin E., Costello F. (2023). Optic neuritis: Current challenges in diagnosis and management. Curr. Opin. Neurol..

[8] Kirk G.S., Raven J.E., Schofield M. (1983). The Presocratic Philosophers: A Critical History with a Selection of Texts.

[9] Hippocrates (2012). Volume X: Aphorisms and Other Works.

[10] Pormann P.E., Savage-Smith E. (2007). Medieval Islamic Medicine.

[11] Temkin O. (1973). Galenism: Rise and Decline of a Medical Philosophy.

[12] Goldstein I. (1929). The Book of the Ten Treatises on the Eye Ascribed to Hunain Ibn Is-Haq (809-877 A. D.). Arch. Ophthalmol..

[13] Wikimedia Hunayn Ibn Ishaq 9th century CE Description of the Eye Diagram in a Copy of His Book, Kitab al-Ashr Maqalat fil-Ayn (Ten Treatises on the Eye), in a 12th Century CE Edition. https://commons.wikimedia.org/w/index.php?title=File:Hunayn_ibn_Ishaq_9th_century_CE_description_of_the_eye_diagram_in_a_copy_of_his_book,_Kitab_al-Ashr_Maqalat_fil-Ayn_(Ten_Treatises_on_the_Eye),_in_a_12th_century_CE_edition.jpg&oldid=844124575.

[14] Wikimedia Double Opening of the ‘Memorandum for Oculists’ by Ali Ibn Isa al-Kahhal (CBL Ar 4002, ff.103b-104a).jpg. https://commons.wikimedia.org/w/index.php?title=File:Double_Opening_of_the_%27Memorandum_for_Oculists%27_by_Ali_Ibn_Isa_al-Kahhal_(CBL_Ar_4002,_ff.103b-104a).jpg&oldid=1032259355.

[15] Gruner O.C. (1973). A Treatise on the Canon of Medicine of Avicenna: Incorporating a Translation of the First Book.

[16] Lindberg D.C. (1976). Theories of Vision from Al-kindi to Kepler.

[17] Compston A., Lassmann H., McDonald I., Compston A., Confavreux C., Lassmann H., McDonald I., Miller D., Noseworthy J., Smith K., Wekerle H. (2006). Chapter 1—The story of multiple sclerosis. McAlpine’s Multiple Sclerosis.

[18] Nettleship E. (1884). On cases of retro-ocular neuritis. Trans. Ophthal Soc. UK.

[19] Opara J.A., Brola W., Wylegala A.A., Wylegala E. (2016). Uhthoff`s phenomenon 125 years later—What do we know today?. J. Med. Life.

[20] Zalc B. (2018). One hundred and fifty years ago Charcot reported multiple sclerosis as a new neurological disease. Brain.

[21] Kahana E., Alter M., Feldman S. (1976). Optic neuritis in relation to multiple sclerosis. J. Neurol..

[22] Arnason B.G. (1973). Editorial: Optic neuritis and multiple sclerosis. N. Engl. J. Med..

[23] Alshyaokh F., Alhussein M., Almulla A., Alhelal F., Alqhtani M., Abulaban A. (2024). 3. Outcome of Multiple Sclerosis with and without Optic Neuritis. Mult. Scler. Relat. Disord..

[24] Smith E.L. (1953). Intravenous use of corticotropin in optic neuritis. A.M.A. Arch. Ophthalmol..

[25] Rawson M.D., Liversedge L.A. (1969). Treatment of retrobulbar neuritis with corticotrophin. Lancet.

[26] The Optic Neuritis Study Group (1991). The clinical profile of optic neuritis. Experience of the Optic Neuritis Treatment Trial. Optic Neuritis Study Group. Arch. Ophthalmol..

[27] Beck R.W., Group T.O.N.S. (1992). Corticosteroid treatment of optic neuritis. Neurology.

[28] Beck R.W., Cleary P.A., Trobe J.D., Kaufman D.I., Kupersmith M.J., Paty D.W., Brown C.H. (1993). The effect of corticosteroids for acute optic neuritis on the subsequent development of multiple sclerosis. The Optic Neuritis Study Group. N. Engl. J. Med..

[29] Beck R.W., Kupersmith M.J., Cleary P.A., Katz B. (1993). Fellow eye abnormalities in acute unilateral optic neuritis. Experience of the optic neuritis treatment trial. Ophthalmology.

[30] Beck R.W., Arrington J., Murtagh F.R., Cleary P.A., Kaufman D.I. (1993). Brain magnetic resonance imaging in acute optic neuritis. Experience of the Optic Neuritis Study Group. Arch. Neurol..

[31] Cleary P.A., Beck R.W., Anderson M.M., Kenny D.J., Backlund J.Y., Gilbert P.R. (1993). Design, methods, and conduct of the Optic Neuritis Treatment Trial. Control Clin. Trials.

[32] Keltner J.L., Johnson C.A., Beck R.W., Cleary P.A., Spurr J.O. (1993). Quality control functions of the Visual Field Reading Center (VFRC) for the Optic Neuritis Treatment Trial (ONTT). Control Clin. Trials.

[33] Beck R.W., Cleary P.A. (1993). Recovery from severe visual loss in optic neuritis. Arch. Ophthalmol..

[34] Beck R.W., Diehl L., Cleary P.A. (1993). Optic Neuritis Study Group. Pelli-Robson normative data for young adults. Clin. Vis. Sci..

[35] Chrousos G.A., Kattah J.C., Beck R.W., Cleary P.A. (1993). Side effects of glucocorticoid treatment. Experience of the Optic Neuritis Treatment Trial. JAMA.

[36] Beck R.W., Cleary P.A. (1993). Optic neuritis treatment trial. One-year follow-up results. Arch. Ophthalmol..

[37] Beck R.W., Cleary P.A., Backlund J.C. (1994). The course of visual recovery after optic neuritis. Experience of the Optic Neuritis Treatment Trial. Ophthalmology.

[38] Beck R.W. (1995). The optic neuritis treatment trial: Three-year follow-up results. Arch. Ophthalmol..

[39] Beck R.W., Trobe J.D. (1995). The Optic Neuritis Treatment Trial. Putting the results in perspective. The Optic Neuritis Study Group. J. Neuro-Ophthalmol. Off. J. N. Am. Neuro-Ophthalmol. Soc..

[40] Rolak L.A., Beck R.W., Paty D.W., Tourtellotte W.W., Whitaker J.N., Rudick R.A. (1996). Cerebrospinal fluid in acute optic neuritis: Experience of the optic neuritis treatment trial. Neurology.

[41] Trobe J.D., Beck R.W., Moke P.S., Cleary P.A. (1996). Contrast sensitivity and other vision tests in the optic neuritis treatment trial. Am. J. Ophthalmol..

[42] Cleary P.A., Beck R.W., Bourque L.B., Backlund J.C., Miskala P.H. (1997). Visual symptoms after optic neuritis. Results from the Optic Neuritis Treatment Trial. J. Neuro-Ophthalmol. Off. J. N. Am. Neuro-Ophthalmol. Soc..

[43] The Optic Neuritis Study Group (1997). The 5-year risk of MS after optic neuritis. Experience of the optic neuritis treatment trial. Neurology.

[44] The Optic Neuritis Study Group (1997). Visual function 5 years after optic neuritis: Experience of the Optic Neuritis Treatment Trial. The Optic Neuritis Study Group. Arch. Ophthalmol..

[45] Cole S.R., Beck R.W., Moke P.S., Kaufman D.I., Tourtellotte W.W. (1998). The predictive value of CSF oligoclonal banding for MS 5 years after optic neuritis. Optic Neuritis Study Group. Neurology.

[46] Long D.T., Beck R.W., Moke P.S., Blair R.C., Kip K.E., Gal R.L., Katz B.J. (2001). The SKILL Card test in optic neuritis: Experience of the Optic Neuritis Treatment Trial. Smith-Kettlewell Institute Low Luminance. Optic Neuritis Study Group. J. Neuro-Ophthalmol. Off. J. N. Am. Neuro-Ophthalmol. Soc..

[47] Beck R.W., Trobe J.D., Moke P.S., Gal R.L., Xing D., Bhatti M.T., Brodsky M.C., Buckley E.G., Chrousos G.A., Corbett J. (2003). High- and low-risk profiles for the development of multiple sclerosis within 10 years after optic neuritis: Experience of the optic neuritis treatment trial. Arch. Ophthalmol..

[48] The Optic Neuritis Study Group (2008). Visual function 15 years after optic neuritis: A final follow-up report from the Optic Neuritis Treatment Trial. Ophthalmology.

[49] The Optic Neuritis Study Group (2008). Multiple sclerosis risk after optic neuritis: Final optic neuritis treatment trial follow-up. Arch. Neurol..

[50] Keltner J.L., Johnson C.A., Cello K.E., Dontchev M., Gal R.L., Beck R.W. (2010). Visual field profile of optic neuritis: A final follow-up report from the optic neuritis treatment trial from baseline through 15 years. Arch. Ophthalmol..

[51] Keltner J.L., Johnson C.A., Spurr J.O., Beck R.W. (1993). Baseline visual field profile of optic neuritis. The experience of the optic neuritis treatment trial. Optic Neuritis Study Group. Arch. Ophthalmol..

[52] Beck R.W., Gal R.L., Bhatti M.T., Brodsky M.C., Buckley E.G., Chrousos G.A., Corbett J., Eggenberger E., Goodwin J.A., Katz B. (2004). Visual function more than 10 years after optic neuritis: Experience of the optic neuritis treatment trial. Am. J. Ophthalmol..

[53] Beck R.W., Smith C.H., Gal R.L., Xing D., Bhatti M.T., Brodsky M.C., Buckley E.G., Chrousos G.A., Corbett J., Eggenberger E. (2004). Neurologic impairment 10 years after optic neuritis. Arch. Neurol..

[54] Heussinger N., Kontopantelis E., Gburek-Augustat J., Jenke A., Vollrath G., Korinthenberg R., Hofstetter P., Meyer S., Brecht I., Kornek B. (2015). Oligoclonal bands predict multiple sclerosis in children with optic neuritis. Ann. Neurol..

[55] Lin Y.C., Yen M.Y., Hsu W.M., Lee H.C., Wang A.G. (2006). Low conversion rate to multiple sclerosis in idiopathic optic neuritis patients in Taiwan. Jpn. J. Ophthalmol..

[56] Elpers C., Amler S., Grenzebach U., Allkemper T., Fiedler B., Schwartz O., Meuth S.G., Kurlemann G. (2015). Prediction of Multiple Sclerosis after Childhood Isolated Optic Neuritis. Neonatal Pediatr. Med..

[57] Zimmermann H.G., Knier B., Oberwahrenbrock T., Behrens J., Pfuhl C., Aly L., Kaminski M., Hoshi M.M., Specovius S., Giess R.M. (2018). Association of Retinal Ganglion Cell Layer Thickness With Future Disease Activity in Patients With Clinically Isolated Syndrome. JAMA Neurol..

[58] Pihl-Jensen G., Wanscher B., Frederiksen J.L. (2021). Predictive value of optical coherence tomography, multifocal visual evoked potentials, and full-field visual evoked potentials of the fellow, non-symptomatic eye for subsequent multiple sclerosis development in patients with acute optic neuritis. Mult. Scler. J..

[59] McDonald W.I., Compston A., Edan G., Goodkin D., Hartung H.P., Lublin F.D., McFarland H.F., Paty D.W., Polman C.H., Reingold S.C. (2001). Recommended diagnostic criteria for multiple sclerosis: Guidelines from the International Panel on the diagnosis of multiple sclerosis. Ann. Neurol..

[60] Polman C.H., Reingold S.C., Banwell B., Clanet M., Cohen J.A., Filippi M., Fujihara K., Havrdova E., Hutchinson M., Kappos L. (2011). Diagnostic criteria for multiple sclerosis: 2010 revisions to the McDonald criteria. Ann. Neurol..

[61] Thompson A.J., Banwell B.L., Barkhof F., Carroll W.M., Coetzee T., Comi G., Correale J., Fazekas F., Filippi M., Freedman M.S. (2018). Diagnosis of multiple sclerosis: 2017 revisions of the McDonald criteria. Lancet Neurol..

[62] Montalban X., Lebrun-Frénay C., Oh J., Arrambide G., Moccia M., Pia Amato M., Amezcua L., Banwell B., Bar-Or A., Barkhof F. (2025). Diagnosis of multiple sclerosis: 2024 revisions of the McDonald criteria. Lancet Neurol..

[63] Polman C.H., Reingold S.C., Edan G., Filippi M., Hartung H.P., Kappos L., Lublin F.D., Metz L.M., McFarland H.F., O’Connor P.W. (2005). Diagnostic criteria for multiple sclerosis: 2005 revisions to the “McDonald Criteria”. Ann. Neurol..

[64] Brownlee W.J., Vidal-Jordana A., Shatila M., Strijbis E., Schoof L., Killestein J., Barkhof F., Bollo L., Rovira A., Sastre-Garriga J. (2025). Towards a Unified Set of Diagnostic Criteria for Multiple Sclerosis. Ann. Neurol..

[65] Levraut M., Landes-Chateau C., Mondot L., Cohen M., Lebrun-Frenay C. (2025). The Kappa Free Light Chains Index and Central Vein Sign: Two New Biomarkers for Multiple Sclerosis Diagnosis. Neurol. Ther..

[66] Vidal-Jordana A., Rovira A., Calderon W., Arrambide G., Castilló J., Moncho D., Rahnama K., Collorone S., Toosy A.T., Ciccarelli O. (2024). Adding the Optic Nerve in Multiple Sclerosis Diagnostic Criteria: A Longitudinal, Prospective, Multicenter Study. Neurology.

[67] Sati P., Oh J., Constable R.T., Evangelou N., Guttmann C.R., Henry R.G., Klawiter E.C., Mainero C., Massacesi L., McFarland H. (2016). The central vein sign and its clinical evaluation for the diagnosis of multiple sclerosis: A consensus statement from the North American Imaging in Multiple Sclerosis Cooperative. Nat. Rev. Neurol..

[68] Maggi P., Absinta M., Grammatico M., Vuolo L., Emmi G., Carlucci G., Spagni G., Barilaro A., Repice A.M., Emmi L. (2018). Central vein sign differentiates Multiple Sclerosis from central nervous system inflammatory vasculopathies. Ann. Neurol..

[69] Absinta M., Sati P., Masuzzo F., Nair G., Sethi V., Kolb H., Ohayon J., Wu T., Cortese I.C.M., Reich D.S. (2019). Association of Chronic Active Multiple Sclerosis Lesions With Disability In Vivo. JAMA Neurol..

[70] Rjeily N.B., Solomon A.J. (2024). Correction to: Misdiagnosis of Multiple Sclerosis: Past, Present, and Future. Curr. Neurol. Neurosci. Rep..

[71] Sinnecker T., Clarke M.A., Meier D., Enzinger C., Calabrese M., De Stefano N., Pitiot A., Giorgio A., Schoonheim M.M., Paul F. (2019). Evaluation of the Central Vein Sign as a Diagnostic Imaging Biomarker in Multiple Sclerosis. JAMA Neurol..

[72] Adams C.W. (1988). Perivascular iron deposition and other vascular damage in multiple sclerosis. J. Neurol. Neurosurg. Psychiatry.

[73] Castellaro M., Tamanti A., Pisani A.I., Pizzini F.B., Crescenzo F., Calabrese M. (2020). The Use of the Central Vein Sign in the Diagnosis of Multiple Sclerosis: A Systematic Review and Meta-analysis. Diagnostics.

[74] Rocca M.A., Preziosa P., Filippi M. (2024). Juxtacortical Paramagnetic Rim: A New MRI Marker to Characterize Focal Cortical Pathology in Multiple Sclerosis?. Neurology.

[75] Dal-Bianco A., Grabner G., Kronnerwetter C., Weber M., Höftberger R., Berger T., Auff E., Leutmezer F., Trattnig S., Lassmann H. (2017). Slow expansion of multiple sclerosis iron rim lesions: Pathology and 7 T magnetic resonance imaging. Acta Neuropathol..

[76] Maggi P., Sati P., Nair G., Cortese I.C.M., Jacobson S., Smith B.R., Nath A., Ohayon J., van Pesch V., Perrotta G. (2020). Paramagnetic Rim Lesions are Specific to Multiple Sclerosis: An International Multicenter 3T MRI Study. Ann. Neurol..

[77] Reeves J.A., Mohebbi M., Wicks T., Salman F., Bartnik A., Jakimovski D., Bergsland N., Schweser F., Weinstock-Guttman B., Dwyer M.G. (2024). Paramagnetic rim lesions predict greater long-term relapse rates and clinical progression over 10 years. Mult. Scler. J..

[78] Elliott C., Belachew S., Wolinsky J.S., Hauser S.L., Kappos L., Barkhof F., Bernasconi C., Fecker J., Model F., Wei W. (2019). Chronic white matter lesion activity predicts clinical progression in primary progressive multiple sclerosis. Brain.

[79] Elliott C., Rudko D.A., Arnold D.L., Fetco D., Elkady A.M., Araujo D., Zhu B., Gafson A., Tian Z., Belachew S. (2023). Lesion-level correspondence and longitudinal properties of paramagnetic rim and slowly expanding lesions in multiple sclerosis. Mult. Scler. J..

[80] Sanchez-Dalmau B., Martinez-Lapiscina E.H., Torres-Torres R., Ortiz-Perez S., Zubizarreta I., Pulido-Valdeolivas I.V., Alba-Arbalat S., Guerrero-Zamora A., Calbet D., Villoslada P. (2018). Early retinal atrophy predicts long-term visual impairment after acute optic neuritis. Mult. Scler. J..

[81] Lee T.H., Ji Y.S., Park S.W., Heo H. (2017). Retinal ganglion cell and axonal loss in optic neuritis: Risk factors and visual functions. Eye.

[82] Xu S.C., Kardon R.H., Leavitt J.A., Flanagan E.P., Pittock S.J., Chen J.J. (2019). Optical coherence tomography is highly sensitive in detecting prior optic neuritis. Neurology.

[83] Bsteh G., Hegen H., Altmann P., Auer M., Berek K., Zinganell A., Pauli F.D., Rommer P., Deisenhammer F., Leutmezer F. (2020). Validation of inter-eye difference thresholds in optical coherence tomography for identification of optic neuritis in multiple sclerosis. Mult. Scler. Relat. Disord..

[84] Saidha S., Naismith R.T. (2019). Optical coherence tomography for diagnosing optic neuritis. Neurology.

[85] Andorrà M., Alba-Arbalat S., Camos-Carreras A., Gabilondo I., Fraga-Pumar E., Torres-Torres R., Pulido-Valdeolivas I., Tercero-Uribe A.I., Guerrero-Zamora A.M., Ortiz-Perez S. (2020). Using Acute Optic Neuritis Trials to Assess Neuroprotective and Remyelinating Therapies in Multiple Sclerosis. JAMA Neurol..

[86] Presslauer S., Milosavljevic D., Brücke T., Bayer P., Hübl W. (2008). Elevated levels of kappa free light chains in CSF support the diagnosis of multiple sclerosis. J. Neurol..

[87] Leurs C., Twaalfhoven H., Lissenberg-Witte B., van Pesch V., Dujmovic I., Drulovic J., Castellazzi M., Bellini T., Pugliatti M., Kuhle J. (2020). Kappa free light chains is a valid tool in the diagnostics of MS: A large multicenter study. Mult. Scler. J..

[88] Hegen H., Arrambide G., Gnanapavan S., Kaplan B., Khalil M., Saadeh R., Teunissen C., Tumani H., Villar L.M., Willrich M.A.V. (2023). Cerebrospinal fluid kappa free light chains for the diagnosis of multiple sclerosis: A consensus statement. Mult. Scler. J..

[89] Nabizadeh F., Mohammadi M., Maleki T., Valizadeh P., Sodeifian F. (2024). Diagnostic value of kappa free light chain and kappa index in Multiple Sclerosis: A systematic review and meta-analysis. Neurol. Lett..

[90] Maroto-García J., Mañez M., Martínez-Escribano A., Hachmaoui-Ridaoui A., Ortiz C., Ábalos-García C., Gónzález I., García de la Torre Á., Ruiz-Galdón M. (2024). A sex-dependent algorithm including kappa free light chain for multiple sclerosis diagnosis. Scand. J. Immunol..

[91] Beck R.W., Trobe J.D. (1995). What we have learned from the Optic Neuritis Treatment Trial. Ophthalmology.

[92] Konen F.F., Schwenkenbecher P., Jendretzky K.F., Gingele S., Sühs K.-W., Tumani H., Süße M., Skripuletz T. (2021). The Increasing Role of Kappa Free Light Chains in the Diagnosis of Multiple Sclerosis. Cells.

[93] Keltner J.L., Johnson C.A., Spurr J.O., Beck R.W. (1994). Visual field profile of optic neuritis. One-year follow-up in the Optic Neuritis Treatment Trial. Arch. Ophthalmol..

[94] Balcer L.J. (2006). Clinical practice. Optic neuritis. N. Engl. J. Med..

[95] Hickman S.J., Ko M., Chaudhry F., Jay W.M., Plant G.T. (2008). Optic Neuritis: An Update Typical and Atypical Optic Neuritis. Neuro-Ophthalmol..

[96] Ambika S., Lakshmi P. (2024). Infectious optic neuropathy (ION), how to recognise it and manage it. Eye.

[97] Kahloun R., Abroug N., Ksiaa I., Mahmoud A., Zeghidi H., Zaouali S., Khairallah M. (2015). Infectious optic neuropathies: A clinical update. Eye Brain.

[98] Lennon V.A., Wingerchuk D.M., Kryzer T.J., Pittock S.J., Lucchinetti C.F., Fujihara K., Nakashima I., Weinshenker B.G. (2004). A serum autoantibody marker of neuromyelitis optica: Distinction from multiple sclerosis. Lancet.

[99] Cree B.A.C., Bennett J.L., Kim H.J., Weinshenker B.G., Pittock S.J., Wingerchuk D.M., Fujihara K., Paul F., Cutter G.R., Marignier R. (2019). Inebilizumab for the treatment of neuromyelitis optica spectrum disorder (N-MOmentum): A double-blind, randomised placebo-controlled phase 2/3 trial. Lancet.

[100] Jurynczyk M., Messina S., Woodhall M.R., Raza N., Everett R., Roca-Fernandez A., Tackley G., Hamid S., Sheard A., Reynolds G. (2017). Clinical presentation and prognosis in MOG-antibody disease: A UK study. Brain.

[101] Cross S.A., Salomao D.R., Parisi J.E., Kryzer T.J., Bradley E.A., Mines J.A., Lam B.L., Lennon V.A. (2003). Paraneoplastic autoimmune optic neuritis with retinitis defined by CRMP-5-IgG. Ann. Neurol..

[102] Cohen D.A., Bhatti M.T., Pulido J.S., Lennon V.A., Dubey D., Flanagan E.P., Pittock S.J., Klein C.J., Chen J.J. (2020). Collapsin Response-Mediator Protein 5-Associated Retinitis, Vitritis, and Optic Disc Edema. Ophthalmology.

[103] Greco G., Masciocchi S., Diamanti L., Bini P., Vegezzi E., Marchioni E., Colombo E., Rigoni E., Businaro P., Ferraro O.E. (2023). Visual System Involvement in Glial Fibrillary Acidic Protein Astrocytopathy: Two Case Reports and a Systematic Literature Review. Neurol. Neuroimmunol. Neuroinflamm..

[104] Jia N., Wang J., He Y., Li Z., Lai C. (2023). Isolated optic neuritis with positive glial fibrillary acidic protein antibody. BMC Ophthalmol..

[105] White D., Mollan S.P., Ramalingam S., Nagaraju S., Hayton T., Jacob S. (2019). Enlarged and Enhancing Optic Nerves in Advanced Glial Fibrillary Acidic Protein Meningoencephalomyelitis. J. Neuro-Ophthalmol. Off. J. N. Am. Neuro-Ophthalmol. Soc..

[106] Ke G., Jian S., Yang T., Zhao X. (2024). Clinical characteristics and MRI features of autoimmune glial fibrillary acidic protein astrocytopathy: A case series of 34 patients. Front. Neurol..

[107] Flanagan E.P., Hinson S.R., Lennon V.A., Fang B., Aksamit A.J., Morris P.P., Basal E., Honorat J.A., Alfugham N.B., Linnoila J.J. (2017). Glial fibrillary acidic protein immunoglobulin G as biomarker of autoimmune astrocytopathy: Analysis of 102 patients. Ann. Neurol..

[108] Martinez-Hernandez E., Sepulveda M., Rostásy K., Höftberger R., Graus F., Harvey R.J., Saiz A., Dalmau J. (2015). Antibodies to aquaporin 4, myelin-oligodendrocyte glycoprotein, and the glycine receptor α1 subunit in patients with isolated optic neuritis. JAMA Neurol..

[109] Piquet A.L., Khan M., Warner J.E.A., Wicklund M.P., Bennett J.L., Leehey M.A., Seeberger L., Schreiner T.L., Paz Soldan M.M., Clardy S.L. (2019). Novel clinical features of glycine receptor antibody syndrome: A series of 17 cases. Neurol. Neuroimmunol. Neuroinflamm..

[110] Hamid S., Elsone L., Waters P., Woodhall M.W., Mutch K., Rafferty C., Tang L., Solomon T., Vincent A., Jacob A. (2015). Glycine receptor antibody—A marker for nmo/non-ms demyelination?. J. Neurol. Neurosurg. Psychiatry.

[111] Melzi S., Aboura R., Ladjouze A., Bouhafs N., Boulesnane K., Bouadjar R., Mebrouki L., Beddek Z., Haddad N., Tabti M. (2022). 66 Optic neuritis in children. Rheumatology.

[112] Sun C.B., Ma Z., Liu Z. (2023). Case Report: Severe Optic Neuritis after Multiple Episodes of Malaria in a Traveler to Africa. Am. J. Trop. Med. Hyg..

[113] Kerrison J.B., Lounsbury D., Thirkill C.E., Lane R.G., Schatz M.P., Engler R.M. (2002). Optic neuritis after anthrax vaccination. Ophthalmology.

[114] Shukla P., Sharma N., Shaia J.K., Cohen D.A., Singh R.P., Talcott K.E. (2024). The Risk of Optic Neuritis following mRNA Coronavirus Disease 2019 Vaccination Compared to Coronavirus Disease 2019 Infection and Other Vaccinations. Ophthalmology.

[115] Stübgen J.P. (2013). A literature review on optic neuritis following vaccination against virus infections. Autoimmun. Rev..

[116] Karussis D., Petrou P. (2014). The spectrum of post-vaccination inflammatory CNS demyelinating syndromes. Autoimmun. Rev..

[117] Matiello M., Lennon V.A., Jacob A., Pittock S.J., Lucchinetti C.F., Wingerchuk D.M., Weinshenker B.G. (2008). NMO-IgG predicts the outcome of recurrent optic neuritis. Neurology.

[118] Benoilid A., Tilikete C., Collongues N., Arndt C., Vighetto A., Vignal C., de Seze J. (2014). Relapsing optic neuritis: A multicentre study of 62 patients. Mult. Scler..

[119] Chalmoukou K., Alexopoulos H., Akrivou S., Stathopoulos P., Reindl M., Dalakas M.C. (2015). Anti-MOG antibodies are frequently associated with steroid-sensitive recurrent optic neuritis. Neurol. Neuroimmunol. Neuroinflamm..

[120] Kidd D., Burton B., Plant G.T., Graham E.M. (2003). Chronic relapsing inflammatory optic neuropathy (CRION). Brain.

[121] Petzold A., Plant G.T. (2014). Chronic relapsing inflammatory optic neuropathy: A systematic review of 122 cases reported. J. Neurol..

[122] Lee H.J., Kim B., Waters P., Woodhall M., Irani S., Ahn S., Kim S.J., Kim S.M. (2018). Chronic relapsing inflammatory optic neuropathy (CRION): A manifestation of myelin oligodendrocyte glycoprotein antibodies. J. Neuroinflamm..

[123] Bennett J.L., Costello F., Chen J.J., Petzold A., Biousse V., Newman N.J., Galetta S.L. (2023). Optic neuritis and autoimmune optic neuropathies: Advances in diagnosis and treatment. Lancet Neurol..

[124] Li H., Zhou H., Sun J., Wang H., Wang Y., Wang Z., Li J. (2020). Optic Perineuritis and Its Association With Autoimmune Diseases. Front. Neurol..

[125] Ma K.S., Lee C.M., Chen P.H., Yang Y., Dong Y.W., Wang Y.H., Wei J.C., Zheng W.J. (2022). Risk of Autoimmune Diseases Following Optic Neuritis: A Nationwide Population-Based Cohort Study. Front. Med..

[126] Kim S.M., Woodhall M.R., Kim J.S., Kim S.J., Park K.S., Vincent A., Lee K.W., Waters P. (2015). Antibodies to MOG in adults with inflammatory demyelinating disease of the CNS. Neurol. Neuroimmunol. Neuroinflamm..

[127] Hickman S.J., Petzold A. (2022). Update on Optic Neuritis: An International View. Neuroophthalmology.

[128] Petzold A., Plant G.T. (2014). Diagnosis and classification of autoimmune optic neuropathy. Autoimmun. Rev..

[129] Toosy A.T., Mason D.F., Miller D.H. (2014). Optic neuritis. Lancet Neurol..

[130] Hickman S.J., Dalton C.M., Miller D.H., Plant G.T. (2002). Management of acute optic neuritis. Lancet.

[131] Terrim S., Silva G.D., de Sá e Benevides Falcao F.C., dos Reis Pereira C., de Souza Andrade Benassi T., Fortini I., Gonçalves M.R.R., Castro L.H.M., Comerlatti L.R., de Medeiros Rimkus C. (2023). Real-world application of the 2022 diagnostic criteria for first-ever episode of optic neuritis. J. Neuroimmunol..

[132] Klyscz P., Asseyer S., Alonso R., Bereuter C., Bialer O., Bick A., Carta S., Chen J.J., Cohen L., Cohen-Tayar Y. (2024). Application of the international criteria for optic neuritis in the Acute Optic Neuritis Network. Ann. Clin. Transl. Neurol..

[133] Ghezzi A., Martinelli V., Rodegher M., Zaffaroni M., Comi G. (2000). The prognosis of idiopathic optic neuritis. Neurol. Sci. Off. J. Ital. Neurol. Soc. Ital. Soc. Clin. Neurophysiol..

[134] Siuko M., Kivelä T.T., Setälä K., Tienari P.J. (2019). The clinical spectrum and prognosis of idiopathic acute optic neuritis: A longitudinal study in Southern Finland. Mult. Scler. Relat. Disord..

[135] Jendretzky K.F., Bajor A., Lezius L.M., Hümmert M.W., Konen F.F., Grosse G.M., Schwenkenbecher P., Sühs K.W., Trebst C., Framme C. (2024). Clinical and paraclinical characteristics of optic neuritis in the context of the McDonald criteria 2017. Sci. Rep..

[136] Lebranchu P., Mazhar D., Wiertlewski S., Le Meur G., Couturier J., Ducloyer J.B. (2025). One-year risk of multiple sclerosis after a first episode of optic neuritis according to modern diagnosis criteria. Mult. Scler. Relat. Disord..

[137] Huang Y., Liu Y., Liu Y., Li Q., Fu X., Zou L., Xu Q. (2022). Teaching NeuroImage: Rapid Identification of Infectious Optic Neuritis by Next-Generation Sequencing. Neurology.

[138] Chen J.J., Tobin W.O., Majed M., Jitprapaikulsan J., Fryer J.P., Leavitt J.A., Flanagan E.P., McKeon A., Pittock S.J. (2018). Prevalence of Myelin Oligodendrocyte Glycoprotein and Aquaporin-4-IgG in Patients in the Optic Neuritis Treatment Trial. JAMA Ophthalmol..

[139] Wingerchuk D.M., Lennon V.A., Lucchinetti C.F., Pittock S.J., Weinshenker B.G. (2007). The spectrum of neuromyelitis optica. Lancet Neurol..

[140] Filippatou A.G., Mukharesh L., Saidha S., Calabresi P.A., Sotirchos E.S. (2020). AQP4-IgG and MOG-IgG Related Optic Neuritis-Prevalence, Optical Coherence Tomography Findings, and Visual Outcomes: A Systematic Review and Meta-Analysis. Front. Neurol..

[141] Hassan M.B., Stern C., Flanagan E.P., Pittock S.J., Kunchok A., Foster R.C., Jitprapaikulsan J., Hodge D.O., Bhatti M.T., Chen J.J. (2020). Population-Based Incidence of Optic Neuritis in the Era of Aquaporin-4 and Myelin Oligodendrocyte Glycoprotein Antibodies. Am. J. Ophthalmol..

[142] Castro-Suarez S., Guevara-Silva E., Osorio-Marcatinco V., Alvarez-Toledo K., Meza-Vega M., Caparó-Zamalloa C. (2022). Clinical and paraclinical profile of neuromyelitis optic spectrum disorder in a peruvian cohort. Mult. Scler. Relat. Disord..

[143] Cam S., Gulec B., Tutuncu M., Saip S., Siva A., Uygunoglu U. (2022). Disease Characteristics of Seropositive Neuromyelitis Optica Spectrum Disorder in a Turkish Cohort. Neurology.

[144] Zhou H., Zhao S., Yin D., Chen X., Xu Q., Chen T., Li X., Wang J., Li H., Peng C. (2016). Optic neuritis: A 5-year follow-up study of Chinese patients based on aquaporin-4 antibody status and ages. J. Neurol..

[145] Arnett S., Chew S.H., Leitner U., Hor J.Y., Paul F., Yeaman M.R., Levy M., Weinshenker B.G., Banwell B.L., Fujihara K. (2024). Sex ratio and age of onset in AQP4 antibody-associated NMOSD: A review and meta-analysis. J. Neurol..

[146] Borisow N., Kleiter I., Gahlen A., Fischer K., Wernecke K.D., Pache F., Ruprecht K., Havla J., Krumbholz M., Kümpfel T. (2017). Influence of female sex and fertile age on neuromyelitis optica spectrum disorders. Mult. Scler..

[147] Kim S.H., Mealy M.A., Levy M., Schmidt F., Ruprecht K., Paul F., Ringelstein M., Aktas O., Hartung H.P., Asgari N. (2018). Racial differences in neuromyelitis optica spectrum disorder. Neurology.

[148] Lana-Peixoto M.A., Talim N.C., Pedrosa D., Macedo J.M., Santiago-Amaral J. (2021). Prevalence of neuromyelitis optica spectrum disorder in Belo Horizonte, Southeast Brazil. Mult. Scler. Relat. Disord..

[149] Hor J.Y., Asgari N., Nakashima I., Broadley S.A., Leite M.I., Kissani N., Jacob A., Marignier R., Weinshenker B.G., Paul F. (2020). Epidemiology of Neuromyelitis Optica Spectrum Disorder and Its Prevalence and Incidence Worldwide. Front. Neurol..

[150] Poisson K., Moeller K., Fisher K.S. (2023). Pediatric Neuromyelitis Optica Spectrum Disorder. Semin. Pediatr. Neurol..

[151] Sepúlveda M., Armangué T., Sola-Valls N., Arrambide G., Meca-Lallana J.E., Oreja-Guevara C., Mendibe M., Alvarez de Arcaya A., Aladro Y., Casanova B. (2016). Neuromyelitis optica spectrum disorders. Neurol. Neuroimmunol. Neuroinflamm..

[152] Papadopoulos M.C., Verkman A.S. (2012). Aquaporin 4 and neuromyelitis optica. Lancet Neurol..

[153] Hinson S.R., Pittock S.J., Lucchinetti C.F., Roemer S.F., Fryer J.P., Kryzer T.J., Lennon V.A. (2007). Pathogenic potential of IgG binding to water channel extracellular domain in neuromyelitis optica. Neurology.

[154] Jarius S., Paul F., Weinshenker B.G., Levy M., Kim H.J., Wildemann B. (2020). Neuromyelitis optica. Nat. Rev. Dis. Primers.

[155] Saadoun S., Papadopoulos M.C. (2015). Role of membrane complement regulators in neuromyelitis optica. Mult. Scler..

[156] Soerensen S.F., Wirenfeldt M., Wlodarczyk A., Moerch M.T., Khorooshi R., Arengoth D.S., Lillevang S.T., Owens T., Asgari N. (2021). An Experimental Model of Neuromyelitis Optica Spectrum Disorder-Optic Neuritis: Insights Into Disease Mechanisms. Front. Neurol..

[157] Chen X., Cheng L., Pan Y., Chen P., Luo Y., Li S., Zou W., Wang K. (2023). Different immunological mechanisms between AQP4 antibody-positive and MOG antibody-positive optic neuritis based on RNA sequencing analysis of whole blood. Front. Immunol..

[158] Cao Y., Yao W., Chen F. (2024). Brief research report: WGCNA-driven identification of histone modification genes as potential biomarkers in AQP4-Associated optic neuritis. Front. Genet..

[159] Yang M., Wu Y., Song H., Lai M., Li H., Sun M., Zhao J., Fu J., Xu X., Xie L. (2022). Vision Prognosis and Associated Factors of Optic Neuritis in Dependence of Glial Autoimmune Antibodies. Am. J. Ophthalmol..

[160] Tajfirouz D., Padungkiatsagul T., Beres S., Moss H.E., Pittock S., Flanagan E., Kunchok A., Shah S., Bhatti M.T., Chen J.J. (2022). Optic chiasm involvement in AQP-4 antibody-positive NMO and MOG antibody-associated disorder. Mult. Scler..

[161] Akaishi T., Takeshita T., Himori N., Takahashi T., Misu T., Ogawa R., Kaneko K., Fujimori J., Abe M., Ishii T. (2020). Rapid Administration of High-Dose Intravenous Methylprednisolone Improves Visual Outcomes After Optic Neuritis in Patients With AQP4-IgG-Positive NMOSD. Front. Neurol..

[162] Moon Y., Jang Y., Lee H.J., Kim S.M., Kim S.J., Jung J.H. (2022). Long-Term Visual Prognosis in Patients With Aquaporin-4-Immunoglobulin G-Positive Neuromyelitis Optica Spectrum Disorder. J. Neuro-Ophthalmol. Off. J. N. Am. Neuro-Ophthalmol. Soc..

[163] Min Y.G., Moon Y., Kwon Y.N., Lee B.J., Park K.A., Han J.Y., Han J., Lee H.J., Baek S.H., Kim B.J. (2024). Prognostic factors of first-onset optic neuritis based on diagnostic criteria and antibody status: A multicentre analysis of 427 eyes. J. Neurol. Neurosurg. Psychiatry.

[164] Ishikawa H., Kezuka T., Shikishima K., Yamagami A., Hiraoka M., Chuman H., Nakamura M., Hoshi K., Goseki T., Mashimo K. (2019). Epidemiologic and Clinical Characteristics of Optic Neuritis in Japan. Ophthalmology.

[165] Kümpfel T., Giglhuber K., Aktas O., Ayzenberg I., Bellmann-Strobl J., Häußler V., Havla J., Hellwig K., Hümmert M.W., Jarius S. (2024). Update on the diagnosis and treatment of neuromyelitis optica spectrum disorders (NMOSD)—Revised recommendations of the Neuromyelitis Optica Study Group (NEMOS). Part II: Attack therapy and long-term management. J. Neurol..

[166] Niino M., Isobe N., Araki M., Ohashi T., Okamoto T., Ogino M., Okuno T., Ochi H., Kawachi I., Shimizu Y. (2024). Clinical practice guidelines for multiple sclerosis, neuromyelitis optica spectrum disorder, and myelin oligodendrocyte glycoprotein antibody-associated disease 2023 in Japan. Mult. Scler. Relat. Disord..

[167] Paul F., Marignier R., Palace J., Arrambide G., Asgari N., Bennett J.L., Cree B.A.C., De Sèze J., Fujihara K., Kim H.J. (2023). International Delphi Consensus on the Management of AQP4-IgG+ NMOSD: Recommendations for Eculizumab, Inebilizumab, and Satralizumab. Neurol. Neuroimmunol. Neuroinflamm..

[168] Amaral J.M., Talim N., Kleinpaul R., Lana-Peixoto M.A. (2020). Optic neuritis at disease onset predicts poor visual outcome in neuromyelitis optica spectrum disorders. Mult. Scler. Relat. Disord..

[169] Ueki S., Hatase T., Kiyokawa M., Kawachi I., Saji E., Onodera O., Fukuchi T., Igarashi H. (2021). Visual outcome of aquaporin-4 antibody-positive optic neuritis with maintenance therapy. Jpn. J. Ophthalmol..

[170] Mader S., Gredler V., Schanda K., Rostasy K., Dujmovic I., Pfaller K., Lutterotti A., Jarius S., Di Pauli F., Kuenz B. (2011). Complement activating antibodies to myelin oligodendrocyte glycoprotein in neuromyelitis optica and related disorders. J. Neuroinflamm..

[171] Sato D.K., Callegaro D., Lana-Peixoto M.A., Waters P.J., de Haidar Jorge F.M., Takahashi T., Nakashima I., Apostolos-Pereira S.L., Talim N., Simm R.F. (2014). Distinction between MOG antibody-positive and AQP4 antibody-positive NMO spectrum disorders. Neurology.

[172] Dinoto A., Cacciaguerra L., Vorasoot N., Redenbaugh V., Lopez-Chiriboga S.A., Valencia-Sanchez C., Guo K., Thakolwiboon S., Horsman S.E., Syc-Mazurek S.B. (2025). Clinical Features and Factors Associated With Outcome in Late Adult-Onset Myelin Oligodendrocyte Glycoprotein Antibody-Associated Disease. Neurology.

[173] Ciotti J.R., Eby N.S., Wu G.F., Naismith R.T., Chahin S., Cross A.H. (2020). Clinical and laboratory features distinguishing MOG antibody disease from multiple sclerosis and AQP4 antibody-positive neuromyelitis optica. Mult. Scler. Relat. Disord..

[174] Hor J.Y., Fujihara K. (2023). Epidemiology of myelin oligodendrocyte glycoprotein antibody-associated disease: A review of prevalence and incidence worldwide. Front. Neurol..

[175] Ferilli M.A.N., Valeriani M., Papi C., Papetti L., Ruscitto C., Figà Talamanca L., Ursitti F., Moavero R., Vigevano F., Iorio R. (2021). Clinical and neuroimaging characteristics of MOG autoimmunity in children with acquired demyelinating syndromes. Mult. Scler. Relat. Disord..

[176] Nair A., Sankhyan N., Sukhija J., Saini A.G., Vyas S., Suthar R., Sahu J.K., Rawat A. (2024). Clinical outcomes and Anti-MOG antibodies in pediatric optic neuritis: A prospective observational study. Eur. J. Paediatr. Neurol..

[177] Armangue T., Olivé-Cirera G., Martínez-Hernandez E., Sepulveda M., Ruiz-Garcia R., Muñoz-Batista M., Ariño H., González-Álvarez V., Felipe-Rucián A., Jesús Martínez-González M. (2020). Associations of paediatric demyelinating and encephalitic syndromes with myelin oligodendrocyte glycoprotein antibodies: A multicentre observational study. Lancet Neurol..

[178] de Mol C.L., Wong Y., van Pelt E.D., Wokke B., Siepman T., Neuteboom R.F., Hamann D., Hintzen R.Q. (2020). The clinical spectrum and incidence of anti-MOG-associated acquired demyelinating syndromes in children and adults. Mult. Scler..

[179] O’Connell K., Hamilton-Shield A., Woodhall M., Messina S., Mariano R., Waters P., Ramdas S., Leite M.I., Palace J. (2020). Prevalence and incidence of neuromyelitis optica spectrum disorder, aquaporin-4 antibody-positive NMOSD and MOG antibody-positive disease in Oxfordshire, UK. J. Neurol. Neurosurg. Psychiatry.

[180] Liu H., Zhou H., Wang J., Sun M., Teng D., Song H., Xu Q., Wei S. (2019). The prevalence and prognostic value of myelin oligodendrocyte glycoprotein antibody in adult optic neuritis. J. Neurol. Sci..

[181] Wendel E.M., Baumann M., Barisic N., Blaschek A., Coelho de Oliveira Koch E., Della Marina A., Diepold K., Hackenberg A., Hahn A., von Kalle T. (2020). High association of MOG-IgG antibodies in children with bilateral optic neuritis. Eur. J. Paediatr. Neurol..

[182] Jarius S., Ruprecht K., Kleiter I., Borisow N., Asgari N., Pitarokoili K., Pache F., Stich O., Beume L.A., Hümmert M.W. (2016). MOG-IgG in NMO and related disorders: A multicenter study of 50 patients. Part 2: Epidemiology, clinical presentation, radiological and laboratory features, treatment responses, and long-term outcome. J. Neuroinflamm..

[183] Chen J.J., Flanagan E.P., Jitprapaikulsan J., López-Chiriboga A.S.S., Fryer J.P., Leavitt J.A., Weinshenker B.G., McKeon A., Tillema J.M., Lennon V.A. (2018). Myelin Oligodendrocyte Glycoprotein Antibody-Positive Optic Neuritis: Clinical Characteristics, Radiologic Clues, and Outcome. Am. J. Ophthalmol..

[184] Virupakshaiah A., Schoeps V.A., Race J., Waltz M., Sharayah S., Nasr Z., Moseley C.E., Zamvil S.S., Gaudioso C., Schuette A. (2024). Predictors of a relapsing course in myelin oligodendrocyte glycoprotein antibody-associated disease. J. Neurol. Neurosurg. Psychiatry.

[185] Boudjani H., Fadda G., Dufort G., Antel J., Giacomini P., Levesque-Roy M., Oskoui M., Duquette P., Prat A., Girard M. (2023). Clinical course, imaging, and pathological features of 45 adult and pediatric cases of myelin oligodendrocyte glycoprotein antibody-associated disease. Mult. Scler. Relat. Disord..

[186] Cacciaguerra L., Sechi E., Komla-Soukha I., Chen J.J., Smith C.Y., Jenkins S.M., Guo K., Redenbaugh V., Fryer J.P., Tillema J.M. (2025). MOG antibody-associated disease epidemiology in Olmsted County, USA, and Martinique. J. Neurol..

[187] Tan K., Yeo T., Yong K.P., Thomas T., Wang F., Tye J., Quek A.M.L. (2021). Central nervous system inflammatory demyelinating diseases and neuroimmunology in Singapore—Epidemiology and evolution of an emerging subspecialty. Neurol. Clin. Neurosci..

[188] Höftberger R., Guo Y., Flanagan E.P., Lopez-Chiriboga A.S., Endmayr V., Hochmeister S., Joldic D., Pittock S.J., Tillema J.M., Gorman M. (2020). The pathology of central nervous system inflammatory demyelinating disease accompanying myelin oligodendrocyte glycoprotein autoantibody. Acta Neuropathol..

[189] Banwell B., Bennett J.L., Marignier R., Kim H.J., Brilot F., Flanagan E.P., Ramanathan S., Waters P., Tenembaum S., Graves J.S. (2023). Diagnosis of myelin oligodendrocyte glycoprotein antibody-associated disease: International MOGAD Panel proposed criteria. Lancet Neurol..

[190] Shor N., Aboab J., Maillart E., Lecler A., Bensa C., Le Guern G., Grunbaum S., Marignier R., Papeix C., Heron E. (2020). Clinical, imaging and follow-up study of optic neuritis associated with myelin oligodendrocyte glycoprotein antibody: A multicentre study of 62 adult patients. Eur. J. Neurol. Off. J. Eur. Fed. Neurol. Soc..

[191] Bruijstens A.L., Breu M., Wendel E.M., Wassmer E., Lim M., Neuteboom R.F., Wickström R., Baumann M., Bartels F., Finke C. (2020). E.U. paediatric MOG consortium consensus: Part 4—Outcome of paediatric myelin oligodendrocyte glycoprotein antibody-associated disorders. Eur. J. Paediatr. Neurol..

[192] Akaishi T., Himori N., Takeshita T., Misu T., Takahashi T., Takai Y., Nishiyama S., Fujimori J., Ishii T., Aoki M. (2021). Five-year visual outcomes after optic neuritis in anti-MOG antibody-associated disease. Mult. Scler. Relat. Disord..

[193] Havla J., Pakeerathan T., Schwake C., Bennett J.L., Kleiter I., Felipe-Rucián A., Joachim S.C., Lotz-Havla A.S., Kümpfel T., Krumbholz M. (2021). Age-dependent favorable visual recovery despite significant retinal atrophy in pediatric MOGAD: How much retina do you really need to see well?. J. Neuroinflamm..

[194] Fan Y., Wang Z., Wu Y., Zhou L., Wang L., Huang W., Tan H., Chang X., ZhangBao J., Quan C. (2025). Fewer relapses and worse outcomes of patients with late-onset myelin oligodendrocyte glycoprotein antibody-associated disease. J. Neurol. Neurosurg. Psychiatry.

[195] Zhao J., Chen X., Zhang J., Liu L., Wang J., Zhu L. (2024). Isolated myelin oligodendrocyte glycoprotein antibody-associated optic neuritis in adults: The importance of age of onset and prognosis-related radiological features. Mult. Scler. Relat. Disord..

[196] Dai Y., Yin Q., Han B., Yuan T., Su K., Dai W., Wang D., Yang L. (2025). MOG-IgG detection in serum and cerebrospinal fluid: Diagnostic implications and clinical correlates in adult-onset Chinese MOGAD patients. J. Neurol..

[197] Burton J.M., Youn S., Al-Ani A., Costello F. (2024). Patterns and utility of myelin oligodendrocyte glycoprotein (MOG) antibody testing in cerebrospinal fluid. J. Neurol..

[198] Takai Y., Misu T., Kaneko K., Chihara N., Narikawa K., Tsuchida S., Nishida H., Komori T., Seki M., Komatsu T. (2020). Myelin oligodendrocyte glycoprotein antibody-associated disease: An immunopathological study. Brain.

[199] Hyun J.W., Woodhall M.R., Kim S.H., Jeong I.H., Kong B., Kim G., Kim Y., Park M.S., Irani S.R., Waters P. (2017). Longitudinal analysis of myelin oligodendrocyte glycoprotein antibodies in CNS inflammatory diseases. J. Neurol. Neurosurg. Psychiatry.

[200] Jitprapaikulsan J., Chen J.J., Flanagan E.P., Tobin W.O., Fryer J.P., Weinshenker B.G., McKeon A., Lennon V.A., Leavitt J.A., Tillema J.M. (2018). Aquaporin-4 and Myelin Oligodendrocyte Glycoprotein Autoantibody Status Predict Outcome of Recurrent Optic Neuritis. Ophthalmology.

[201] Rode J., Pique J., Maarouf A., Ayrignac X., Bourre B., Ciron J., Cohen M., Collongues N., Deschamps R., Maillart E. (2023). Time to steroids impacts visual outcome of optic neuritis in MOGAD. J. Neurol. Neurosurg. Psychiatry.

[202] Bruijstens A.L., Wendel E.M., Lechner C., Bartels F., Finke C., Breu M., Flet-Berliac L., de Chalus A., Adamsbaum C., Capobianco M. (2020). E.U. paediatric MOG consortium consensus: Part 5—Treatment of paediatric myelin oligodendrocyte glycoprotein antibody-associated disorders. Eur. J. Paediatr. Neurol..

[203] Whittam D.H., Karthikeayan V., Gibbons E., Kneen R., Chandratre S., Ciccarelli O., Hacohen Y., de Seze J., Deiva K., Hintzen R.Q. (2020). Treatment of MOG antibody associated disorders: Results of an international survey. J. Neurol..

[204] Chen J.J., Flanagan E.P., Bhatti M.T., Jitprapaikulsan J., Dubey D., Lopez Chiriboga A.S.S., Fryer J.P., Weinshenker B.G., McKeon A., Tillema J.M. (2020). Steroid-sparing maintenance immunotherapy for MOG-IgG associated disorder. Neurology.

[205] Bilodeau P.A., Vishnevetsky A., Molazadeh N., Lotan I., Anderson M., Romanow G., Salky R., Healy B.C., Matiello M., Chitnis T. (2024). Effectiveness of immunotherapies in relapsing myelin oligodendrocyte glycoprotein antibody-associated disease. Mult. Scler..

[206] Chen J.J., Huda S., Hacohen Y., Levy M., Lotan I., Wilf-Yarkoni A., Stiebel-Kalish H., Hellmann M.A., Sotirchos E.S., Henderson A.D. (2022). Association of Maintenance Intravenous Immunoglobulin With Prevention of Relapse in Adult Myelin Oligodendrocyte Glycoprotein Antibody-Associated Disease. JAMA Neurol..

[207] Lu Q., Luo J., Hao H., Liu R., Jin H., Jin Y., Gao F. (2021). Efficacy and safety of long-term immunotherapy in adult patients with MOG antibody disease: A systematic analysis. J. Neurol..

[208] Nakajima H., Motomura M., Tanaka K., Fujikawa A., Nakata R., Maeda Y., Shima T., Mukaino A., Yoshimura S., Miyazaki T. (2015). Antibodies to myelin oligodendrocyte glycoprotein in idiopathic optic neuritis. BMJ Open.

[209] Zhao G., Chen Q., Huang Y., Li Z., Sun X., Lu P., Yan S., Wang M., Tian G. (2018). Clinical characteristics of myelin oligodendrocyte glycoprotein seropositive optic neuritis: A cohort study in Shanghai, China. J. Neurol..

[210] Ramanathan S., Prelog K., Barnes E., Tantsis E., Reddel S., Henderson A., Vucic O., Gorman M., Benson L., Alper G. (2016). Myelin Oligodendrocyte Glycoprotein Antibodies are Associated with Bilateral and Recurrent Optic Neuritis and have a Distinct Radiological Profile to Multiple Sclerosis or Aquaporin-4 Antibody-associated Optic Neuritis (P3.001). Neurology.

[211] Ramanathan S., Prelog K., Barnes E.H., Tantsis E.M., Reddel S.W., Henderson A.P., Vucic S., Gorman M.P., Benson L.A., Alper G. (2016). Radiological differentiation of optic neuritis with myelin oligodendrocyte glycoprotein antibodies, aquaporin-4 antibodies, and multiple sclerosis. Mult. Scler..

[212] Bianchi A., Cortese R., Prados F., Tur C., Kanber B., Yiannakas M.C., Samson R., De Angelis F., Magnollay L., Jacob A. (2024). Optic chiasm involvement in multiple sclerosis, aquaporin-4 antibody-positive neuromyelitis optica spectrum disorder and myelin oligodendrocyte glycoprotein-associated disease. Mult. Scler..

[213] Cortese R., Prados Carrasco F., Tur C., Bianchi A., Brownlee W., De Angelis F., De La Paz I., Grussu F., Haider L., Jacob A. (2023). Differentiating Multiple Sclerosis From AQP4-Neuromyelitis Optica Spectrum Disorder and MOG-Antibody Disease With Imaging. Neurology.

[214] Stiebel-Kalish H., Lotan I., Brody J., Chodick G., Bialer O., Marignier R., Bach M., Hellmann M.A. (2017). Retinal Nerve Fiber Layer May Be Better Preserved in MOG-IgG versus AQP4-IgG Optic Neuritis: A Cohort Study. PLoS ONE.

[215] Pache F., Zimmermann H., Mikolajczak J., Schumacher S., Lacheta A., Oertel F.C., Bellmann-Strobl J., Jarius S., Wildemann B., Reindl M. (2016). MOG-IgG in NMO and related disorders: A multicenter study of 50 patients. Part 4: Afferent visual system damage after optic neuritis in MOG-IgG-seropositive versus AQP4-IgG-seropositive patients. J. Neuroinflamm..

[216] Yu J., Huang Y., Zhou L., ZhangBao J., Zong Y., Quan C., Wang M. (2021). Comparison of the retinal vascular network and structure in patients with optic neuritis associated with myelin oligodendrocyte glycoprotein or aquaporin-4 antibodies: An optical coherence tomography angiography study. J. Neurol..

[217] Sotirchos E.S., Filippatou A., Fitzgerald K.C., Salama S., Pardo S., Wang J., Ogbuokiri E., Cowley N.J., Pellegrini N., Murphy O.C. (2020). Aquaporin-4 IgG seropositivity is associated with worse visual outcomes after optic neuritis than MOG-IgG seropositivity and multiple sclerosis, independent of macular ganglion cell layer thinning. Mult. Scler. J..

[218] Ramdani R., Pique J., Deschamps R., Ciron J., Maillart E., Audoin B., Cohen M., Zephir H., Laplaud D., Ayrignac X. (2025). Evaluation of the predictive value of CSF-restricted oligoclonal bands on residual disability and risk of relapse in adult patients with MOGAD: MOGADOC study. Mult. Scler..

[219] Jarius S., Lechner C., Wendel E.M., Baumann M., Breu M., Schimmel M., Karenfort M., Marina A.D., Merkenschlager A., Thiels C. (2020). Cerebrospinal fluid findings in patients with myelin oligodendrocyte glycoprotein (MOG) antibodies. Part 2: Results from 108 lumbar punctures in 80 pediatric patients. J. Neuroinflamm..

[220] Kovacs B., Lafferty T.L., Brent L.H., DeHoratius R.J. (2000). Transverse myelopathy in systemic lupus erythematosus: An analysis of 14 cases and review of the literature. Ann. Rheum. Dis..

[221] Smith C.A., Pinals R.S. (1982). Optic neuritis in systemic lupus erythematosus. J. Rheumatol..

[222] Kaneko K., Sato D.K., Nakashima I., Ogawa R., Akaishi T., Takai Y., Nishiyama S., Takahashi T., Misu T., Kuroda H. (2018). CSF cytokine profile in MOG-IgG+ neurological disease is similar to AQP4-IgG+ NMOSD but distinct from MS: A cross-sectional study and potential therapeutic implications. J. Neurol. Neurosurg. Psychiatry.

[223] Hoshina Y., Seay M., Vegunta S., Stulberg E.L., Wright M.A., Wong K.H., Smith T.L., Shimura D., Clardy S.L. (2025). Isolated Optic Neuritis: Etiology, Characteristics, and Outcomes in a US Mountain West Cohort. J. Neuro-Ophthalmol. Off. J. N. Am. Neuro-Ophthalmol. Soc..

[224] Hervas-Garcia J.V., Pagani-Cassara F. (2019). Chronic relapsing inflammatory optic neuropathy: A literature review. Rev. Neurol..

[225] Sun J.Y., Liu Z., Zhao P., Liu T. (2016). Optic neuritis as an initial presentation of primary Sjögren syndrome: A case report and literature review. Medicine.

[226] Fang B., McKeon A., Hinson S.R., Kryzer T.J., Pittock S.J., Aksamit A.J., Lennon V.A. (2016). Autoimmune Glial Fibrillary Acidic Protein Astrocytopathy: A Novel Meningoencephalomyelitis. JAMA Neurol..

[227] Akaishi T., Takahashi T., Fujihara K., Misu T., Fujimori J., Takai Y., Nishiyama S., Abe M., Ishii T., Aoki M. (2021). Impact of comorbid Sjögren syndrome in anti-aquaporin-4 antibody-positive neuromyelitis optica spectrum disorders. J. Neurol..

[228] Selbst R.G., Selhorst J.B., Harbison J.W., Myer E.C. (1983). Parainfectious Optic Neuritis: Report and Review Following Varicella. Arch. Neurol..

[229] Vianello F.A., Osnaghi S., Laicini E.A., Milani G.P., Tardini G., Cappellari A.M., Lunghi G., Agostoni C.V., Fossali E.F. (2014). Optic neuritis associated with influenza B virus meningoencephalitis. J. Clin. Virol..

[230] Bazzino Rubio F., Gonzalez Betlza M., Gonzalez Rabelino G., Bello Pedrosa O. (2017). Optic neuritis following Epstein-Barr virus encephalitis in immunocompetent children: A case report. Neurologia.

[231] Choi S.-Y., Choi Y.-J., Choi J.-H., Choi K.-D. (2017). Isolated optic neuritis associated with Mycoplasma pneumoniae infection: Report of two cases and literature review. Neurol. Sci..

[232] Hagbohm C., Ouellette R., Flanagan E.P., Jonsson D.I., Piehl F., Banwell B., Wickström R., Iacobaeus E., Granberg T., Ineichen B.V. (2024). Clinical and neuroimaging phenotypes of autoimmune glial fibrillary acidic protein astrocytopathy: A systematic review and meta-analysis. Eur. J. Neurol. Off. J. Eur. Fed. Neurol. Soc..

[233] Toledano-Illán C., Esparragosa Vázquez I., Zelaya Huerta M.V., Rosales Castillo J.J., Paternain Nuin A., Arbizu Lostao J., García de Eulate M.R., Riverol Fernández M. (2021). Autoimmune glial fibrillary acidic protein astrocytopathy: Case report of a treatable cause of rapidly progressive dementia. J. Neurol..

[234] Sechi E., Morris P.P., McKeon A., Pittock S.J., Hinson S.R., Weinshenker B.G., Aksamit A.J., Krecke K.N., Kaufmann T.J., Jolliffe E.A. (2019). Glial fibrillary acidic protein IgG related myelitis: Characterisation and comparison with aquaporin-4-IgG myelitis. J. Neurol. Neurosurg. Psychiatry.

[235] Kong Y., McKeon A., Koh O.S.Q., Chiew Y.R., Purohit B., Chin C.F., Zekeridou A., Ng A.S.L. (2021). Teaching NeuroImages: Linear Radial Periventricular Enhancement in Glial Fibrillary Acidic Protein Astrocytopathy. Neurology.

[236] Ahmed S., K V.C.C., Kannoth S., Raeesa F., Misri Z., Mascarenhas D.G., Nair S. (2025). Autoimmune GFAP Astrocytopathy-Beyond the Known Horizon, India’s First Multifaceted Institutional Experience. Ann. Neurosci..

[237] Kraker J.A., Chen J.J. (2023). An update on optic neuritis. J. Neurol..

[238] Dubey D., Lennon V.A., Gadoth A., Pittock S.J., Flanagan E.P., Schmeling J.E., McKeon A., Klein C.J. (2018). Autoimmune CRMP5 neuropathy phenotype and outcome defined from 105 cases. Neurology.

[239] Uysal S., Li Y., Thompson N., Conway D., Levin K., Kunchok A. (2023). Prognosticating Outcomes in Patients with CRMP5-IgG Associated Paraneoplastic Neurologic Disorders (P5-5.019). Neurology.

[240] Kidd D.P., Burton B.J., Graham E.M., Plant G.T. (2016). Optic neuropathy associated with systemic sarcoidosis. Neurol. Neuroimmunol. Neuroinflamm..

[241] Lin Y.-C., Wang A.-G., Yen M.-Y. (2009). Systemic lupus erythematosus-associated optic neuritis: Clinical experience and literature review. Acta Ophthalmol..

[242] Tang W.Q., Wei S.H. (2013). Primary Sjögren’s syndrome related optic neuritis. Int. J. Ophthalmol..

[243] Kardum Ž., Milas Ahić J., Lukinac A.M., Ivelj R., Prus V. (2021). Optic neuritis as a presenting feature of Behçet’s disease: Case-based review. Rheumatol. Int..

[244] Junek M., Khalidi N. (2023). ANCA-Associated Vasculitis For the Allergist and Immunologist: A Clinical Update. Can. Allergy Immunol. Today.

[245] Li J., Zhang Y., Zhou H., Wang L., Wang Z., Li H. (2019). Magnetic resonance imaging indicator of the causes of optic neuropathy in IgG4-related ophthalmic disease. BMC Med. Imaging.

[246] Vazquez-Romo K.A., Rodriguez-Hernandez A., Paczka J.A., Nuño-Suarez M.A., Rocha-Muñoz A.D., Zavala-Cerna M.G. (2017). Optic Neuropathy Secondary to Polyarteritis Nodosa, Case Report, and Diagnostic Challenges. Front. Neurol..

[247] Szydełko-Paśko U., Przeździecka-Dołyk J., Nowak Ł., Małyszczak A., Misiuk-Hojło M. (2023). Ocular Manifestations of Takayasu’s Arteritis—A Case-Based Systematic Review and Meta-Analysis. J. Clin. Med..

[248] Clemmensen K., Akrawi N., Stawowy M. (2015). Irreversible optic neuritis after infliximab treatment in a patient with ulcerative colitis. Scand. J. Gastroenterol..

[249] Suvajac G., Stojanovich L., Milenkovich S. (2007). Ocular manifestations in antiphospholipid syndrome. Autoimmun. Rev..

[250] Taga A., Wali A., Eberhart C.G., Green K.E. (2024). Teaching NeuroImage: Giant Cell Arteritis With Optic Perineuritis. Neurology.

[251] El Mayel A., Amor H.B., Sellem I., Mesfar R., Khochtali S., Ksiaa I., Khairallah M. (2022). Intermediate uveitis associated with Cogan’s syndrome. Acta Ophthalmol..

[252] Chou Y.-S., Lu D.-W., Chen J.-T. (2011). Ankylosing Spondylitis Presented as Unilateral Optic Neuritis in a Young Woman. Ocul. Immunol. Inflamm..

[253] Prasad N., Dubey A., Kumar K. (2024). Bilateral Optic Neuropathy in Association with Diffuse Cutaneous Systemic Sclerosis with Interstitial Lung Disease. Ocul. Immunol. Inflamm..

[254] Mansız-Kaplan B., Nacır B., Mülkoğlu C., Pervane-Vural S., Genç H. (2021). Undifferentiated connective tissue disease presenting with optic neuritis and concomitant axial spondyloarthritis: A rare case report. Turk. J. Phys. Med. Rehabil..

[255] Yousef N., Alhmood A., Mawri F., Kaddurah A., Diskin D., Abuhammour W. (2009). Bilateral optic neuritis in a patient with Kawasaki disease. J. Pediatr. Infect. Dis..

[256] Wilf-Yarkoni A., Zmira O., Tolkovsky A., Pflantzer B., Gofrit S.G., Kleffner I., Paul F., Dörr J. (2024). Clinical Characterization and Ancillary Tests in Susac Syndrome: A Systematic Review. Neurol. Neuroimmunol. Neuroinflamm..

[257] Della Zoppa M., Bertuccio F.R., Campo I., Tousa F., Crescenzi M., Lettieri S., Mariani F., Corsico A.G., Piloni D., Stella G.M. (2024). Phenotypes and Serum Biomarkers in Sarcoidosis. Diagnostics.

[258] Fritz D., van de Beek D., Brouwer M.C. (2016). Clinical features, treatment and outcome in neurosarcoidosis: Systematic review and meta-analysis. BMC Neurol..

[259] Ungprasert P., Tooley A.A., Crowson C.S., Matteson E.L., Smith W.M. (2019). Clinical Characteristics of Ocular Sarcoidosis: A Population-Based Study 1976–2013. Ocul. Immunol. Inflamm..

[260] Stern B.J., Royal W., Gelfand J.M., Clifford D.B., Tavee J., Pawate S., Berger J.R., Aksamit A.J., Krumholz A., Pardo C.A. (2018). Definition and Consensus Diagnostic Criteria for Neurosarcoidosis: From the Neurosarcoidosis Consortium Consensus Group. JAMA Neurol..

[261] Shen J., Lackey E., Shah S. (2023). Neurosarcoidosis: Diagnostic Challenges and Mimics A Review. Curr. Allergy Asthma Rep..

[262] Mochizuki M., Smith J.R., Takase H., Kaburaki T., Acharya N.R., Rao N.A. (2019). Revised criteria of International Workshop on Ocular Sarcoidosis (IWOS) for the diagnosis of ocular sarcoidosis. Br. J. Ophthalmol..

[263] Palejwala N.V., Walia H.S., Yeh S. (2012). Ocular manifestations of systemic lupus erythematosus: A review of the literature. Autoimmune Dis..

[264] Giorgi D., Balacco Gabrieli C. (1999). Optic neuropathy in systemic lupus erythematosus and antiphospholipid syndrome (APS): Clinical features, pathogenesis, review of the literature and proposed ophthalmological criteria for APS diagnosis. Clin. Rheumatol..

[265] Siatkowski R.M., Scott I.U., Verm A.M., Warn A.A., Farris B.K., Strominger M.B., Sklar E.M. (2001). Optic neuropathy and chiasmopathy in the diagnosis of systemic lupus erythematosus. J. Neuro-Ophthalmol. Off. J. N. Am. Neuro-Ophthalmol. Soc..

[266] Galindo-Rodríguez G., Aviña-Zubieta J.A., Pizarro S., Díaz de León V., Saucedo N., Fuentes M., Lavalle C. (1999). Cyclophosphamide pulse therapy in optic neuritis due to systemic lupus erythematosus: An open trial. Am. J. Med..

[267] Li H., Zhang Y., Yi Z., Huang D., Wei S. (2014). Frequency of autoantibodies and connective tissue diseases in Chinese patients with optic neuritis. PLoS ONE.

[268] Prasad C.B., Kopp C.R., Naidu G., Sharma V., Misra D.P., Agarwal V., Sharma A. (2024). Overlap syndrome of anti-aquaporin 4 positive neuromyelitis optica spectrum disorder and primary Sjögren’s syndrome: A systematic review of individual patient data. Rheumatol. Int..

[269] Lalji A., Izbudak I., Birnbaum J. (2017). Cortical blindness and not optic neuritis as a cause of vision loss in a Sjögren’s syndrome (SS) patient with the neuromyelitis optica spectrum disorder (NMOSD): Challenges of ascribing demyelinating syndromes to SS: A case report. Medicine.

[270] Zheng W., Liu X., Hou X., Zhu Y., Zhang T., Liao L. (2020). Recurrent optic neuritis in a patient with Sjogren syndrome and neuromyelitis optica spectrum disorder: A case report. Medicine.

[271] Ramos J.M., Tello A., Alzamora A., Ramón M.L. (2014). Optic Neuritis in a Traveler Returning From Dominican Republic to Spain With Dengue Virus Infection. J. Travel. Med..

[272] Farris B.K., Pickard D.J. (1990). Bilateral Postinfectious Optic Neuritis and Intravenous Steroid Therapy in Children. Ophthalmology.

[273] Zhao D., Li X., Carey A.R., Henderson A.D. (2024). Optic Neuritis and Cranial Neuropathies Diagnosis Rates before Coronavirus Disease 2019, in the Initial Pandemic Phase, and Post-Vaccine Introduction. Ophthalmology.

[274] Patel J., Alchaki A.R., Eddin M.F., Souayah N. (2018). Development of Optic Neuritis after Vaccination, A CDC/FDA Vaccine Adverse Event Reporting System (VAERS) Study, 1990–2017. (P2.146). Neurology.

[275] Han S.B., Hwang J.M., Kim J.S., Yang H.K. (2014). Optic neuritis following Varicella zoster vaccination: Report of two cases. Vaccine.

[276] Mutete F., Mwendaweli N., Asukile M., Mulubwa S., Mumbi W., Saylor D. (2023). MOG Antibody-Associated Bilateral Optic Neuritis Associated with Johnson and Johnson COVID-19 Vaccination (P12-3.009). Neurology.

[277] Baxter R., Lewis E., Fireman B., DeStefano F., Gee J., Klein N.P. (2016). Case-centered Analysis of Optic Neuritis After Vaccines. Clin. Infect. Dis..

[278] Korematsu S., Miyahara H., Kakita A., Izumi T. (2014). Elevated serum anti-phosphatidylcholine IgG antibodies in patients with influenza vaccination-associated optic neuritis. Vaccine.

[279] Georganta I., Chasapi D., Smith C.J., Kopsidas K., Tatham A. (2023). Systematic review exploring the clinical features of optic neuritis after SARS-CoV infection and vaccination. BMJ Open Ophthalmol..

[280] Hurissi E.A., Abuallut I.I., Dibaji M.Q., Jaly A., Alhazmi A.H., Abuageelah B.M., Alameer K.M., Alyami Y.M. (2024). Ocular Complications after COVID-19 Vaccination: A Systematic Review. Medicina.

[281] Chang H., Lee H.L., Yeo M., Kim J.S., Shin D.I., Lee S.S., Lee S.H. (2016). Recurrent optic neuritis and neuromyelitis optica-IgG following first and second human papillomavirus vaccinations. Clin. Neurol. Neurosurg..

[282] Yang X., Li X., Lai M., Wang J., Tan S., Chan H.H. (2022). Pain Symptoms in Optic Neuritis. Front. Pain. Res..

[283] Fazzone H.E., Lefton D.R., Kupersmith M.J. (2003). Optic neuritis: Correlation of pain and magnetic resonance imaging. Ophthalmology.

[284] Asseyer S., Hamblin J., Messina S., Mariano R., Siebert N., Everett R., Küker W., Bellmann-Strobl J., Ruprecht K., Jarius S. (2020). Prodromal headache in MOG-antibody positive optic neuritis. Mult. Scler. Relat. Disord..

[285] Tanaka K., Kezuka T., Ishikawa H., Tanaka M., Sakimura K., Abe M., Kawamura M. (2023). Pathogenesis, Clinical Features, and Treatment of Patients with Myelin Oligodendrocyte Glycoprotein (MOG) Autoantibody-Associated Disorders Focusing on Optic Neuritis with Consideration of Autoantibody-Binding Sites: A Review. Int. J. Mol. Sci..

[286] Huda S., Whittam D., Bhojak M., Chamberlain J., Noonan C., Jacob A. (2019). Neuromyelitis optica spectrum disorders. Clin. Med..

[287] Rosenkranz S.C., Kaulen B., Zimmermann H.G., Bittner A.K., Dorr M., Stellmann J.P. (2021). Validation of Computer-Adaptive Contrast Sensitivity as a Tool to Assess Visual Impairment in Multiple Sclerosis Patients. Front. Neurosci..

[288] Park S.-H., Park C.Y., Shin Y.J., Jeong K.S., Kim N.-H. (2020). Low Contrast Visual Acuity Might Help to Detect Previous Optic Neuritis. Front. Neurol..

[289] Fernandez V.C., Villa A.M. (2022). Acute optic neuritis: What do complementary tests add to diagnosis?. Mult. Scler. Relat. Disord..

[290] Laviers H., Petzold A., Braithwaite T. (2024). How far should I manage acute optic neuritis as an ophthalmologist? A United Kingdom perspective. Eye.

[291] Nguyen M.N.L., Zhu C., Kolbe S.C., Butzkueven H., White O.B., Fielding J., Kilpatrick T.J., Egan G.F., Klistorner A., van der Walt A. (2022). Early predictors of visual and axonal outcomes after acute optic neuritis. Front. Neurol..

[292] Villoslada P., Solana E., Alba-Arbalat S., Martinez-Heras E., Vivo F., Lopez-Soley E., Calvi A., Camos-Carreras A., Dotti-Boada M., Bailac R.A. (2024). Retinal Damage and Visual Network Reconfiguration Defines Visual Function Recovery in Optic Neuritis. Neurol. Neuroimmunol. Neuroinflamm..

[293] Hickman S.J., Toosy A.T., Miszkiel K.A., Jones S.J., Altmann D.R., MacManus D.G., Plant G.T., Thompson A.J., Miller D.H. (2004). Visual recovery following acute optic neuritis--a clinical, electrophysiological and magnetic resonance imaging study. J. Neurol..

[294] Kupersmith M.J., Alban T., Zeiffer B., Lefton D. (2002). Contrast-enhanced MRI in acute optic neuritis: Relationship to visual performance. Brain.

[295] Tur C., Goodkin O., Altmann D.R., Jenkins T.M., Miszkiel K., Mirigliani A., Fini C., Gandini Wheeler-Kingshott C.A., Thompson A.J., Ciccarelli O. (2016). Longitudinal evidence for anterograde trans-synaptic degeneration after optic neuritis. Brain.

[296] Mealy M., Whetstone A., Calabresi P., Levy M. (2014). Differentiating NMO and MS-Associated Optic Neuritis by MRI (S63.002). Neurology.

[297] Kitley J., Waters P., Woodhall M., Leite M.I., Murchison A., George J., Kuker W., Chandratre S., Vincent A., Palace J. (2014). Neuromyelitis Optica Spectrum Disorders With Aquaporin-4 and Myelin-Oligodendrocyte Glycoprotein Antibodies A Comparative Study. JAMA Neurol..

[298] Leitner U., Chew S.H., Blanch J., Viswanathan M., Patil S., Ward K., Bhuta S., Zhang P., Sun J., Broadley S.A. (2025). Characteristics of MRI lesions in AQP4 antibody-positive NMOSD, MOGAD, and multiple sclerosis: A systematic review and meta-analysis. J. Neurol..

[299] Jeyakumar N., Lerch M., Dale R.C., Ramanathan S. (2024). MOG antibody-associated optic neuritis. Eye.

[300] Baumann M., Bartels F., Finke C., Adamsbaum C., Hacohen Y., Rostásy K.E. (2020). U. paediatric MOG consortium consensus: Part 2—Neuroimaging features of paediatric myelin oligodendrocyte glycoprotein antibody-associated disorders. Eur. J. Paediatr. Neurol..

[301] Akaishi T., Sato D.K., Nakashima I., Takeshita T., Takahashi T., Doi H., Kurosawa K., Kaneko K., Kuroda H., Nishiyama S. (2016). MRI and retinal abnormalities in isolated optic neuritis with myelin oligodendrocyte glycoprotein and aquaporin-4 antibodies: A comparative study. J. Neurol. Neurosurg. Psychiatry.

[302] Messina S., Mariano R., Roca-Fernandez A., Cavey A., Jurynczyk M., Leite M.I., Calabrese M., Jenkinson M., Palace J. (2022). Contrasting the brain imaging features of MOG-antibody disease, with AQP4-antibody NMOSD and multiple sclerosis. Mult. Scler..

[303] Ciotti J.R., Eby N.S., Brier M.R., Wu G.F., Chahin S., Cross A.H., Naismith R.T. (2022). Central vein sign and other radiographic features distinguishing myelin oligodendrocyte glycoprotein antibody disease from multiple sclerosis and aquaporin-4 antibody-positive neuromyelitis optica. Mult. Scler..

[304] Petzold A., Chua S.Y.L., Khawaja A.P., Keane P.A., Khaw P.T., Reisman C., Dhillon B., Strouthidis N.G., Foster P.J., Patel P.J. (2021). Retinal asymmetry in multiple sclerosis. Brain.

[305] Oertel F.C., Specovius S., Zimmermann H.G., Chien C., Motamedi S., Bereuter C., Cook L., Lana Peixoto M.A., Fontanelle M.A., Kim H.J. (2021). Retinal Optical Coherence Tomography in Neuromyelitis Optica. Neurol. Neuroimmunol. Neuroinflamm..

[306] Huang L., Wang Y., Zhang R. (2023). Retina thickness in clinically affected and unaffected eyes in patients with aquaporin-4 immunoglobulin G antibody seropositive neuromyelitis optica spectrum disorders: A systematic review and meta-analysis. J. Neurol..

[307] Lu A., Zimmermann H.G., Specovius S., Motamedi S., Chien C., Bereuter C., Lana-Peixoto M.A., Fontenelle M.A., Ashtari F., Kafieh R. (2022). Astrocytic outer retinal layer thinning is not a feature in AQP4-IgG seropositive neuromyelitis optica spectrum disorders. J. Neurol. Neurosurg. Psychiatry.

[308] Oertel F.C., Zimmermann H.G., Motamedi S., Chien C., Aktas O., Albrecht P., Ringelstein M., Dcunha A., Pandit L., Martinez-Lapiscina E.H. (2023). Diagnostic value of intereye difference metrics for optic neuritis in aquaporin-4 antibody seropositive neuromyelitis optica spectrum disorders. J. Neurol. Neurosurg. Psychiatry.

[309] Barnes S., You Y., Shen T., Hardy T.A., Fraser C., Reddel S.W., Brilot F., Ramanathan S., Klistorner A., Yiannikas C. (2021). Structural and functional markers of optic nerve damage in myelin oligodendrocyte glycoprotein antibody-associated optic neuritis. Mult. Scler. J.—Exp. Transl. Clin..

[310] Vicini R., Brügger D., Abegg M., Salmen A., Grabe H.M. (2021). Differences in morphology and visual function of myelin oligodendrocyte glycoprotein antibody and multiple sclerosis associated optic neuritis. J. Neurol..

[311] Havla J., Kümpfel T., Schinner R., Spadaro M., Schuh E., Meinl E., Hohlfeld R., Outteryck O. (2017). Myelin-oligodendrocyte-glycoprotein (MOG) autoantibodies as potential markers of severe optic neuritis and subclinical retinal axonal degeneration. J. Neurol..

[312] Nolan R.C., Galetta S.L., Frohman T.C., Frohman E.M., Calabresi P.A., Castrillo-Viguera C., Cadavid D., Balcer L.J. (2018). Optimal Intereye Difference Thresholds in Retinal Nerve Fiber Layer Thickness for Predicting a Unilateral Optic Nerve Lesion in Multiple Sclerosis. J. Neuro-Ophthalmol. Off. J. N. Am. Neuro-Ophthalmol. Soc..

[313] Volpe G., Jurkute N., Girafa G., Zimmermann H.G., Motamedi S., Bereuter C., Pandit L., D’Cunha A., Yeaman M.R., Smith T.J. (2024). Diagnostic Value of Inter-Eye Difference Metrics on OCT for Myelin Oligodendrocyte Glycoprotein Antibody-Associated Optic Neuritis. Neurol. Neuroimmunol. Neuroinflamm..

[314] Ross R., Kenney R., Balcer L., Galetta S., Krupp L., O’Neill K., Marini C., Grossman S. (2024). Structure-function Correlates in Myelin Oligodendrocyte Glycoprotein Antibody Disease Optic Neuritis (P7-10.004). Neurology.

[315] Jarius S., Paul F., Aktas O., Asgari N., Dale R.C., de Seze J., Franciotta D., Fujihara K., Jacob A., Kim H.J. (2018). MOG encephalomyelitis: International recommendations on diagnosis and antibody testing. J. Neuroinflamm..

[316] Chen J.J., Sotirchos E.S., Henderson A.D., Vasileiou E.S., Flanagan E.P., Bhatti M.T., Jamali S., Eggenberger E.R., Dinome M., Frohman L.P. (2022). OCT retinal nerve fiber layer thickness differentiates acute optic neuritis from MOG antibody-associated disease and Multiple Sclerosis: RNFL thickening in acute optic neuritis from MOGAD vs MS. Mult. Scler. Relat. Disord..

[317] Deschamps R., Philibert M., Lamirel C., Lambert J., Vasseur V., Gueguen A., Bensa C., Lecler A., Marignier R., Vignal C. (2021). Visual field loss and structure-function relationships in optic neuritis associated with myelin oligodendrocyte glycoprotein antibody. Mult. Scler..

[318] Ramanathan S., Mohammad S., Tantsis E., Nguyen T.K., Merheb V., Fung V.S.C., White O.B., Broadley S., Lechner-Scott J., Vucic S. (2018). Clinical course, therapeutic responses and outcomes in relapsing MOG antibody-associated demyelination. J. Neurol. Neurosurg. Psychiatry.

[319] Oertel F.C., Sotirchos E.S., Zimmermann H.G., Motamedi S., Specovius S., Asseyer E.S., Chien C., Cook L., Vasileiou E., Filippatou A. (2022). Longitudinal Retinal Changes in MOGAD. Ann. Neurol..

[320] Specovius S., Zimmermann H.G., Oertel F.C., Chien C., Bereuter C., Cook L.J., Lana Peixoto M.A., Fontenelle M.A., Kim H.J., Hyun J.W. (2020). Cohort profile: A collaborative multicentre study of retinal optical coherence tomography in 539 patients with neuromyelitis optica spectrum disorders (CROCTINO). BMJ Open.

[321] Bollo L., Arrambide G., Cobo-Calvo A., Alvarez J.V., Alberich M., Cabello S., Castilló J., Galan I., Midaglia L.S., Acevedo B.R. (2024). Trans-Synaptic Degeneration in the Visual Pathway in Patients With Myelin Oligodendrocyte Glycoprotein Antibody-Associated Disease. Neurology.

[322] El Ayoubi N.K., Ismail A., Fahd F., Younes L., Chakra N.A., Khoury S.J. (2024). Retinal optical coherence tomography measures in multiple sclerosis: A systematic review and meta-analysis. Ann. Clin. Transl. Neurol..

[323] Laron M., Cheng H., Zhang B., Schiffman J.S., Tang R.A., Frishman L.J. (2010). Comparison of multifocal visual evoked potential, standard automated perimetry and optical coherence tomography in assessing visual pathway in multiple sclerosis patients. Mult. Scler. J..

[324] Fraser C., Klistorner A., Graham S., Garrick R., Billson F., Grigg J. (2006). Multifocal Visual Evoked Potential Latency Analysis: Predicting Progression to Multiple Sclerosis. Arch. Neurol..

[325] Park S.-H., Park C.-Y., Shin Y.J., Jeong K.S., Kim N.-H. (2022). Low Contrast Visual Evoked Potentials for Early Detection of Optic Neuritis. Front. Neurol..

[326] Shen T., You Y., Arunachalam S., Fontes A., Liu S., Gupta V., Parratt J., Wang C., Barnett M., Barton J. (2019). Differing Structural and Functional Patterns of Optic Nerve Damage in Multiple Sclerosis and Neuromyelitis Optica Spectrum Disorder. Ophthalmology.

[327] Britze J., Pihl-Jensen G., Frederiksen J.L. (2017). Retinal ganglion cell analysis in multiple sclerosis and optic neuritis: A systematic review and meta-analysis. J. Neurol..

[328] Petzold A., Wattjes M.P., Costello F., Flores-Rivera J., Fraser C.L., Fujihara K., Leavitt J., Marignier R., Paul F., Schippling S. (2014). The investigation of acute optic neuritis: A review and proposed protocol. Nat. Rev. Neurol..

[329] Tejeda-Velarde A., Costa-Frossard L., Sainz de la Maza S., Carrasco Á., Espiño M., Picón C., Toboso I., Walo P.E., Lourido D., Muriel A. (2018). Clinical usefulness of prognostic biomarkers in optic neuritis. Eur. J. Neurol..

[330] Krajnc N., Föttinger F., Ponleitner M., Kornek B., Leutmezer F., Macher S., Rommer P., Schmied C., Zebenholzer K., Zulehner G. (2025). Diagnostic accuracy of inter-eye difference of ganglion cell layer alone in identifying optic neuritis in multiple sclerosis. Mult. Scler..

[331] Huang-Link Y., Yang G., Gustafsson G., Gauffin H., Landtblom A.M., Mirabelli P., Link H. (2023). The Importance of Optical Coherence Tomography in the Diagnosis of Atypical or Subclinical Optic Neuritis: A Case Series Study. J. Clin. Med..

